# Supplementary material for: How good are GPs at adhering to a pragmatic trial protocol in primary care? Results from the ADDITION-Cambridge cluster-randomised pragmatic trial
Source: BMJ Open. 2018 Jun 14;8(6):e015295. doi: 10.1136/bmjopen-2016-015295 (PMC6009504; doi:10.1136/bmjopen-2016-015295)
Supplement: Supplementary file 1 [file bmjopen-2016-015295supp001.pdf]

Supplementary Material

Appendix 1: Proportion of patients receiving regular monitoring for HbA_{1c}, cholesterol and albuminuria and proportion of patients receiving blood pressure lowering medication

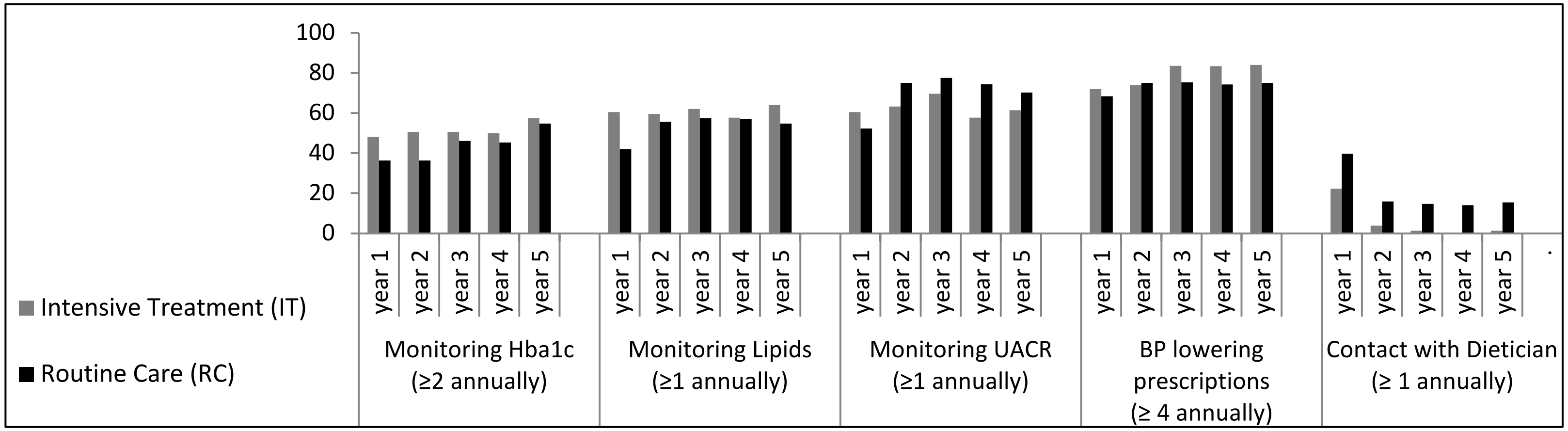

**Stratified by year ł**	**OR (95%-CI) ǂ**	**OR (95%-CI) ǂ**	**OR (95%-CI) ǂ**	**OR (95%-CI) #**	**OR (95%-CI) ǂ**
Year 1 (IT vs. RC)	2.00 (0.44, 9.02)	2.66 (0.53, 13.22)	1.41 (0.46, 4.32)	1.15 (0.55, 2.4)	0.88 (0.24, 3.26)
Year 2 (IT vs. RC)	2.29 (0.41, 12.63)	1.30 (0.23, 7.20)	0.62 (0.15, 2.60)	0.95 (0.45, 2.01)	0.38 (0.07, 2.18)
Year 3 (IT vs. RC)	1.28 (0.36, 4.52)	1.96 (0.36, 10.68)	0.93 (0.24, 3.56)	1.69 (0.72, 3.95)	0.12 (0.01, 1.39)
Year 4 (IT vs. RC)	1.52 (0.46, 5.03)	1.28 (0.29, 5.73)	0.49 (0.17, 1.45)	1.76 (0.72, 4.3)	-
Year 5 (IT vs. RC)	1.15 (0.44, 3.03)	2.3 (0.47, 11.32)	0.72 (0.30, 1.77)	1.89 (0.70, 5.15)	0.08 (0.01, 0.67)
**Year 1-5 ⊦**	**OR (95%-CI) ¬**	**OR (95%-CI) ¬**	**OR (95%-CI) ¬**	**OR (95%-CI) Ⅎ**	**OR (95%-CI) ¬**
Overall (IC vs. SC)	1.56 (0.63, 3.83)	1.53 (0.51, 4.6)	0.82 (0.34, 1.98)	1.41 (0.71, 2.8)	0.43 (0.32, 0.58)
Time since diagnosis (years)	1.12 (1.01, 1.23)	1.05 (0.95, 1.16)	1.08 (0.97, 1.2)	1.15 (1.02, 1.31)	0.13 (0.04, 0.45)
p-value (time x treatment)	0.294	0.303	0.075	0.223	0.001

ł stratified logistic regression models with a main effect for the intervention; adjusted for sex and age of diagnosis; accounted for patients being clustered within GP practices

⊦ overall logistic regression models with a main effect for the intervention and for time since diagnosis and an interaction term between intervention and time; adjusted for sex and age of diagnosis; accounted for patients being clustered within GP practices and observations being clustered in patients

ǂ n=169 in year 1, n=167 in year 2, n=168 in year 3, n=164 in year 4, and n=159 in year 5

n=151 in year1, n=149 in year 2, n=150 in year 3, n=146 in year 4 and n=141 in year 5

¬ n=827 observations (n=169 in year 1, n=167 in year 2, n=168 in year 3, n=164 in year 4, and n=159 in year 5)

Ⅎ n=737 observations (n=151 in year1, n=149 in year 2, n=150 in year 3, n=146 in year 4, and n=141 in year 5)

HbA_{1c} hemoglobin A1c; UACR urine-albumin-creatinine-ratio; BP blood pressure

Appendix 2: Results of various sensitivity analyses

	Adjusted odds ratio of having received 'continuous medication', IT vs. RC (reference)ǂ				Difference in adjusted mean number of contacts with GPs and nurses, IT vs. RC (reference) ⊦	
	**OR (95-% Cl)**				**adjusted mean difference (95%-Cl)**	
	Glucose-lowering	ACE-inhibiting	lipid-lowering	aspirin	# of GP contacts	# of nurse contacts
main model (from Figure 2 & 3)	*3.27 (1.81, 5.93)* ¬	*2.03 (1.13, 3.65)* ¬	*2.42 (1.30, 4.51)* ¬	*1.41 (0.61, 3.24)* ¬	*0.65 (-0.95, 2.26)*˧	*-0.15 (-1.77, 1.48)* ˧
a) weighted model	2.89 (1.51, 5.53) ¬	2.13 (1.15, 3.93) ¬	2.54 (1.32, 4.92) ¬	1.47 (0.59, 3.69) ¬	0.81 (-0.79, 2.42) ˧	0.21 (-1.40, 1.81) ˧
b) multiple imputed model	3.06 (1.78, 5.28) ǂ	2.05 (1.20, 3.50) ǂ	2.37 (1.32, 4.25) ǂ	1.32 (0.62, 2.80) ǂ	0.68 (-0.9, 2.26) ǂ	-0.10 (-1.70, 1.50) ǂ
c) threshold: ≥ 2 prescriptions annually	3.07 (1.68, 5.61) ¬	2.10 (1.12, 3.94) ¬	2.16 (1.13, 4.14) ¬	1.45 (0.70, 3.02) ¬	-	-
d) threshold: ≥ 6 prescriptions annually	3.97 (2.17, 7.26) ¬	2.24 (1.25, 4.03) ¬	2.35 (1.24, 4.45) ¬	1.40 (0.57, 3.46) ¬	-	-
e) threshold: ≥ 12 prescriptions annually	4.86 (2.34, 10.1) ¬	1.79 (0.79, 4.06) ¬	1.35 (0.58, 3.12) ¬	1.04 (0.37, 2.97) ¬	-	-

ł overall logistic regression models with a main effect for the intervention and for time since diagnosis and an interaction term between intervention and time; adjusted for sex and age of diagnosis; accounted for patients being clustered within GP practices and observations being clustered in patients

⊦ overall linear regression models with a main effect for the intervention and for time since diagnosis and an interaction term between intervention and time; adjusted for sex and age of diagnosis; accounted for patients being clustered within GP practices and observations being clustered in patients

¬ n=737 observations (n=151 in year1, n=149 in year 2, n=150 in year 3, n=146 in year 4, and n=141 in year 5)
ǂ n=885 observations (n=173 from year1 to year 5)

a) individuals weighted by the inverse probability of being in the sample given the status on the primary endpoint

b) multiple imputed dataset of participants with at least partially missing information on electronic primary care records in year 1 to 5 (PROC MI/PROC MIANALYZE)

c) threshold for 'continuous medication' changed to '≥ 2 prescriptions annually'

d) threshold for 'continuous medication' changed to '≥ 6 prescriptions annually'

e) threshold for 'continuous medication' changed to '≥ 12 prescriptions annually'
