# Supplementary material for: How good are GPs at adhering to a pragmatic trial protocol in primary care? Results from the ADDITION-Cambridge cluster-randomised pragmatic trial
Source: BMJ Open. 2018 Jun 14;8(6):e015295. doi: 10.1136/bmjopen-2016-015295 (PMC6009504; doi:10.1136/bmjopen-2016-015295)
Supplement: Author's manuscript [file bmjopen-2016-015295.draft_revisions.pdf]

How good are GPs at adhering to a pragmatic trial protocol in primary care? Results from the ADDITION-Cambridge cluster-randomized pragmatic trial

Journal:	*BMJ Open*
Manuscript ID	bmjopen-2016-015295
Article Type:	Research
Date Submitted by the Author:	28-Nov-2016

Complete List of Authors:	Laxy, Michael; Helmholtz Zentrum München, Institute of Health Economics and Health Care Management<br>Wilson, Edward; University of Cambridge, Cambridge Centre for Health Services Research<br>Boothby, Clare; Institute of Metabolic Science, MRC Epidemiology Unit<br>Griffin, Simon; Institute of Metabolic Science, MRC Epidemiology Unit

**Primary Subject Heading**:	Health services research
Secondary Subject Heading:	Diabetes and endocrinology
Keywords:	diabetes mellitus, pragmatic trial, protocol adherence, PRIMARY CARE

SCHOLARONE™ Manuscripts

For peer review only

For peer review only - http://bmjopen.bmj.com/site/about/guidelines.xhtml

How good are GPs at adhering to a pragmatic trial protocol in primary care? Results from the ADDITION-Cambridge cluster-randomized pragmatic trial

Authors: Michael Laxy[1,2,3], Edward C.F. Wilson[4], Clare E. Boothby[3], Simon J. Griffin[3]

[1] Institute of Health Economics, Helmholtz Zentrum München, Neuherberg, Germany, Ingolstädter Landstraße 1, 85764 München

[2] German Center for Diabetes Research, Neuherberg, Germany, Ingolstädter Landstraße 1, 85764 München

[3] MRC Epidemiology Unit, University of Cambridge, Cambridge, UK, Cambridge Biomedical Campus, Cambridge CB2 0SL

[4] Cambridge Centre for Health Services Research, University of Cambridge, Cambridge, UK, Forvie Site Cambridge Biomedical Campus, Cambridge CB2 0SR

Email addresses:

Michael Laxy: michael.laxy@helmholtz-muenchen.de

Ed Wilson: ew442@medschl.cam.ac.uk

Clare Boothby: Clare.Boothby@mrc-epid.cam.ac.uk

Simon Griffin: sjg49@medschl.cam.ac.uk

Corresponding author: Dr. Ed Wilson, Cambridge Centre for Health Services Research, University of Cambridge, Institute of Public Health, Forvie Site, Robinson Way, Cambridge CB2 0SR; Email: ew442@medschl.cam.ac.uk

Manuscript word count: 5,070

Key words: diabetes mellitus, pragmatic trial, protocol adherence, primary care

For peer review only - http://bmjopen.bmj.com/site/about/guidelines.xhtml

Abstract

Objective: To assess the fidelity of general practitioners' (GP) adherence to a long term pragmatic trial protocol.

Design: Analyses of electronic primary care records of participants in the pragmatic cluster-randomised ADDITION (Anglo-Danish-Dutch Study of Intensive Treatment In People with Screen Detected Diabetes in Primary Care)-Cambridge trial, comparing intensive multi-factorial treatment (IT) vs. routine care (RC).

Setting: Primary care surgeries in the East of England

Study sample/participants: A subsample (189 patients) of patients from the ADDITION-Cambridge cohort (867 patients), consisting of 40-69 year old patients with screen detected diabetes mellitus.

Interventions: In the RC-arm treatment was delivered according to concurrent treatment guidelines. Surgeries in the IT-arm received funding for additional contacts between GPs/nurses and patients, and GPs were advised to follow more intensive treatment algorithms for the management of glucose, lipids and blood pressure and aspirin therapy than in the RC-arm.

Outcome measures: The number of annual contacts between patients and GPs/nurses, the proportion of patients receiving prescriptions for cardio-metabolic medication in years 1 to 5 after diabetes diagnosis, and the adherence to prescription algorithms.

Results: The difference in the number of annual GP contacts (β=0.65) and nurse contacts (β=-0.15) between the study arms was small and insignificant. Patients in the IT-arm were more likely to receive glucose-lowering (OR=3.27), ACE-inhibiting (OR=2.03) and lipid-lowering drugs (OR=2.42, all p-values<0.01) than patients in the RC-arm. The prescription adherence varied between medication classes, but improved in both trial arms over the 5 year follow-up time.

Conclusions: The adherence of GPs to different aspects of the trial protocol was mixed. Background changes in health care policy need to be considered as they have the potential to dilute differences in

For peer review only - http://bmjopen.bmj.com/site/about/guidelines.xhtml

treatment intensity and hence incremental effect. Intensive prescribing of medication was well implemented, suggesting that positive effects on cardiovascular morbidity may be observed in the longer term.

Clinical trial number: ISRCTN86769081

Article Summary: Strengths and Limitations of the Study

- Pragmatic trials aim to produce externally valid results for decision makers. If and to what extent pragmatic trial interventions are delivered to patients often remains unknown.
- This study describes the adherence of GPs to the ADDITION trial protocol and hence provides a unique insight about what we can expect in future long-term pragmatic studies in the primary care context, particularly in the context of policy and guideline changes.
- Analyses are based on a subsample of participants of the ADDITION-Cambridge trial conducted in East England. Therefore, the generalizability of results might be limited.

For peer review only - http://bmjopen.bmj.com/site/about/guidelines.xhtml

Background

Type 2 Diabetes is an increasing public health problem associated with premature mortality and costly micro- and macro-vascular complications in terms of both reduced quality of life and financial burden, causing substantial economic pressure on healthcare systems and societies [1-4].

Previous research has shown that intensive treatment of cardiovascular risk factors is an effective and cost-effective intervention for patients with longstanding diabetes or routinely diagnosed diabetes [5-8]. In contrast, little was known about the cost-effectiveness of intensive primary care based treatment in patients in the early stages of the disease, such as screen detected populations. The pragmatic cluster-randomised ADDITION (**A**nglo-**D**anish-**D**utch Study of **I**ntensive **T**reatment **I**n People with Scree**n** Detected Diabetes in Primary Care) - trial (ISRCTN86769081) was one of the first studies addressing this important question [9-11]. Results showed that, compared to routine care, early intensive treatment modestly improved levels of cardiovascular risk factors, but did not significantly reduce the incidence of cardiovascular events, microvascular complications, and cardiovascular/overall mortality over the 5 year study period [12-14].

Pragmatic trials aiming to generate externally valid evidence in a real world setting, such as ADDITION, always present uncertainties concerning the implementation of the planned interventions in daily practice. Unlike highly controlled efficacy trials in which compliance to a (one-dimensional) intervention can (and must) be assured, the purpose of pragmatic trials is to assess the effectiveness of a (complex, multifactorial) intervention in routine settings. In the ADDITION-Cambridge trial, intensive treatment (IT) was compared to routine care (RC) for screen detected diabetes patients. IT in ADDITION was a multifactorial intervention including treatment targets and treatment algorithms that were more intensive than those in contemporary UK national treatment guidelines, as well as educational material for patients [10; 15-17]. However, the degree to which protocol components were implemented into practice, and hence the degree to which more intensified treatment was actually provided to patients in the intervention arm, has remained unknown. Furthermore, potential changes in national treatment guidelines towards more intensive care, and the introduction of the pay

For peer review only - http://bmjopen.bmj.com/site/about/guidelines.xhtml

For peer review only

for performance system in England within the national Quality and Outcomes Framework (QOF) [18; 19], are likely to have improved routine care and may have diluted the difference in treatment intensity between the study arms over time [20].

Beyond improving understanding of the results of the ADDITION-Cambridge study, knowledge about whether and how the intervention was actually delivered in practice can inform future pragmatic trials in relation to barriers to protocol adherence, and the difference in treatment intensity that can be expected in a primary care based pragmatic trial in the context of background policy changes.

The objective of this study was therefore to describe the adherence of GPs to the trial protocol and to compare the intensity of care delivered to screen detected diabetes patients between the trial arms.

Methods

Study design

The ADDITION-Cambridge study protocol has been published elsewhere[10]. In brief, ADDITION-Cambridge is part of the ADDITION-Europe trial, which consisted of two phases: a screening program and a pragmatic, cluster-randomised trial comparing the effect of early intensive treatment versus routine care on five year cardiovascular risk in patients with screen-detected type 2 diabetes mellitus [9]. The primary endpoint was a composite of cardiovascular morbidity and mortality (cardiovascular death, non-fatal myocardial infarction, non-fatal stroke, non-traumatic amputations and revascularisations).

Study population

For ADDITION-Cambridge, 33,539 eligible individuals were invited to stepwise screening. Individuals eligible for screening were people registered at one of the participating general practices around Cambridge, aged 40 to 69 years, not known to have diabetes and with a diabetes risk score of >0.17 (corresponding to the top 25% of the population distribution). The risk score included age, sex, BMI, steroid and antihypertensive medication as well as smoking and family history [21]. Exclusion

For peer review only - http://bmjopen.bmj.com/site/about/guidelines.xhtml

criteria were assessed by the potential participant's GP. Patients with severe illnesses with a life expectancy of less than 12 months, those with psychological or psychiatric disorders that might invalidate informed consent and those who were housebound, pregnant or breast feeding were excluded from the study. 867 eligible patients (from n=49 surgeries) with screen detected diabetes participated in the pragmatic primary care based intervention trial. Ethical approval was granted by the Eastern Multi-Regional Ethics Committee (ref 02/5/54). Written informed consent was obtained from all participants. This trial is registered as ISRCTN86769081.

The analyses reported in the present study are based on a subsample of the ADDITION-Cambridge trial population consisting of all patients with a primary endpoint in the 5 years of follow-up (n=63 patients) and two randomly selected patients from the same surgery without a primary endpoint (n=126 patients). In total, the subsample included 189 patients (RC: n=99 patients, IT: n=90 patients) from 34 surgeries. The study design is illustrated in detail in **figure 1**.

Intensive Treatment and Routine Care

Patients were treated according to the treatment allocation of their surgery. In the RC-arm patients received diabetes care through the National Health Service according to current UK guidelines and recommendations [15-17]. In the IT-arm additional features were added to current RC:

a) Surgeries received funding for 3 additional 10-minute GP consultations and 3 additional nurse consultations in the first 3 years after diagnosis.

b) Treatment algorithms were introduced along with underlying evidence demonstrating positive effects on CVD risk factors among patients with type 2 diabetes. In the IT-arm therapy with glucose lowering medication was indicated if $HbA_{1c} \geq 6.5\%$; ACE inhibitors/ARBs if BP ≥ 120/80mmHg; statins if cholesterol ≥ 3.5 mmol/l; and aspirin for all patients independent of their risk factor levels (assuming that patients had no contraindications). The thresholds for treatment initiation for glucose lowering, BP lowering and lipid lowering medication and for aspirin therapy in both the IT-arm (based on the trial protocol [10]) and the RC-arm (based on national guidelines [15-17]) are summarized in **Table 1**.

For peer review only - http://bmjopen.bmj.com/site/about/guidelines.xhtml

For peer review only

Table 1:
Criteria for the initiation of glucose lowering, blood pressure lowering, lipid lowering and platelet inhibiting (aspirin) medication according to the trial protocol (IT-arm) and national guidelines (RC-arm) ɫ

	Glucose-lowering therapy	Blood pressure-lowering therapy	Lipid-lowering therapy	CVD risk-lowering aspirin therapy
**Routine Care (RC)**	- if HbA_{1c} ≥ 7% ˫	- if BP ≥ 160/100<br>- if 140/80 mmHg ≤ BP < 160/100 mmHg and either prevalent CVD or 10-year CHD risk ≥ 15%<br>*(ACE inhibitors, ARBs, Β-blockers or diuretics as first choice)*	- if cholesterol ≥ 5 mmol/l or triglycerides ≥ 2.3mmol/l<br>- if prevalent CVD or 10-year CHD-risk ≥ 15%	- if prevalent CVD or 10-year CHD-risk ≥ 15%
**Intensive Treatment (IT)**	- if HbA_{1c} ≥ 6.5%	if ≥ 120/80 mmHg or prevalent CVD<br>*(ACE inhibitors/ARBs as first choice)*	- if cholesterol ≥ 3.5mmol/l	- independent of risk profile

ɫ Criteria are based on the national treatment guidelines from 2002 [15-17] and the ADDITION trial protocol [10]
˫ a range of 6.5% - 7.5% was mentioned. Consequently, the arithmetic mean of the borders (7%) was used as threshold

This figure does not claim to comprehensively describe the national treatment algorithms from the year 2002 or the detailed ADDITION trial protocol. It only highlights the differences in criteria for the initiation of drug therapy between IT and RC and does not account for possible contraindications.

c) Practice teams received theory-based educational materials to hand over to the patients, aiming to provide a shared framework for the management of their disease. Furthermore, GPs were advised to refer patients to a dietician and patients were encouraged through their GPs and nurses to increase their physical activity, to avoid excessive alcohol intake, to lose weight, to stop smoking, to adhere to medication, and to self-monitor blood glucose if given a glucometer by their GP.

Intensive treatment was promoted to participating surgeries by practice-based educational meetings with GPs and nurses. This included initial practice-based academic detailing conducted by a diabetologist and an academic GP to introduce treatment algorithms, and two interactive practice-based feedback sessions (approximately 6 and 14 months after the initial education session) to support and monitor treatment delivery.

Measures of treatment intensity

Information on the intensity of delivered care was extracted from the electronic primary care records of participating patients from the date of the diabetes diagnosis until December 2010 by a researcher unaware of the general practice study group allocation. These files recorded the date and type of delivered services, including consultations with primary care health professionals, prescribed medications and laboratory measurements/tests. For the analyzed trial population more than 80,000

For peer review only

For peer review only - http://bmjopen.bmj.com/site/about/guidelines.xhtml

'observations' were available in the first 5 years after diagnosis. Clear text functions were used and algorithms were derived to classify the obtained information. Ambiguous observations were screened and coded by hand. Anatomic Therapeutic Chemical (ATC) codes were assigned to drugs to categorize medication classes. The intensity of care indicators were defined as follows:

Contact with health care professionals: The annual number of contacts between patients and GPs (including GP partners, GP principals, GP associates, out of hours doctors) and nurses (including practice nurses, nurse practitioners and nurse specialists).

Medication: Continuous treatment (≥ 4 prescriptions annually) with glucose lowering drugs (metformin, sulphonylurea, thiazolidinedione, insulin, other glucose lowering drugs), ACE inhibiting drugs (ACE inhibitors or ARBs), lipid lowering drugs (statins, other cholesterol lowering drugs) or aspirin.

Monitoring of risk factor levels: Regular monitoring of glycaemic control (≥ 2 HbA_{1c} tests per year), lipid profile (≥ 1 cholesterol test per year) and kidney function (≥ 1 urine albumin-creatinine ratio (UACR) test per year) [15-17].

Statistical Analyses

We analysed the difference in treatment intensity within the first 5 years from date of diagnosis. The study period was subdivided into five annual intervals representing year 1 (day 1 – day 365) to year 5 (day 1460 – day 1825) from diagnosis. 16 patients whose electronic primary care records did not contain information for at least one entire year were excluded from the base-case intention to treat (ITT) analysis. The remaining 173 patients from 34 general practice surgeries with a mean cluster size of 5 patients (range: 2 to 17) were included in the analyses (IT: 82 patients from 18 surgeries, RC: 91 patients from 16 surgeries). Due to non-availability of data, surgery changes and deaths the total number of complete observed patient-years over the follow up period was 827 for contact with health care professionals and monitoring and 737 for prescriptions.

We applied linear regression models separately for years 1 to 5 in order to analyse the difference in the number of contacts with GPs and nurses for each individual year. A multi-level linear regression

For peer review only - http://bmjopen.bmj.com/site/about/guidelines.xhtml

model accounting for repeated observations (year 1-5) within patients was applied to test the overall difference in the number of annual contacts between the study arms over the 5 year study period. This model included an interaction term between the year since diagnosis and the treatment to capture any time – treatment interactions.

In parallel to the linear regression models for the frequency of contacts with health care professionals, logistic regression models were applied to assess the likelihood of receiving continuous medication (≥ 4 prescriptions annually). In a secondary analysis, we also examined the likelihood of receiving regular monitoring of glycaemic control, lipid profile and kidney function and the likelihood of seeing a dietician[15-17].

Linear and logistic regression models were adjusted for age and sex and accounted for patients being clustered into surgeries (2-level model for stratified analyses and 3-level models for overall analyses). As the non-random selection of the analysed subsample does not exactly represent the study population, we tested in a sensitivity analysis if the introduction of a weighting factor (inverse probability of being included in the study based on the status of having a primary endpoint) has an impact on the results. We also altered the thresholds for the definition of 'continuous' medication (from 4 to 2, 6 and 12 prescriptions) to assess the sensitivity towards these threshold definitions. To assess the sensitivity to missing data we further refitted the analyses to a regression-based multiply-imputed (n=10 imputations) dataset (n=189 patients). Statistical analyses were performed with SAS 9.3 using the GLIMMIX, MI and MIANALYZE procedure (Cary, NC).

To gain a more detailed insight into the pattern of GPs' adherence to treatment algorithms, we further extracted clinical information including HbA_{1c}, BP, cholesterol, triglycerides, prevalent CVD (defined as MI or stroke) and 10-year modelled CHD risk (using the UKPDS risk engine V2) from the baseline, year 1 and year 5 examinations of the ADDITION study. Missing clinical values were imputed by the methods of 'last observation carried forward' and 'first observation carried backwards' to avoid shrinkage of the sample size. We calculated the proportion of patients who should have received medication, i.e. the proportion of patients whose clinical values exceeded the

For peer review only - http://bmjopen.bmj.com/site/about/guidelines.xhtml

thresholds referred to in the trial protocol [10] and the national guidelines[15-17] (P [clinical value ≥ threshold]) and the proportion of patients who actually received at least one prescription in a time frame of 3 months after the date of the laboratory measurement (P [# of prescriptions ≥ 1]) (Table 1). We finally defined the adherence of GPs to the trial protocol/national guidelines descriptively as the proportion of patients who receive at least one prescription, out of those patients whose clinical values exceed the thresholds (P [# of prescriptions ≥ 1] | [clinical value ≥ threshold]).

Results

Baseline sample characteristics

Characteristics of the sample at baseline are shown in **Table 2**. The mean age of the sample was 62 years, 34% were female and 96% Caucasian. The biomedical characteristics of the comparison arms were balanced. No differences were observed between the full sample (n=189) and the analysis sample (n=173).

Table 2:
Baseline characteristics of the used subsample of ADDITION Cambridge

	Intensive Treatment	Routine Care
N	82	91
Number of primary outcomes n (%)	27 (33.3)	33 (33.3)
Female sex, n (%)	30 (36.6)	30 (30.3)
Caucasian ethnicity, n (%)	77 (93.9)	96 (97)
Age, mean (SD)	61.87 (7.28)	62.01 (6.81)
BMI [kg/m2], mean (SD)	33.6 (5.6)	33.8 (5.9)
Total cholesterol [mmol/L], mean (SD)	5.47 (1.12)	5.46 (1.22)
HDL [mmol/L], mean (SD)	1.16 (0.32)	1.2 (0.31)
Systolic blood pressure [mm Hg], mean (SD)	143 (20.8)	143.8 (22.2)
HbA_{1c} [%], mean (SD)	7.84 (2.09)	7.27 (1.59)

SD: Standard Deviation, BMI: Body Mass Index, HDL: High Density Lipoprotein, HbA_{1c}: glycated haemoglobin; N: number of individuals included in the analysis sample

Contact with health care professionals

The adjusted mean number of annual GP and nurse contacts is graphically illustrated in **Figure 2**. We found no difference in the mean annual number of contacts with GPs (IT: 5.80, vs. RC: 5.18, β=0.65 [95%-CI: -0.24, +0.13] or nurses (IT: 5.34 vs. RC: 5.49, β = -0.15 [-1.77, +1.48]) and no consistent trend over time.

For peer review only - http://bmjopen.bmj.com/site/about/guidelines.xhtml

Medication

The proportion of GPs who regularly prescribed (≥ 4 times annually) glucose lowering and cardio-protective drugs and odds ratios for the likelihood of regular prescriptions are shown in **Figure 3**.

GPs in the IT-arm were 3.27 [95%CI: 1.81 to 5.93] times more likely to regularly prescribe glucose lowering medications compared to GPs in the RC-arm, however, this difference diminished over the follow-up period as more patients in the RC arm were also prescribed medication. Patients in the IT-arm also had a greater chance of being prescribed lipid lowering medication (OR=2.42 [1.30 to 4.51]) and ACE inhibiting drugs (OR=2.02 [1.13, 3.65]), which were, in contrast to routine care guidelines, the first choice BP lowering drug according to the trial protocol. But no significant difference was observed between the trial arms for the category of BP lowering drugs as a whole (including beta-blocker, diuretics etc.) (OR=1.41 [0.71, 2.80]) (**Appendix 1**). No significant difference was observed between the trial arms for prescription of aspirin. Overall in both treatment arms, the likelihood of patients receiving glucose lowering, ACE inhibiting and lipid-lowering medications increased from diagnosis to five year follow up.

Monitoring of risk factors

The proportion of patients receiving regular HbA_{1c} tests (≥ 2 annually, 45% of patients), lipid tests (≥ 1 annually, 55% of patients) and UACR tests (≥ 1 annually, 75% of patients) was low. No significant difference was observed between the treatment arms (HbA_{1c} tests: OR=1.56 [0.63, 3.83], lipid tests OR=1.53 [0.51, 4.60], UACR-test: OR=0.82 [0.34, 1.98]) (**Appendix 1**).

Sensitivity Analysis

Analyses of multiple imputed data-sets led to qualitatively and quantitatively similar results. Also the introduction of a weighting factor to account for non-random patient selection yielded comparable results. Using different thresholds for the definition of 'continuous medication' showed that the results for glucose and lipid lowering medications were not sensitive to threshold definitions. However, increasing the threshold number for lipid lowering drugs attenuated the respective OR considerably (**appendix 2**).

For peer review only - http://bmjopen.bmj.com/site/about/guidelines.xhtml

For peer review only

Adherence to prescription algorithms

The proportions of patients who should have received medication according to national guidelines and the ADDITION trial protocol and the proportions of patients who actually received a prescription within 3 months following the assessment of bio-medical data are presented in *column 1 and column 2* of **Figure 4**: The black part in *column 2* represents the proportion of patients who received a prescription and whose clinical values exceeded the thresholds for medication prescription and the framed white part represents the proportion of patients who received medication although clinical values did not exceed the thresholds. Adherence to the prescription algorithms, i.e. the proportion of patients who received at least one prescription out of those patients whose clinical values exceeded the thresholds (P [# of prescriptions ≥ 1] | [clinical value ≥ threshold]) is shown numerically in the lower part of **Figure 4**.

Due to tighter algorithms in the trial protocol (IT-arm) than in the national guidelines (RC-arm) more patients in the IT-arm were eligible for glucose-lowering, BP lowering and aspirin therapy than in the RC-arm. However, despite lower cholesterol thresholds in the IT-arm compared to the RC-arm, treatment with lipid lowering medication was indicated in almost equal proportions of patients in the two treatment arms.

Glucose lowering drugs: In the first year, the adherence to the treatment algorithm was generally low, but considerably higher in the IT-arm than in the RC-arm. At year 5, 73% of patients in both treatment arms with an HbA_{1c} ≥ threshold-level received a prescription.

BP- lowering/ACE inhibiting drugs: In the IT arm, adherence to the guideline for prescription of ACE inhibiting medication increased from 41% at baseline to 77% at year 5. In the RC arm, guideline adherence for prescription of any BP lowering medication increased from 55% at baseline to 94% at year 5 and 'prescription adherence' to ACE inhibiting medication (ACE inhibitors were not mentioned in the guidelines to be the first line treatment in RC) increased from 28% at baseline to 64% at year 5 (not shown).

For peer review only - http://bmjopen.bmj.com/site/about/guidelines.xhtml

Lipid lowering drugs: Adherence to the treatment algorithms increased in both treatment arms and was consistently better in the IT-arm. At year 5, most patients with clinical values greater than threshold-levels were treated (IT-arm 93%, RC-arm 81%).

Aspirin: The adherence to the trial protocol/guidelines was low, less than 50% of eligible patients in both treatment arms received aspirin.

Discussion

Summary

ADDITION is a large pragmatic primary care based trial aiming to promote intensive multifactorial treatment of patients with screen detected diabetes by GPs. Utilizing electronic primary care records of patients, this study shows that GPs in the IT-arm did not see their patients more often, but were more likely to regularly prescribe metabolic and cardio-protective drugs. Generally, GPs' adherence to prescription algorithms increased substantially in both trial arms over the 5 year follow-up period. Large time-treatment interactions for prescription of glucose lowering medication indicates that background changes in routine care might have diluted the difference in treatment intensity over time.

Contextual frame

Pragmatic ("effectiveness") trials seek to produce externally valid results in order to inform the process of decision-making by policy makers [22-25]. However, unlike in explanatory ("efficacy") trials, adherence to protocol is rarely tightly monitored and the degree to which the intervention is implemented often remains uncertain. In the case of non-statistically significant results, this begs the question whether the intervention is *per se* not efficacious in the tested (heterogeneous) population, or whether the intended difference in treatment intensity was not big enough to detect any effects in the given sample size.

Lack of a difference in the intensity of treatment can be due to different reasons. Firstly, adherence of responsible health care professionals to the protocol might be low due to limited motivation, insufficient monetary resources or lack of interest in the ongoing trial. To tackle this issue, in

For peer review only - http://bmjopen.bmj.com/site/about/guidelines.xhtml

ADDITION-Cambridge, a detailed trial protocol was specified and the implementation of the protocol elements was incentivized by additional monetary resources and supported by an initial practice-based academic and two interactive feedback sessions[10]. Secondly, daily treatment delivered in practice might differ from both guidelines and what happens in practices participating in research. Not considering actual practice in routine care can result in intervention plans that fail to induce treatment differences between the trial arms. The choice of suitable interventions is therefore particularly challenging in multi-national trials like ADDITION, where guidelines or daily practice in countries might differ but a certain degree of intervention homogeneity is warranted[9]. Thirdly, policy changes, such as changes in the remuneration system and modifications in treatment guidelines, can intensify routine care, thus potentially diluting differences between the intervention and routine care arm. Long-term trials such as ADDITION are particularly susceptible to such influences. Between 2003 (~start of the study) and 2008/09 (~end of the 5 year analysis period) in the UK no new national diabetes treatment guidelines were released. However, in 2004 the Quality and Outcomes Framework (QOF) with its pay for performance system was launched [18] and extended in the following years. The QOF incentivised fulfilment of basic quality of care indicators by monetary resources and may have improved the quality of care for patients with various conditions, including diabetes [20; 26].

Principal findings

Our study shows that although surgeries in the IT-arm received monetary resources for additional consultations, GPs and nurses did not see their patients more often, nor were they more likely to perform regular HbA_{1c}, lipid or UACR tests. This result might be explained by the fact that the patients in the RC-arm already saw their GP/nurse on average 5-6 times a year, which is more than the average ~4 GP and ~2.5 nurse contacts per year for the general UK population [27]. Therefore the GPs (and indeed the patients) may have felt that this was sufficient to adequately monitor the condition. It also shows that monetary incentives might help to convince a reasonable number of surgeries to participate in long-term extensive trials such as ADDITION (46% of contacted surgeries agreed to join the study), but that financial incentives might not be successful in motivating GPs to further increase treatment intensity if it is already at a high level [10]. In contrast, our results indicate

For peer review only - http://bmjopen.bmj.com/site/about/guidelines.xhtml

that the education sessions and feedback audits had a positive impact on the protocol adherence of GPs, as in general adherence to the treatment algorithms in the IT-arm was higher than adherence to the national guidelines in the RC-arm. This finding supports previous research that feedback loops can help to maximize guideline adherence in primary care [28; 29].

According to the clinical thresholds outlined in the trial protocol and the national guidelines, more patients in the IT-arm than in the RC-arm were eligible to receive glucose-lowering, BP-lowering and platelet-inhibiting drugs (**figure 4**). This suggests that the ADDITION intervention was designed at an appropriate level for the context, as even with a hypothetical prescription adherence of 100% patients in the IT-arm should have received more intensive treatment than patients in the RC-arm.

Notably, a very high proportion of patients in the RC-arm already received BP-lowering medication at baseline, many of them although there BP levels did not exceed thresholds. The finding of high BP-lowering prescription prevalence probably results from the fact that treatment with BP lowering medication was part of the risk-score used to identify high risk individuals eligible for diabetes screening in the first phase of the ADDITION trial [10]. There could be two reasons why many of the patients who received BP-lowering prescriptions had no clinical indication for treatment. On the one hand, these patients might have previously had uncontrolled BP levels, but treatment with BP lowering medication brought their BP under control. On the other hand, it is possible that the daily practice for BP control at this time was already much stricter than recommended by the guidelines. Independently of its origin, the initially high prevalence of BP-lowering medication in both trial arms might be the reason why we did not observe a difference in the proportion of patients prescribed BP lowering drugs. Consequently, the observed difference in ACE inhibiting drugs may be due to GPs switching from diuretics or beta-blockers to ACE inhibiting drugs, as recommended by the trial protocol.

The low adherence to recommendations concerning aspirin therapy observed in both trial arms is interesting, as this prescription behaviour could be interpreted as a general scepticism among GPs (and perhaps patients) towards the weak evidence of benefits of aspirin therapy for primary

For peer review only - http://bmjopen.bmj.com/site/about/guidelines.xhtml

prevention of cardiovascular disease [6]. The results of subsequent large trials justify such scepticism [30; 31]. Alternatively, some patients may have obtained aspirin from the pharmacy without a prescription without this being noted in the electronic medical record.

Except for aspirin, adherence to prescription algorithms increased substantially over the follow-up period. We assume that this finding is triggered by the progression and duration of the disease and by general improvements in the overall quality of care over time, independently of disease progression [32]. The significant interaction between ‘treatment’ and ‘time since diagnosis’ for glucose lowering medication indicates changing treatment patterns in the RC-arm which might be triggered by policy changes, like QOF. However, due to methodological limitations (covariate co-linearity, power problems in stratified models) this question could not be adequately addressed with the available data.

Implications for the planning of future pragmatic trials

This study shows that the successful implementation of a pragmatic trial in primary care is possible, but there are issues that need to be considered. Namely, (1) a high standard of care in control practices questions the need for further intensification, (2) treatment of patients in the RC-arm that did not reflect the national guidelines, and (3) background policy changes affecting quality of routine care. These issues need to be identified, considered and addressed when designing a pragmatic study or rolling out an intervention comprehensively [23; 24; 33]. The results further underline the potential importance of standard good practice in (pragmatic) trials. Methods such as initial academic detailing and repeated feedback sessions may be of great importance for the overall success of the study [24; 34]. In this context, more qualitative or quantitative implementation research may help to identify and test strategies that affect the adherence of health care professionals (and patients) [35].

Ideally, pragmatic trials of complex interventions should, if possible, be designed in a way that allows evaluation of the adherence of health care professionals to the trial protocol and of patients to the chosen treatment regimen. This study shows that the use of electronic primary care records is a promising approach for assessing the adherence of GPs. The obtained data is also useful for health economic research. In this particular example, the new primary care data can be used to update a

For peer review only - http://bmjopen.bmj.com/site/about/guidelines.xhtml

previous analysis to reduce uncertainty on the cost-effectiveness of the intervention [36], a method consistent with an iterative approach to research and decision making [37-39].

Implications for the interpretation of trial results

Intensified prescription algorithms were well implemented into practice. We found that prescription with glucose lowering, ACE inhibiting and lipid lowering drugs was higher in the IT-arm. The expected treatment effect resulting from this difference in medication could be interpreted as an area under the curve issue: The combination of the magnitude and the duration of the treatment difference can be expected to be the crucial driver of long-term effects. The extended follow-up of the UKPDS trial, which aimed to reduce diabetes related complications through tighter glucose and BP control, has shown that after the termination of the intervention, between-group differences in laboratory measurements disappeared [40-43]. However, the reductions in risk of micro and macro-vascular complications persisted (or increased) for patients who had received tight glucose control, but not for patients who had received tight BP control [40; 41]. In ADDITION we observed a small but significant improvement in HbA_{1c}, BP and cholesterol levels in the IT-arm and a non-significant reduction in risk of the composite CVD endpoint (RR=0.83, p=0.12) over a 5 year time period [14]. This study shows that the proportion of patients receiving glucose-lowering drugs in each arm had equalised at the end of the 5 year observation period, suggesting that the differences in glycaemic control might disappear in the subsequent years. However, as a substantially greater proportion of patients in the IT-arm received ACE inhibiting and lipid lowering drugs, it can be assumed that differences in BP and lipids might be sustained. If between-group differences in treatment for blood pressure and lipids diminish so will the levels of risk factors, however the CVD risk may remain lower due to potential legacy effects of earlier reductions in glucose and cholesterol. Given that the number of events will also increase over time, it may be that the ADDITION intervention will appear effective in the long-term. The ten year follow up of ADDITION will quantify the long term effect of relatively small differences in treatment and risk factors observed in the first 5 years after diagnosis of diabetes by screening [14].

For peer review only - http://bmjopen.bmj.com/site/about/guidelines.xhtml

Strengths and limitations

To our knowledge, this is one of the first studies to comprehensively analyse the adherence of GPs to a pragmatic trial protocol in primary care. In contrast to self-reported information from patients, electronically stored primary care records provide a high degree of detail about all GP-based primary care services delivered to patients and are less susceptible to recall bias [44]. Through the linkage of clinical information from the trial measurements with information on prescriptions from the electronic primary care records, it was further possible to comprehensively describe and analyse the prescription adherence of GPs to the trial protocol and national guidelines.

However, we only had data from a subsample of the ADDITION-Cambridge trial-cohort with an oversampling of patients with a primary event during the follow-up period. As our weighted sensitivity analyses showed that this issue did not affect the results, the findings of this study are likely to be generalizable to the sample of practices who participated in the ADDITION trial. Nevertheless, the generalizability of results to average GP practices in the UK might be quite limited. The practices that take part in research tend to be more organised and deliver better quality routine care than those declining to participate. This might lead to ceiling effects for interventions, i.e. it appears to be hard to induce a difference in treatment intensity between RC and a more intensive treatment regimen. Another limitation is that in our assessment of prescription adherence, we did not take into account possible contra-indications for medications as well as patients'views, and analysed the data from a rather non-situational, disease-orientated perspective [45; 46]. Shared decision making between the GP and the patient might reasonably lead to decisions that deviate from those in the protocol (and national guidelines). We therefore do not know if patients or GPs were the main determinants of protocol non-adherence. It is possible that patients did not agree to start medication or to come to the surgery more often. To completely understand the adoption of the intervention the patient's role also needs to be taken into account, which was impossible with the chosen approach. Finally, although the accuracy of primary care records for GP-based services is known to be quite high, particularly for prescribed medication and laboratory tests, the handling, merging and extraction of free text data from numerous observations (~80,000) originating from different IT format systems

For peer review only - http://bmjopen.bmj.com/site/about/guidelines.xhtml

is challenging and validation was not undertaken [44]. Consequently, it is possible that a small proportion of services might be misclassified, resulting in non-differential bias.

Conclusion

This study demonstrates that the successful implementation of long-term pragmatic trials in primary care is possible, but there are many obstacles especially during periods of significant change in routine care. The retrospective analyses of the electronic primary care records of participants in the ADDITION-Cambridge trial shows that intensive treatment was fairly well implemented into practice, suggesting that positive effects on cardiovascular morbidity and mortality might be expected in the long-term. Where possible, data needed to evaluate the fidelity of stakeholders to trial protocols should be collected routinely in future pragmatic trials as this information is invaluable for the interpretation of study results and for the planning of future studies.

Data Sharing Statement

The access policy for sharing is based on the MRC Policy and Guidance on Sharing of Research Data from Population and Patient Studies. All data sharing must meet the terms of existing participants' consent and study ethical approvals.

Information on data and data requests can be found on http://epi-meta.medschl.cam.ac.uk/includes/addcam/addcam.html. In case of questions please contact datasharing@mrc-epid.

For peer review only - http://bmjopen.bmj.com/site/about/guidelines.xhtml

Author contribution

ML, EW, CB and SG designed the concept for the paper. ML performed the statistical analysis, interpreted the data and drafted the manuscript. SG and CB were involved in collecting the data. EW provided statistical support. All authors critically revised the intellectual content of the manuscript and approved its final version.

Conflict of interest statement

None of the authors has competing interests.

Acknowledgements

We gratefully acknowledge the contribution of all participants, practice nurses and general practitioners in the ADDITION-Cambridge study (a full list of participating practices is given below) and Kit Coutts and Dr Rebecca Simmons for their contribution to data collection. We also acknowledge the contribution of the trial steering committee (Professors Nigel Stott (Chair), John Weinman, Richard Himsworth, and Paul Little) and Prof Jane Armitage and Dr Louise Bowman who adjudicated the endpoints.

Funding

ADDITION-Cambridge was supported by the Wellcome Trust (grant reference No G061895) the Medical Research Council (grant reference no: G0001164), National Health Service R&D support funding (including the Primary Care Research and Diabetes Research Networks), and the National Institute for Health Research. We received an unrestricted grant from University of Aarhus, Denmark, to support the ADDITION-Cambridge trial. Bio-Rad provided equipment to undertake capillary glucose screening by HbA1c in general practice. SG is a National Institute for Health Research (NIHR) Senior Investigator. The Primary Care Unit is supported by NIHR Research funds. SJG received support from the Department of Health NIHR Programme Grant funding scheme (RP-PG-0606-1259). The views expressed in this publication are those of the authors and not necessarily those of the NHS, the NIHR, or the Department of Health.The Primary Care Unit and the Medical Research Council Epidemiology Unit at the University of Cambridge jointly coordinated the study. Aside from the authors, the ADDITION-Cambridge study team has included Rebecca Abbott, Amanda Adler, Judith Argles, Gisela Baker, Rebecca Bale, Ros Barling, Daniel Barnes, Mark Betts,

For peer review only

Sue Boase, Sandra Bovan, Gwen Brierley, Ryan Butler, James Brimbicombe, Parinya Chamnan, Sean Dinneen, Pesheya Doubleday, Justin Basile Echouffo-Tcheugui, Sue Emms, Mark Evans, Tom Fanshawe, Francis Finucane, Philippa Gash, Julie Grant, Wendy Hardeman, Robert Henderson, Susie Hennings, Muriel Hood, Garry King, Ann-Louise Kinmonth, Georgina Lewis, Christine May Hall, Joanna Mitchell, Richard Parker, Nicola Popplewell, A Toby Prevost, Emanuella De Lucia Rolfe, Richard Salisbury, Lincoln Sargeant, Rebecca Simmons, Stephen Sharp, Megan Smith, Stephen Sutton, Nicholas Wareham, Liz White, Fiona Whittle and Kate Williams. We also wish to thank the Cambridge University Hospitals NHS Foundation Trust Department of Clinical Biochemistry and the NIHR Cambridge Biomedical Research Centre Core Biochemistry Assay Laboratory for carrying out the biochemical assays and the following groups within the MRC Epidemiology Unit: data management (Adam Dickinson), information technology (Iain Morrison, Rich Hutchinson), technical (Matt Sims), and field epidemiology (Paul Roberts, Kim Mwanza, James Sylvester, Gwen Brierley, Jaimie Taylor). ADDITION-Cambridge practices: Acorn Community Health Centre, Arbury Road Surgery, Ashwell Surgery, Birchwood Surgery, Bridge Street Medical Centre, Brookfields & Cherry Hinton, Broomfields, Buckden Surgery, Burwell Surgery, Cambridge Surgery, Cedar House Surgery, Charles Hicks Centre, Chequers Lane Surgery, Clarkson Surgery, Cornerstone Practice, Cornford House Surgery, Cottenham Surgery, Cromwell Place Surgery, Dr Smith and Partner (Cambridge), East Field Surgery, Ely Surgery, Freshwell Health Centre, George Clare Surgery, Great Staughton Surgery, Harston Surgery, Health Centre (Eaton Socon), Hilton House, John Tasker House, Lensfield Medical Practice, Manea Surgery, Mercheford House, Milton Surgery, Nene Valley Medical Practice, Nevells Road Surgery, New Roysia Surgery, Northcote House Surgery, Nuffield Road Medical Centre, Orchard Surgery, Orchard House Surgery, Orton Medical Practice, Park Medical Centre, Paston Health Centre, Peterborough Surgery, Petersfield Medical Practice, Prior's Field Surgery, Queen Edith's Medical Practice, Queen Street Surgery, Rainbow Surgery, Ramsey Health Centre, Riverside Practice, Roman Gate Surgery, Rosalind Franklin House, South Street Surgery, Thaxted Surgery, The Health Centre (Bury St Edmunds), The Old Exchange, The Surgery Stanground, Townley Close Health Centre, Trumpington Street Medical Practice, Werrington Health Centre, York Street Medical Practice.

For peer review only - http://bmjopen.bmj.com/site/about/guidelines.xhtml

References

1. Morgan CL, Currie CJ, Peters JR: Relationship between diabetes and mortality: a population study using record linkage. Diabetes Care 2000;23:1103-1107
2. Narayan KM, Gregg EW, Fagot-Campagna A, Engelgau MM, Vinicor F: Diabetes--a common, growing, serious, costly, and potentially preventable public health problem. Diabetes Res Clin Pract 2000;50:S77-84
3. Fowler MJ: Microvascular and Macrovascular Complications of Diabetes. Clinical Diabetes 2008;26:77-82
4. Schunk M, Reitmeir P, Schipf S, Volzke H, Meisinger C, Thorand B, Kluttig A, Greiser KH, Berger K, Muller G, Ellert U, Neuhauser H, Tamayo T, Rathmann W, Holle R: Health-related quality of life in subjects with and without Type 2 diabetes: pooled analysis of five population-based surveys in Germany. Diabet Med 2012;29:646-653
5. Gaede P, Lund-Andersen H, Parving HH, Pedersen O: Effect of a multifactorial intervention on mortality in type 2 diabetes. N Engl J Med 2008;358:580-591
6. Gaede P, Vedel P, Larsen N, Jensen GV, Parving HH, Pedersen O: Multifactorial intervention and cardiovascular disease in patients with type 2 diabetes. N Engl J Med 2003;348:383-393
7. Clarke PM, Gray AM, Briggs A, Stevens RJ, Matthews DR, Holman RR: Cost-utility analyses of intensive blood glucose and tight blood pressure control in type 2 diabetes (UKPDS 72). Diabetologia 2005;48:868-877
8. Gray A, Raikou M, McGuire A, Fenn P, Stevens R, Cull C, Stratton I, Adler A, Holman R, Turner R: Cost effectiveness of an intensive blood glucose control policy in patients with type 2 diabetes: economic analysis alongside randomised controlled trial (UKPDS 41). BMJ 2000;320:1373-1378
9. Lauritzen T, Griffin S, Borch-Johnsen K, Wareham NJ, Wolffenbuttel BH, Rutten G: The ADDITION study: proposed trial of the cost-effectiveness of an intensive multifactorial intervention on morbidity and mortality among people with Type 2 diabetes detected by screening. Int J Obes Relat Metab Disord 2000;24:S6-11
10. Echouffo-Tcheugui JB, Simmons RK, Williams KM, Barling RS, Prevost AT, Kinmonth AL, Wareham NJ, Griffin SJ: The ADDITION-Cambridge trial protocol: a cluster -- randomised controlled trial of screening for type 2 diabetes and intensive treatment for screen-detected patients. BMC Public Health 2009;9:1471-2458
11. Li R, Zhang P, Barker LE, Chowdhury FM, Zhang X: Cost-Effectiveness of Interventions to Prevent and Control Diabetes Mellitus: A Systematic Review. Diabetes Care 2010;33:1872-1894
12. Sandbaek A, Griffin SJ, Sharp SJ, Simmons RK, Borch-Johnsen K, Rutten GE, van den Donk M, Wareham NJ, Lauritzen T, Davies MJ, Khunti K: Effect of early multifactorial therapy compared with routine care on microvascular outcomes at 5 years in people with screen-detected diabetes: a randomized controlled trial: the ADDITION-Europe Study. Diabetes Care 2014;37:2015-2023
13. Simmons RK, Sharp SJ, Sandbaek A, Borch-Johnsen K, Davies MJ, Khunti K, Lauritzen T, Rutten GE, van den Donk M, Wareham NJ, Griffin SJ: Does early intensive multifactorial treatment reduce total cardiovascular burden in individuals with screen-detected diabetes? Findings from the ADDITION-Europe cluster-randomized trial. Diabet Med 2012;29:1464-5491
14. Griffin SJ, Borch-Johnsen K, Davies MJ, Khunti K, Rutten GE, Sandbaek A, Sharp SJ, Simmons RK, van den Donk M, Wareham NJ, Lauritzen T: Effect of early intensive multifactorial therapy on 5-year cardiovascular outcomes in individuals with type 2 diabetes detected by screening (ADDITION-Europe): a cluster-randomised trial. Lancet 2011;378:156-167

For peer review only - http://bmjopen.bmj.com/site/about/guidelines.xhtml

15. McIntosh AHA, Home PD, Brown F, Bruce A, Damerell A, Davis R, et al. Clinical guidelines and evidence review for Type 2 diabetes: management of blood glucose. University of Sheffield, 2001.
16. McIntosh AHA, Home PD, Brown F, Bruce A, Damerell A, Davis R, et al. Clinical guidelines and evidence review for Type 2 diabetes: management of blood pressure. University of Sheffield, 2002.
17. McIntosh AHA, Home PD, Brown F, Bruce A, Damerell A, Davis R, et al. Clinical guidelines and evidence review for Type 2 diabetes: Lipids management. University of Sheffield, 2002.
18. Health and Social Care Information Centre. National Quality and Outcomes Framework Statistics for England - 2004-05. retrieved from http://www.hscic.gov.uk/catalogue/PUB01946. last accessed: 05.02.2015.
19. Health and Social Care Information Centre. Quality and Outcomes Framework - 2006-07. retrieved from http://www.hscic.gov.uk/catalogue/PUB05997. last accessed: 05.02.2015.
20. Campbell S, Reeves D, Kontopantelis E, Middleton E, Sibbald B, Roland M: Quality of Primary Care in England with the Introduction of Pay for Performance. New England Journal of Medicine 2007;357:181-190
21. Clarke PM, Gray AM, Briggs A, Farmer AJ, Fenn P, Stevens RJ, Matthews DR, Stratton IM, Holman RR: A model to estimate the lifetime health outcomes of patients with type 2 diabetes: the United Kingdom Prospective Diabetes Study (UKPDS) Outcomes Model (UKPDS no. 68). Diabetologia 2004;47:1747-1759
22. Singal AG, Higgins PDR, Waljee AK: A Primer on Effectiveness and Efficacy Trials. Clin Trans Gastroenterol 2014;5:e45
23. Patsopoulos NA: A pragmatic view on pragmatic trials. Dialogues in Clinical Neuroscience 2011;13:217-224
24. Eldridge S: Pragmatic trials in primary health care: what, when and how? Family Practice 2010;27:591-592
25. Roland M, Torgerson DJ: What are pragmatic trials? BMJ 1998;316:285
26. Khunti K, Gadsby R, Millett C, Majeed A, Davies M: Quality of diabetes care in the UK: comparison of published quality-of-care reports with results of the Quality and Outcomes Framework for Diabetes. Diabetic Medicine 2007;24:1436-1441
27. Redekop WK, Koopmanschap MA, Stolk RP, Rutten GE, Wolffenbuttel BH, Niessen LW: Health-related quality of life and treatment satisfaction in Dutch patients with type 2 diabetes. Diabetes Care 2002;25:458-463
28. Ivers N, Jamtvedt G, Flottorp S, Young JM, Odgaard-Jensen J, French SD, O'Brien MA, Johansen M, Grimshaw J, Oxman AD: Audit and feedback: effects on professional practice and healthcare outcomes. Cochrane Database Syst Rev 2012;13
29. Goff DC, Jr., Massing MW, Bertoni AG, Davis J, Ambrosius WT, McArdle J, Duren-Winfield V, Sueta CA, Croft JB: Enhancing quality of heart failure care in managed Medicare and Medicaid in North Carolina: results of the North Carolina Achieving Cardiac Excellence (NC ACE) Project. Am Heart J 2005;150:717-724
30. Belch J, MacCuish A, Campbell I, Cobbe S, Taylor R, Prescott R, Lee R, Bancroft J, MacEwan S, Shepherd J, Macfarlane P, Morris A, Jung R, Kelly C, Connacher A, Peden N, Jamieson A, Matthews D, Leese G, McKnight J, O'Brien I, Semple C, Petrie J, Gordon D, Pringle S, MacWalter R, Prevention of Progression of Arterial D, Diabetes Study Group DRG, Royal College of Physicians E: The prevention of progression of arterial disease and diabetes (POPADAD) trial: factorial randomised placebo controlled trial of aspirin and antioxidants in patients with diabetes and asymptomatic peripheral arterial disease. BMJ : British Medical Journal 2008;337:a1840
31. Ogawa H, Nakayama M, Morimoto T, et al.: Low-dose aspirin for primary prevention of atherosclerotic events in patients with type 2 diabetes: A randomized controlled trial. JAMA 2008;300:2134-2141

For peer review only - http://bmjopen.bmj.com/site/about/guidelines.xhtml

32. Filion KB, Joseph L, Boivin JF, Suissa S, Brophy JM: Trends in the prescription of anti-diabetic medications in the United Kingdom: a population-based analysis. Pharmacoepidemiol Drug Saf 2009;18:973-976
33. Glasgow RE: What Does It Mean to Be Pragmatic? Pragmatic Methods, Measures, and Models to Facilitate Research Translation. Health Education & Behavior 2013;40:257-265
34. Godwin M, Ruhland L, Casson I, MacDonald S, Delva D, Birtwhistle R, Lam M, Seguin R: Pragmatic controlled clinical trials in primary care: the struggle between external and internal validity. BMC Med Res Methodol 2003;3:28
35. Peters DH, Adam T, Alonge O, Agyepong IA, Tran N: Implementation research: what it is and how to do it. BMJ 2013;347:f6753
36. Tao L, Wilson EC, Wareham NJ, Sandbaek A, Rutten GE, Lauritzen T, Khunti K, Davies MJ, Borch-Johnsen K, Griffin SJ, Simmons RK: Cost-effectiveness of intensive multifactorial treatment compared with routine care for individuals with screen-detected Type 2 diabetes: analysis of the ADDITION-UK cluster-randomized controlled trial. Diabet Med 2015;6:12711
37. Wilson E, Abrams K: From Evidence Based Economics to Economics Based Evidence: Using Systematic Review to inform the design of future research. In *Evidence Based Economics* Shemilt I, Mugford M, Vale L, Marsh K, Donaldson C, Eds. London, Blackwell Publishing, 2010
38. Claxton K, Cohen JT, Neumann PJ: When is evidence sufficient? Health Aff (Millwood) 2005;24:93-101
39. Sculpher M, Drummond M, Buxton M: The iterative use of economic evaluation as part of the process of health technology assessment. J Health Serv Res Policy 1997;2:26-30
40. Holman RR, Paul SK, Bethel MA, Matthews DR, Neil HAW: 10-Year Follow-up of Intensive Glucose Control in Type 2 Diabetes. New England Journal of Medicine 2008;359:1577-1589
41. Holman RR, Paul SK, Bethel MA, Neil HAW, Matthews DR: Long-Term Follow-up after Tight Control of Blood Pressure in Type 2 Diabetes. New England Journal of Medicine 2008;359:1565-1576
42. Tight blood pressure control and risk of macrovascular and microvascular complications in type 2 diabetes: UKPDS 38. UK Prospective Diabetes Study Group. Bmj 1998;317:703-713
43. Intensive blood-glucose control with sulphonylureas or insulin compared with conventional treatment and risk of complications in patients with type 2 diabetes (UKPDS 33). UK Prospective Diabetes Study (UKPDS) Group. Lancet 1998;352:837-853
44. Byford S, Leese M, Knapp M, Seivewright H, Cameron S, Jones V, Davidson K, Tyrer P: Comparison of alternative methods of collection of service use data for the economic evaluation of health care interventions. Health Econ 2007;16:531-536
45. Juul L, Sandbaek A, Foldspang A, Frydenberg M, Borch-Johnsen K, Lauritzen T: Adherence to guidelines in people with screen-detected type 2 diabetes, ADDITION, Denmark. Scand J Prim Health Care 2009;27:223-231
46. Zoffmann V, Harder I, Kirkevold M: A person-centered communication and reflection model: sharing decision-making in chronic care. Qual Health Res 2008;18:670-685

For peer review only

For peer review only - http://bmjopen.bmj.com/site/about/guidelines.xhtml

Figure 1:
Study design

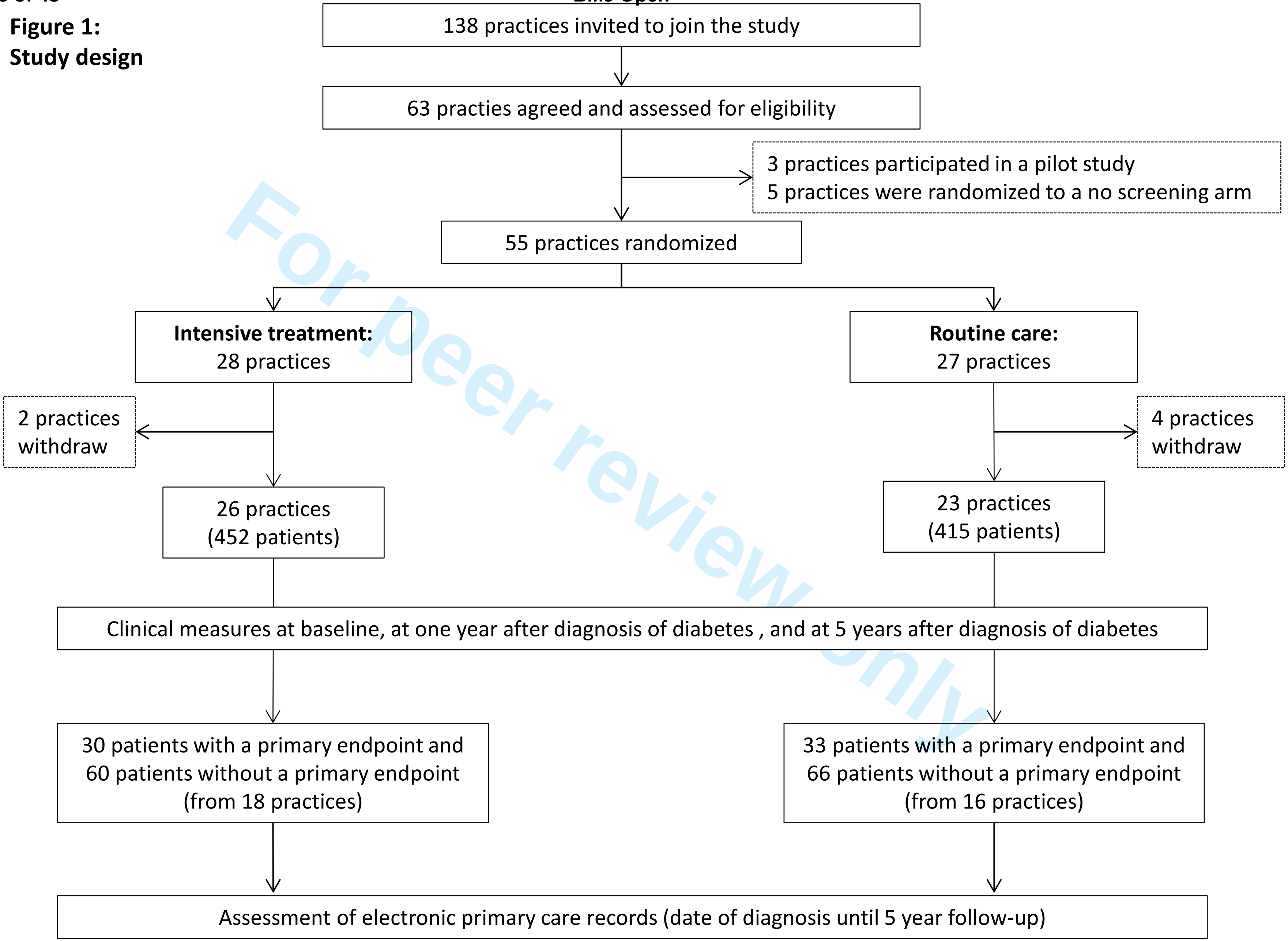

For peer review only

Figure 2:

Adjusted mean number(and 95%-CI) of contacts with GPs and nurses according to Intensive Treatment (grey) and Routine Care (black); stratified from year 1 to year 5 after diagnosis ǂ

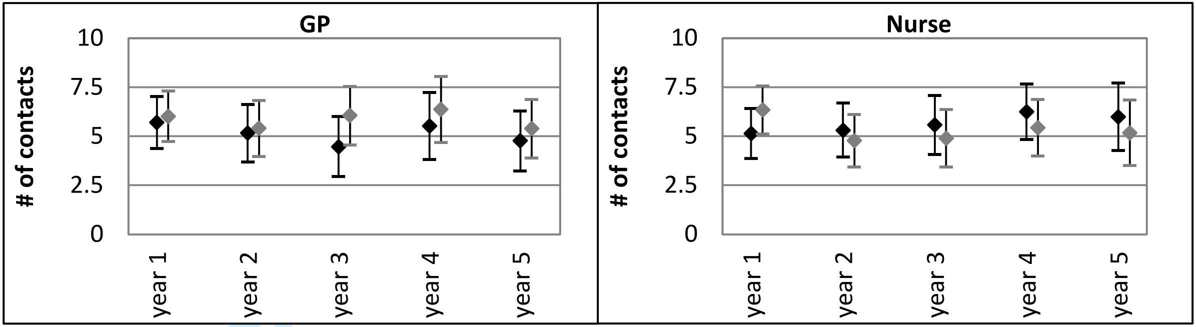

Overall adjusted mean number of contacts with GPs and nurses per year according to Routine Care and Intensive Treatment ⊦

	adj. mean (95%CI) ǂ		adj. mean (95%CI) ǂ
Intensive Treatment	5.80 (4.68, 6.93)	Intensive Treatment	5.34 (4.22, 6.47)
Routine Care	5.15 (4.01, 6.29)	Routine Care	5.49 (4.33, 6.65)
Difference (IT vs. RC)	0.65 (-0.95, 2.26)	Difference (IT vs. RC)	-0.15 (-1.77, 1.48)
time since diagnosis (years)	-0.05 (-0.24, 0.13)	time since diagnosis (years)	0.02 (-0.17, 0.21)
p-value (time x treatment)	0.513	p-value (time x treatment)	0.093

ƚ stratified linear regression models with a main effect for the intervention; adjusted for sex and age of diagnosis; accounted for patients being clustered within GP practices

⊦ overall linear regression models with a main effect for the intervention and for time since diagnosis and an interaction term between intervention and time; adjusted for sex and age of diagnosis; accounted for patients being clustered within GP practices and observations being clustered in patients

ǂ n=827 observations (n=169 in year 1, n=167 in year 2, n=168 in year 3, n=164 in year 4, and n=159 in year 5)

For peer review

Figure 3:

Proportion of patients receiving at least 4 prescriptions according to Intensive Treatment (grey) and Routine Care (black), stratified for year 1- 5 after diagnosis

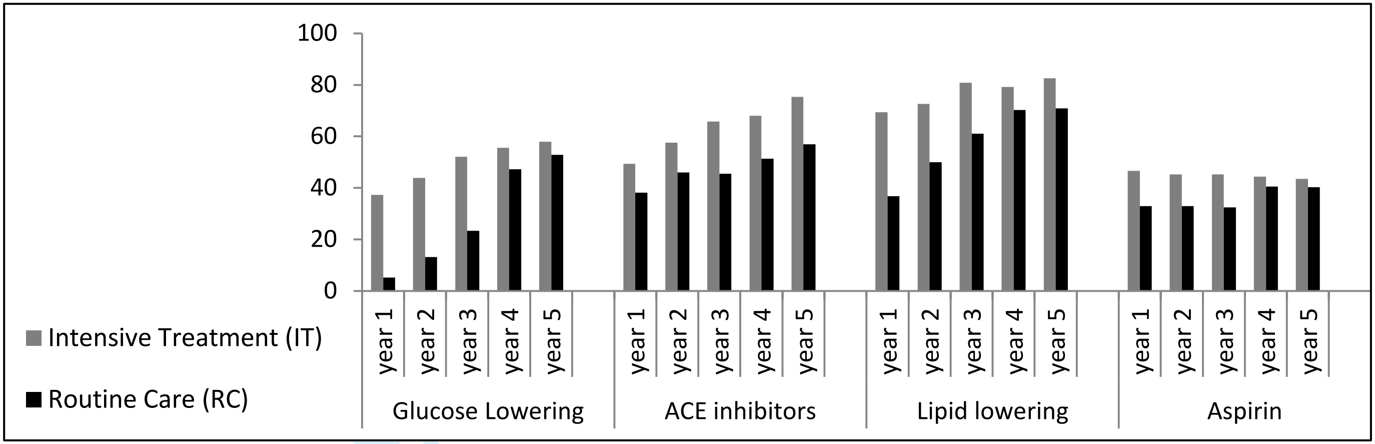

Odds Ratio of having received at least received 4 prescriptions per year IT vs. RC (reference)

Stratified by year ǂ	OR (95%-CI) ǂ	OR (95%-CI) ǂ	OR (95%-CI) ǂ	OR (95%-CI) ǂ
Year 1 (IT vs. RC)	10.89 (3.53, 33.56)	1.57 (0.73, 3.37)	4.00 (1.95, 8.20)	1.67 (0.72, 3.85)
Year 2 (IT vs. RC)	5.88 (2.51, 13.80)	1.60 (0.82, 3.09)	2.63 (1.31, 5.26)	1.66 (0.72, 3.86)
Year 3 (IT vs. RC)	3.78 (1.76, 8.10)	2.34 (1.18, 4.64)	2.63 (1.15, 6.01)	1.60 (0.62, 4.09)
Year 4 (IT vs. RC)	1.42 (0.73, 2.76)	2.06 (1.02, 4.14)	1.57 (0.68, 3.63)	1.16 (0.37, 3.61)
Year 5 (IT vs. RC)	1.23 (0.62, 2.42)	2.66 (1.14, 6.21)	1.99 (0.88, 4.53)	1.22 (0.43, 3.50)
**Year 1-5** ˫	**OR (95%-CI) #**	**OR (95%-CI) #**	**OR (95%-CI) #**	**OR (95%-CI) #**
Overall (IT vs. RC)	3.27 (1.81, 5.93)	2.03 (1.13, 3.65)	2.42 (1.3, 4.51)	1.41 (0.61, 3.24)
Time since diagnosis (per year)	1.61 (1.42, 1.83)	1.25 (1.12, 1.39)	1.33 (1.18, 1.5)	1.04 (0.93, 1.15)
p-value (time x treatment)	<.0001	0.331	0.131	0.220

ł stratified logistic regression models with a main effect for the intervention; adjusted for sex and age of diagnosis; accounted for patients being clustered within GP practices

˫ overall logistic regression models with a main effect for the intervention and for time since diagnosis and an interaction term between intervention and time; adjusted for sex and age of diagnosis; accounted for patients being clustered within GP practices and observations being clustered in patients

ǂ n=151 in year 1, n=149 in year 2, n=150 in year 3, n=146 in year 4, and n=141 in year 5

\# n=737 observations (n=151 in year1, n=149 in year 2, n=150 in year 3, n=146 in year 4, and n=141 in year 5)

Figure 4:
Proportion of patients who should receive medication according to the ADDITION protocol and national guidelines and the proportion of patients who receive medication within a 3 month time period at baseline, year 1 and year 5 after diagnosis [#]

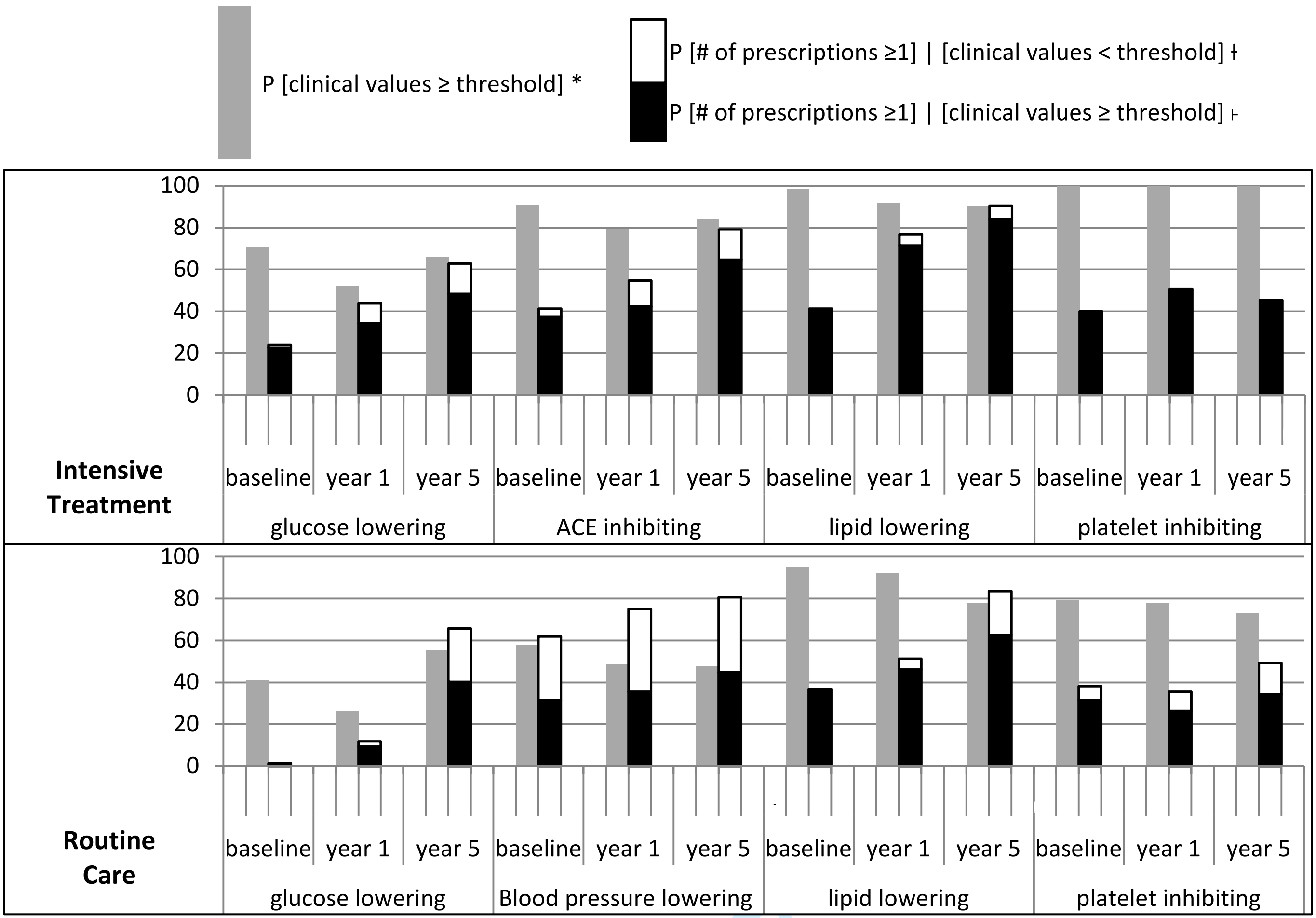

Prescription adherence (P [# of prescriptions ≥1] | [clinical value ≥ threshold])

IT-arm ˧	0.32	0.66	0.73	0.41	0.53	0.77	0.42	0.78	0.93	0.40	0.51	0.45
RC-arm ¥	0.03	0.35	0.73	0.55	0.73	0.94	0.39	0.50	0.81	0.40	0.34	0.47

[#] baseline, n=169; year 1, n=167; year 5, n=145
* i.e. medication indicated
ƚ i.e. either well controlled patients or those receiving medication without indication
˫ i.e. poorly controlled patients or those receiving indicated medication
˧ Adherence with ADDITION protocol; ¥ Adherence with national guidelines

Supplementary Material

Appendix 1: Proportion of patients receiving regular monitoring for HbA_{1c}, cholesterol and albuminuria and proportion of patients receiving blood pressure lowering medication

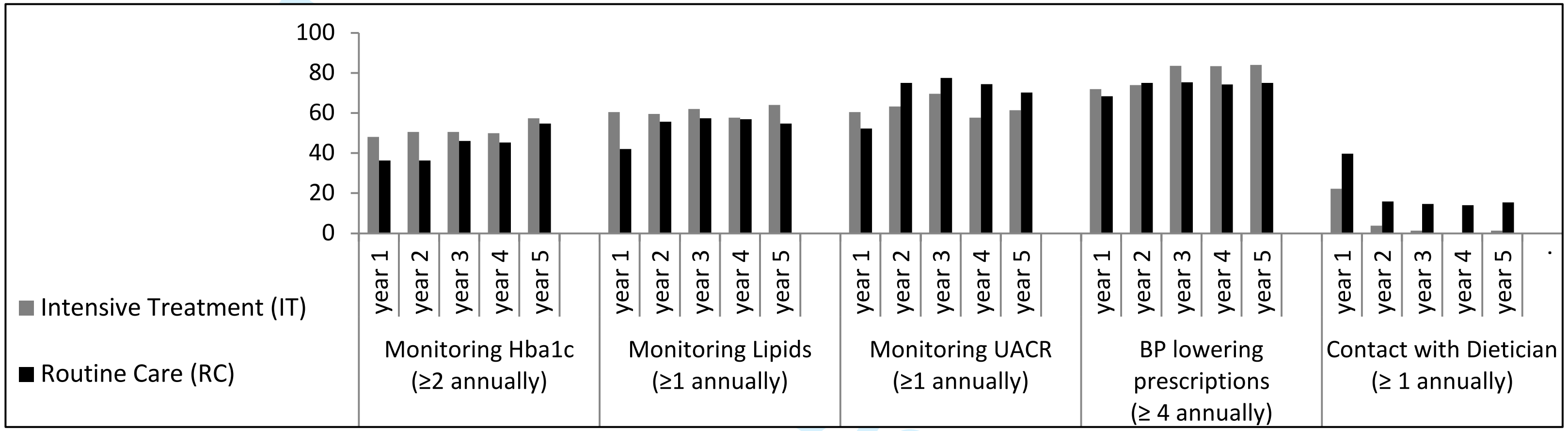

**Stratified by year ƚ**	**OR (95%-CI) ǂ**	**OR (95%-CI) ǂ**	**OR (95%-CI) ǂ**	**OR (95%-CI) #**	**OR (95%-CI) ǂ**
Year 1 (IT vs. RC)	2.00 (0.44, 9.02)	2.66 (0.53, 13.22)	1.41 (0.46, 4.32)	1.15 (0.55, 2.4)	0.88 (0.24, 3.26)
Year 2 (IT vs. RC)	2.29 (0.41, 12.63)	1.30 (0.23, 7.20)	0.62 (0.15, 2.60)	0.95 (0.45, 2.01)	0.38 (0.07, 2.18)
Year 3 (IT vs. RC)	1.28 (0.36, 4.52)	1.96 (0.36, 10.68)	0.93 (0.24, 3.56)	1.69 (0.72, 3.95)	0.12 (0.01, 1.39)
Year 4 (IT vs. RC)	1.52 (0.46, 5.03)	1.28 (0.29, 5.73)	0.49 (0.17, 1.45)	1.76 (0.72, 4.3)	-
Year 5 (IT vs. RC)	1.15 (0.44, 3.03)	2.3 (0.47, 11.32)	0.72 (0.30, 1.77)	1.89 (0.70, 5.15)	0.08 (0.01, 0.67)
**Year 1-5 ⱶ**	**OR (95%-CI) ¬**	**OR (95%-CI) ¬**	**OR (95%-CI) ¬**	**OR (95%-CI) Ⅎ**	**OR (95%-CI) ¬**
Overall (IC vs. SC)	1.56 (0.63, 3.83)	1.53 (0.51, 4.6)	0.82 (0.34, 1.98)	1.41 (0.71, 2.8)	0.43 (0.32, 0.58)
Time since diagnosis (years)	1.12 (1.01, 1.23)	1.05 (0.95, 1.16)	1.08 (0.97, 1.2)	1.15 (1.02, 1.31)	0.13 (0.04, 0.45)
p-value (time x treatment)	0.294	0.303	0.075	0.223	0.001

ƚ stratified logistic regression models with a main effect for the intervention; adjusted for sex and age of diagnosis; accounted for patients being clustered within GP practices

ⱶ overall logistic regression models with a main effect for the intervention and for time since diagnosis and an interaction term between intervention and time; adjusted for sex and age of diagnosis; accounted for patients being clustered within GP practices and observations being clustered in patients

ǂ n=169 in year 1, n=167 in year 2, n=168 in year 3, n=164 in year 4, and n=159 in year 5

n=151 in year1, n=149 in year 2, n=150 in year 3, n=146 in year 4 and n=141 in year 5

¬ n=827 observations (n=169 in year 1, n=167 in year 2, n=168 in year 3, n=164 in year 4, and n=159 in year 5)

Ⅎ n=737 observations (n=151 in year1, n=149 in year 2, n=150 in year 3, n=146 in year 4, and n=141 in year 5)

HbA_{1c} hemoglobin A1c; UACR urine-albumin-creatinine-ratio; BP blood pressure

For peer review only - http://bmjopen.bmj.com/site/about/guidelines.xhtml

Appendix 2: Results of various sensitivity analyses

	Adjusted odds ratio of having received 'continuous medication', IT vs. RC (reference)ɫ				Difference in adjusted mean number of contacts with GPs and nurses, IT vs. RC (reference) ˫	
	**OR (95-% CI)**				**adjusted mean difference (95%-CI)**	
	Glucose-lowering	ACE-inhibiting	lipid-lowering	aspirin	# of GP contacts	# of nurse contacts
main model (from Figure 2 & 3)	*3.27 (1.81, 5.93) ¬*	*2.03 (1.13, 3.65) ¬*	*2.42 (1.30, 4.51) ¬*	*1.41 (0.61, 3.24) ¬*	*0.65 (-0.95, 2.26)˫*	*-0.15 (-1.77, 1.48) ˫*
a) weighted model	2.89 (1.51, 5.53) ¬	2.13 (1.15, 3.93) ¬	2.54 (1.32, 4.92) ¬	1.47 (0.59, 3.69) ¬	0.81 (-0.79, 2.42) ˫	0.21 (-1.40, 1.81) ˫
b) multiple imputed model	3.06 (1.78, 5.28) ǂ	2.05 (1.20, 3.50) ǂ	2.37 (1.32, 4.25) ǂ	1.32 (0.62, 2.80) ǂ	0.68 (-0.9, 2.26) ǂ	-0.10 (-1.70, 1.50) ǂ
c) threshold: ≥ 2 prescriptions annually	3.07 (1.68, 5.61) ¬	2.10 (1.12, 3.94) ¬	2.16 (1.13, 4.14) ¬	1.45 (0.70, 3.02) ¬	-	-
d) threshold: ≥ 6 prescriptions annually	3.97 (2.17, 7.26) ¬	2.24 (1.25, 4.03) ¬	2.35 (1.24, 4.45) ¬	1.40 (0.57, 3.46) ¬	-	-
e) threshold: ≥ 12 prescriptions annually	4.86 (2.34, 10.1) ¬	1.79 (0.79, 4.06) ¬	1.35 (0.58, 3.12) ¬	1.04 (0.37, 2.97) ¬	-	-

ɫ overall logistic regression models with a main effect for the intervention and for time since diagnosis and an interaction term between intervention and time; adjusted for sex and age of diagnosis; accounted for patients being clustered within GP practices and observations being clustered in patients

˫ overall linear regression models with a main effect for the intervention and for time since diagnosis and an interaction term between intervention and time; adjusted for sex and age of diagnosis; accounted for patients being clustered within GP practices and observations being clustered in patients

¬ n=737 observations (n=151 in year1, n=149 in year 2, n=150 in year 3, n=146 in year 4, and n=141 in year 5)

ǂ n=885 observations (n=173 from year1 to year 5)

a) individuals weighted by the inverse probability of being in the sample given the status on the primary endpoint

b) multiple imputed dataset of participants with at least partially missing information on electronic primary care records in year 1 to 5 (PROC MI/PROC MIANALYZE)

c) threshold for 'continuous medication' changed to '≥ 2 prescriptions annually'

d) threshold for 'continuous medication' changed to '≥ 6 prescriptions annually'

e) threshold for 'continuous medication' changed to '≥ 12 prescriptions annually'

For peer review only - http://bmjopen.bmj.com/site/about/guidelines.xhtml

It needs to be acknowledged that the study does not report the primary or secondary outcomes of the trial (they have been reported elsewhere), but it reports the adherence of GPs to the trial protocol. Therefore, several points that are highly important in reporting the results of a trial are of inferior importance in reporting the adherence of GPs to the protocol.

CONSORT 2010 checklist of information to include when reporting a randomised trial*

Section/Topic	Item No	Checklist item	Reported on page No
**Title and abstract**			
	1a	Identification as a randomised trial in the title	1
	1b	Structured summary of trial design, methods, results, and conclusions (for specific guidance see CONSORT for abstracts)	3-4
**Introduction**			
Background and objectives	2a	Scientific background and explanation of rationale	5-6
	2b	Specific objectives or hypotheses	6
**Methods**			
Trial design	3a	Description of trial design (such as parallel, factorial) including allocation ratio	6-7
	3b	Important changes to methods after trial commencement (such as eligibility criteria), with reasons	Na
Participants	4a	Eligibility criteria for participants	6-7
	4b	Settings and locations where the data were collected	6-7
Interventions	5	The interventions for each group with sufficient details to allow replication, including how and when they were actually administered	7-8
Outcomes	6a	Completely defined pre-specified primary and secondary outcome measures, including how and when they were assessed	8-9
	6b	Any changes to trial outcomes after the trial commenced, with reasons	Na
Sample size	7a	How sample size was determined	6-7
	7b	When applicable, explanation of any interim analyses and stopping guidelines	Na
Randomisation:			
Sequence generation	8a	Method used to generate the random allocation sequence	Na
	8b	Type of randomisation; details of any restriction (such as blocking and block size)	Na
Allocation	9	Mechanism used to implement the random allocation sequence (such as sequentially numbered containers),	Na

For peer review only - http://bmjopen.bmj.com/site/about/guidelines.xhtml

concealment mechanism		describing any steps taken to conceal the sequence until interventions were assigned	
Implementation	10	Who generated the random allocation sequence, who enrolled participants, and who assigned participants to interventions	Na
Blinding	11a	If done, who was blinded after assignment to interventions (for example, participants, care providers, those assessing outcomes) and how	Na
	11b	If relevant, description of the similarity of interventions	na
Statistical methods	12a	Statistical methods used to compare groups for primary and secondary outcomes	9-10
	12b	Methods for additional analyses, such as subgroup analyses and adjusted analyses	9-10
**Results**			
Participant flow (a diagram is strongly recommended)	13a	For each group, the numbers of participants who were randomly assigned, received intended treatment, and were analysed for the primary outcome	Figure 1
	13b	For each group, losses and exclusions after randomisation, together with reasons	Figure 1
Recruitment	14a	Dates defining the periods of recruitment and follow-up	8
	14b	Why the trial ended or was stopped	Na
Baseline data	15	A table showing baseline demographic and clinical characteristics for each group	Table 1
Numbers analysed	16	For each group, number of participants (denominator) included in each analysis and whether the analysis was by original assigned groups	Figure 2-4
Outcomes and estimation	17a	For each primary and secondary outcome, results for each group, and the estimated effect size and its precision (such as 95% confidence interval)	Figure 2-4
	17b	For binary outcomes, presentation of both absolute and relative effect sizes is recommended	Na
Ancillary analyses	18	Results of any other analyses performed, including subgroup analyses and adjusted analyses, distinguishing pre-specified from exploratory	Na
Harms	19	All important harms or unintended effects in each group (for specific guidance see CONSORT for harms)	Na
**Discussion**			
Limitations	20	Trial limitations, addressing sources of potential bias, imprecision, and, if relevant, multiplicity of analyses	18-19
Generalisability	21	Generalisability (external validity, applicability) of the trial findings	18-19
Interpretation	22	Interpretation consistent with results, balancing benefits and harms, and considering other relevant evidence	Na
**Other information**			
Registration	23	Registration number and name of trial registry	4
Protocol	24	Where the full trial protocol can be accessed, if available	6 (ref 10)
Funding	25	Sources of funding and other support (such as supply of drugs), role of funders	20-21

For peer review only

For peer review only - http://bmjopen.bmj.com/site/about/guidelines.xhtml

*We strongly recommend reading this statement in conjunction with the CONSORT 2010 Explanation and Elaboration for important clarifications on all the items. If relevant, we also recommend reading CONSORT extensions for cluster randomised trials, non-inferiority and equivalence trials, non-pharmacological treatments, herbal interventions, and pragmatic trials. Additional extensions are forthcoming: for those and for up to date references relevant to this checklist, see www.consort-statement.org.

For peer review only

For peer review only - http://bmjopen.bmj.com/site/about/guidelines.xhtml

BMC Public Health

BioMed Central

Study protocol

Open Access

The ADDITION-Cambridge trial protocol: a cluster – randomised controlled trial of screening for type 2 diabetes and intensive treatment for screen-detected patients

Justin B Echouffo-Tcheugui[1], Rebecca K Simmons[1], Kate M Williams[2], Roslyn S Barling[2], A Toby Prevost[2], Ann Louise Kinmonth[2], Nicholas J Wareham[1] and Simon J Griffin*[1]

Address: [1]MRC Epidemiology Unit, Box 285, Addenbrooke's Hospital, Hills Road, Cambridge, CB2 0QQ, UK and [2]General Practice and Primary Care Research Unit, Department of Public Health and Primary Care, University of Cambridge, Cambridge, CB2 0SR, UK

Email: Justin B Echouffo-Tcheugui - basile.echouffo-tcheugui@mrc-epid.cam.ac.uk; Rebecca K Simmons - rebecca.simmons@mrc-epid.cam.ac.uk; Kate M Williams - kmw36@medschl.cam.ac.uk; Roslyn S Barling - ros.barling@gmail.com; A Toby Prevost - atp14@medschl.cam.ac.uk; Ann Louise Kinmonth - alk25@medschl.cam.ac.uk; Nicholas J Wareham - nicholas.wareham@mrc-epid.cam.ac.uk; Simon J Griffin* - simon.griffin@mrc-epid.cam.ac.uk

* Corresponding author

Published: 12 May 2009

BMC Public Health 2009, **9**:136 doi:10.1186/1471-2458-9-136

Received: 11 March 2009
Accepted: 12 May 2009

This article is available from: http://www.biomedcentral.com/1471-2458/9/136

© 2009 Echouffo-Tcheugui et al; licensee BioMed Central Ltd.
This is an Open Access article distributed under the terms of the Creative Commons Attribution License (http://creativecommons.org/licenses/by/2.0), which permits unrestricted use, distribution, and reproduction in any medium, provided the original work is properly cited.

Abstract

Background: The increasing prevalence of type 2 diabetes poses a major public health challenge. Population-based screening and early treatment for type 2 diabetes could reduce this growing burden. However, the benefits of such a strategy remain uncertain.

Methods and design: The *ADDITION-Cambridge* study aims to evaluate the effectiveness and cost-effectiveness of (i) a stepwise screening strategy for type 2 diabetes; and (ii) intensive multifactorial treatment for people with screen-detected diabetes in primary care. 63 practices in the East Anglia region participated. Three undertook the pilot study, 33 were allocated to three groups: no screening (control), screening followed by intensive treatment (IT) and screening plus routine care (RC) in an unbalanced (1:3:3) randomisation. The remaining 27 practices were randomly allocated to IT and RC. A risk score incorporating routine practice data was used to identify people aged 40–69 years at high-risk of undiagnosed diabetes. In the screening practices, high-risk individuals were invited to take part in a stepwise screening programme. In the IT group, diabetes treatment is optimised through guidelines, target-led multifactorial treatment, audit, feedback, and academic detailing for practice teams, alongside provision of educational materials for newly diagnosed participants. Primary endpoints are modelled cardiovascular risk at one year, and cardiovascular mortality and morbidity at five years after diagnosis of diabetes. Secondary endpoints include all-cause mortality, development of renal and visual impairment, peripheral neuropathy, health service costs, self-reported quality of life, functional status and health utility. Impact of the screening programme at the population level is also assessed through measures of mortality, cardiovascular morbidity, health status and health service use among high-risk individuals.

Discussion: *ADDITION-Cambridge* is conducted in a defined high-risk group accessible through primary care. It addresses the feasibility of population-based screening for diabetes, as well as the benefits and costs of screening and intensive multifactorial treatment early in the disease trajectory.

For peer review only

BMC Public Health 2009, **9**:136 http://www.biomedcentral.com/1471-2458/9/136

The intensive treatment algorithm is based on evidence from studies including individuals with clinically diagnosed diabetes and the education materials are informed by psychological theory. *ADDITION-Cambridge* will provide timely evidence concerning the benefits of early intensive treatment and will inform policy decisions concerning screening for type 2 diabetes.

Trial registration: Current Controlled trials ISRCTN86769081

Background

Diabetes is an increasingly common problem [1], associated with a substantial burden of premature mortality, morbidity, suffering and financial cost through its macrovascular and microvascular complications [2]. The high proportion (30–50%) of undiagnosed cases of diabetes [3], the large number of individuals with complications at clinical diagnosis [4], and the long (9–12 years) latent phase of the condition [5]. Indeed, type 2 diabetes fulfils many of the criteria for suitability for screening [6]. Adopting a national policy of population-based screening for type 2 diabetes could help to reduce the current burden of morbidity and mortality associated with the disease. However, there is continuing uncertainty about the benefits and costs of screening for type 2 diabetes. In particular, modelling data suggest that a key but uncertain determinant of the cost-effectiveness of screening is the size of cardiovascular risk reduction consequent on early intensive multifactorial treatment in screen-detected patients [7]. There is evidence that intensive multifactorial treatment is cost-effective in reducing cardiovascular morbidity and mortality in patients further along the disease trajectory with microalbuminuria [8,9]. It is also clear that intensive treatment of individual cardiovascular risk factors (blood pressure and cholesterol) is beneficial [10-14]. However, it is unclear to what extent intensive multifactorial treatment among screen-detected patients would be cost-effective. Intensive treatment of hyperglycaemia among patients with long-standing diabetes has not been associated with cardiovascular benefits [15-17]. However, long term follow-up of the UKPDS cohort showed that reducing levels of blood glucose from diagnosis led to fewer cardiovascular events [18]. It is unclear whether intensive treatment of hyperglycaemia during the lead time between clinical diagnosis and diagnosis by screening will be associated with similar benefits.

Ideally, there should be trial evidence of cost-effectiveness of screening programmes before they become public policy [6], as was the case for ultrasonographic screening for abdominal aortic aneurysm in men [19]. This is not yet the case for type 2 diabetes. TheAnglo-Danish-Dutch Study of Intensive Treatment In People with Screen Detected Diabetes in Primary Care(*ADDITION)* trial was set up in three countries: England (Cambridge and Leicester), Denmark and The Netherlands to provide evidence on screening for type 2 diabetes and the effects of early intensive multifactorial treatment [20]. We present the protocol of the Cambridge component of this trial.

Target population

If population-based screening for type 2 diabetes were to be undertaken, current evidence supports a targeted approach [6]. The *ADDITION-Cambridge* study targets people without known diabetes but at high risk of having prevalent undiagnosed type 2 diabetes, identified using a previously validated risk score [21]. This risk tool combines information routinely collected in primary care, including age, sex, body mass index and prescribed medication (steroids and antihypertensive drugs), to predict the presence of undiagnosed diabetes. This simple practical tool has previously been shown to perform reasonably well in different settings[22,23].

Limited evidence from previous studies

(i) The potential benefits and harms of screening

Earlier detection of diabetes and treatment of hyperglycaemia and related metabolic abnormalities may be beneficial. Screening for hyperglycaemia can identify patients at an early stage of the disease [24,25] who are likely to benefit from intensive treatment of cardiovascular risk factors. Patients who are given the label of diabetes may also benefit from becoming involved in a more organised and effective system of risk factor management [26]. However, it is uncertain whether an intervention to promote intensive multifactorial management for patients with screen-detected diabetes in primary care will be cost-effective. It is also unclear whether such an intervention might impact on the care of other patients with established diabetes and those at risk of diabetes in the primary care practices undertaking intensive treatment.

Concerns have been expressed about the psychological harms of screening programmes [27]. With the exception of one small randomised trial undertaken in the pilot phase of *ADDITION-Cambridge* [28], published data suggest no or limited psychological impact of screening for diabetes in newly detected individuals [29]. These data, which were mainly derived from cross-sectional or cohort studies (susceptible to selection and ascertainment bias) were recently confirmed by the results of a controlled trial embedded in *ADDITION-Cambridge* [30]. However, none of the published studies have examined the wider impact of screening on health related quality of life among high-

For peer review only - http://bmjopen.bmj.com/site/about/guidelines.xhtml

risk groups, the potential for a worsening of risk due to false reassurance, or the subsequent effects of intensive treatment on the quality of life of screen-detected individuals.

Despite screening negative for diabetes, some of the high-risk people targeted in a screening programme will exhibit a high cardiovascular risk profile and/or develop diabetes within a relatively short period of time given their high lifetime risk compared to the general population [31]. Screening and promotion of early multifactorial intensive treatment could therefore have a wider impact among high-risk individuals as well as those diagnosed with diabetes as a result of screening.

Little is known about the impact at the population level on mortality of a screening programme for diabetes. Modelling studies have suggested that 4–5 yearly screening programmes might be associated with a significant reduction in diabetes-related mortality in the order of 26–40% [32,33]. However, this needs to be confirmed in formal prospective studies.

(ii) The lack of trial evidence

Evaluations of screening that do not incorporate random allocation of representative population samples are particularly susceptible to misinterpretation and overestimation of benefits due to lead time, length time, spectrum, ascertainment and selection bias [34]. Evidence from randomised trials of the impact of screening is important for public health policy decisions in view of the extensive organisational, technical and financial inputs such a screening programme would demand. There is no trial evidence to suggest that early detection of type 2 diabetes improves outcomes, or that treatment effective for clinically diagnosed patients produces greater benefit when commenced in the lead time between detection by screening and clinical diagnosis.

ADDITION-Cambridge Objectives

The primary objective of the *ADDITION-Cambridge* study is to evaluate the effectiveness and cost-effectiveness of a stepwise screening programme for type 2 diabetes and intensive multifactorial treatment in people with screen-detected diabetes in English general practice.

The following research questions are posed:

- *Feasibility of screening*: What uptake and yield are achievable in a primary care-based stepwise screening programme for type 2 diabetes?

- *Costs of screening*: What are the health service and patient costs of screening for type 2 diabetes?

- *Early treatment of type 2 diabetes*: Can an optimised intensive intervention targeting blood glucose and associated cardiovascular risk factors reduce cardiovascular risk and mortality in people with screen-detected diabetes? Is this intervention cost-effective?

- *Population level impact:* Is a targeted screening programme for type 2 diabetes associated with reductions in population mortality and morbidity?

Methods and design

Design

ADDITION-Cambridge consists of two phases: a screening study and a subsequent treatment study. The screening phase examines the feasibility of a stepwise procedure to identify people with undetected diabetes and the effects of screening on health outcomes at the population level. The treatment study is a pragmatic single blind, cluster-randomised, parallel group trial comparing the effects of intensive multifactorial therapy with routine care (according to national guidelines) in individuals with screen-detected type 2 diabetes. The evaluation of the impact of the screening programme at the population level through the inclusion of random allocation of practices to a no screening (control) group is a feature specific to *ADDITION-Cambridge*. The study design, practice and patient flows are shown in Figures 1 and 2.

Ethical approval was obtained from the Cambridge (ref:01/063), Huntingdonshire (ref:00/609), Peterborough and Fenland (ref:P01/95), West Essex (ref:1511-0103), North and Mid Essex (ref:MH395 MREC02/5/54), West Suffolk (ref:03/002), and Hertfordshire and Bedfordshire (ref:EC03623) Local Research Ethics Committees, and the Eastern Multi-Centre Research Ethics Committee (ref:02/5/54). Written informed consent was obtained for all participants involved in both phases of the *ADDITION-Cambridge* study at the time of the diabetes screening appointment and subsequent diagnostic test.

ADDITION-Cambridge is registered as ISRCTN86769081. The ClinicalTrials.gov Identifier of the whole *ADDITION* Study that includes England (Cambridge and Leicester), Denmark and the Netherlands is NCT00237549.

Setting

Patients were recruited from general practices in urban, suburban and rural Cambridgeshire, East Hertfordshire, West Suffolk and North Essex areas of England.

Practice recruitment

Figure 1 shows the flow of practice recruitment. 138 practices were invited to take part in the study between Sep-

For peer review only - http://bmjopen.bmj.com/site/about/guidelines.xhtml

BMC Public Health 2009, **9**:136 http://www.biomedcentral.com/1471-2458/9/136

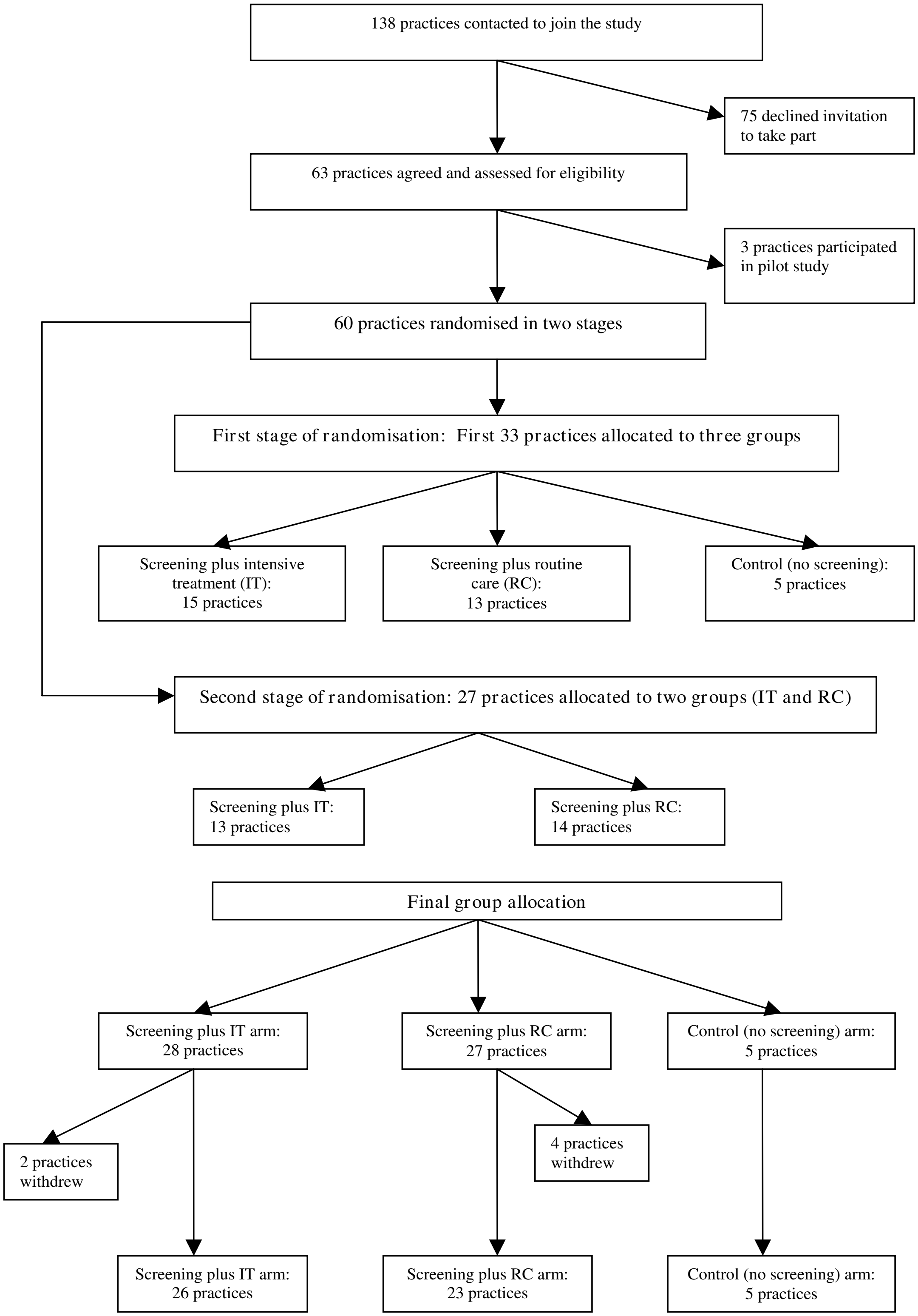

Figure 1
Practice recruitment and randomisation in the *ADDITION-Cambridge* study.

BMC Public Health 2009, **9**:136

http://www.biomedcentral.com/1471-2458/9/136

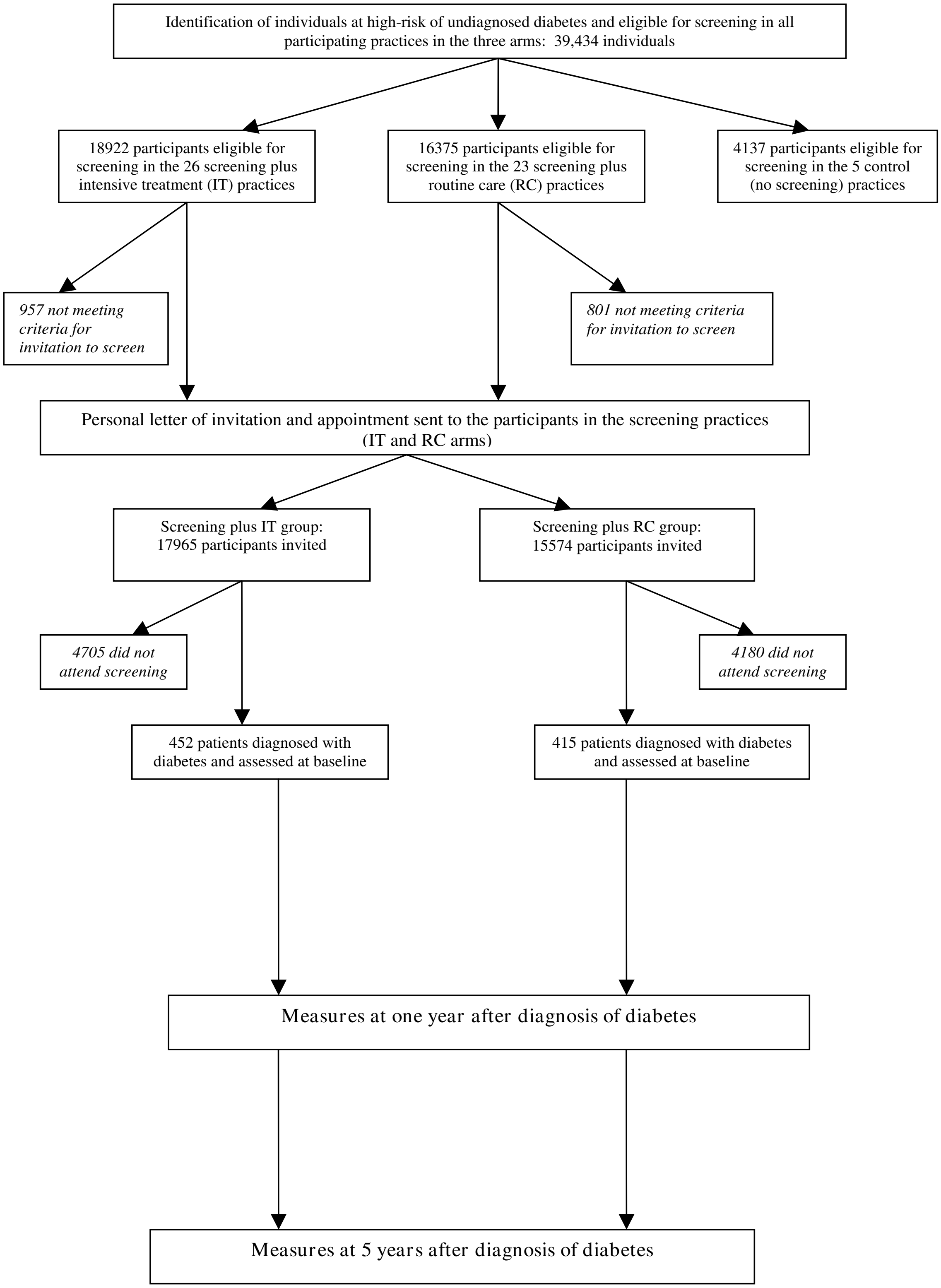

Figure 2
Participant recruitment in the *ADDITION-Cambridge* study.

BMC Public Health 2009, 9:136 http://www.biomedcentral.com/1471-2458/9/136

tember 2001 and August 2003. Personalised letters were sent to the practice manager, partners and nursing staff in each surgery highlighting the importance of the study to primary care, the involvement of practice staff and the reimbursement of all costs involved. We enclosed a brief summary of the study and a Research Information Sheet for Practices [35]. A principal investigator and member of the trial team visited interested practice teams to discuss the study in further detail. All relevant practice staff were encouraged to attend, particularly those that would be involved in the administration of the screening programme. Practices were eligible if they were able to provide data for the calculation of the diabetes risk score for at least 70% of their patients, a criterion satisfied by all 63 practices that agreed to take part.

Three practices undertook pilot testing of the screening strategy, the baseline measures and the intensive treatment materials and training. The remainder (60 practices) were allocated to the three study arms. In the participating practices, a "set-up" visit was undertaken to deliver practice study manuals, to provide the software developed to assist with monitoring the progress of the screening programme and recording of blood glucose test results, and to train the staff in logistical and technical aspects of screening. Further visits were arranged for practices allocated to screening followed by intensive treatment to provide the materials and training to enable them to deliver the intervention.

Practice randomisation

Randomisation of practices was completed centrally and independently of the trial co-ordination team immediately following recruitment. The project statistician used a partial minimisation procedure that dynamically adjusted the randomisation probabilities to balance important baseline practice variables: the number of patients with known diabetes (<160 and ≥ 160 patients) and the local district hospital (Addenbrooke's, Hinchingbrooke, Peterborough, Kings Lynn, Broomfield, Stevenage and Bury St Edmunds hospitals). The first 33 practices recruited were allocated in a ratio of 1:3:3 to the following arms: control (no screening), screening followed by intensive multi-factorial treatment of diabetes (IT), and screening plus routine care of diabetes according to national guidelines (RC). Allocation of practices to the control (no screening) group was stopped at N = 5. The need to increase the yield of individuals with diabetes for the treatment trial warranted the uneven randomisation ratio with a disproportionate number of screening practices and a second stage of randomisation. 27 practices were subsequently randomised in a ratio of 1:1 to IT (n = 13) and RC (n = 14). The final group allocation after the two stages of randomisation included 28 practices to IT, 27 practices to RC and 5 practices to control (no screening). Six of the 60 randomised practices (2 IT and 4 RC) dropped out following recruitment, but before screening commenced due to other commitments or unforeseen difficulties in setting up the practice-based screening programme.

Phase one: step-wise screening programme

(i) Eligibility for screening

Individuals eligible for an invitation for screening were people registered with one of the participating general practices, aged 40 to 69 years, not known to have diabetes and with a diabetes risk score of >0.17 (corresponding to the top 25% of the population distribution). In screening practices, eligible participants deemed unfit for screening by their general practitioner were not invited for biochemical testing. Exclusion criteria, also assessed initially by the participating general practitioners, included pregnancy, lactation, an illness with a likely prognosis of less than one year or a psychiatric illness likely to limit study involvement or invalidate informed consent.

(ii) Participant recruitment

Figure 2 outlines the recruitment procedure. Participants were recruited through their local general practice. An electronic search of medical records was undertaken for routinely collected information that would allow the calculation of a diabetes risk score for each patient [21]. Information about the diabetes risk score was withheld from practitioners in the control practices.

Figure 3 outlines the screening and diagnostic tests used to identify people with undiagnosed diabetes. In practices in the RC and IT groups, general practitioners wrote to all high-risk patients, enclosing a study information sheet, and inviting them to attend the practice for random capillary blood glucose (RBG) and capillary glycosylated haemoglobin (HbA_{1c}) tests, after initial consent had been obtained. The letter was sent at least two weeks in advance of the scheduled appointment. Patients were advised to telephone the surgery and arrange an alternative appointment if the original was inconvenient. One reminder letter was sent to non-attendees. Participants with an RBG of ≥ 11.1 mmol/l were invited for a standard 75 g oral glucose tolerance test (OGTT) at one of four outpatient facilities. Those with an RBG of 5.5–11.0 mmol/l were invited to return to the practice for a fasting capillary blood glucose (FBG) test. Those with an FBG of ≥ 6.1 mmol/l, or an FBG of 5.5–6.0 mmol/l together with an HbA_{1c} of ≥ 6.1%, were invited for an OGTT. The RBG, FBG and OGTT were conducted on different days. Participants with an FBG of 5.5–6.0 mmol/l and an HbA_{1c} of ≥ 6.1% who had a positive OGTT underwent a second confirmatory OGTT on a different day. World Health Organisation criteria were used to diagnose diabetes [36]. Practitioners were informed by fax about the result of clinical and biochemical measures with a clear statement of whether or not the

For peer review only - http://bmjopen.bmj.com/site/about/guidelines.xhtml

BMC Public Health 2009, **9**:136 http://www.biomedcentral.com/1471-2458/9/136

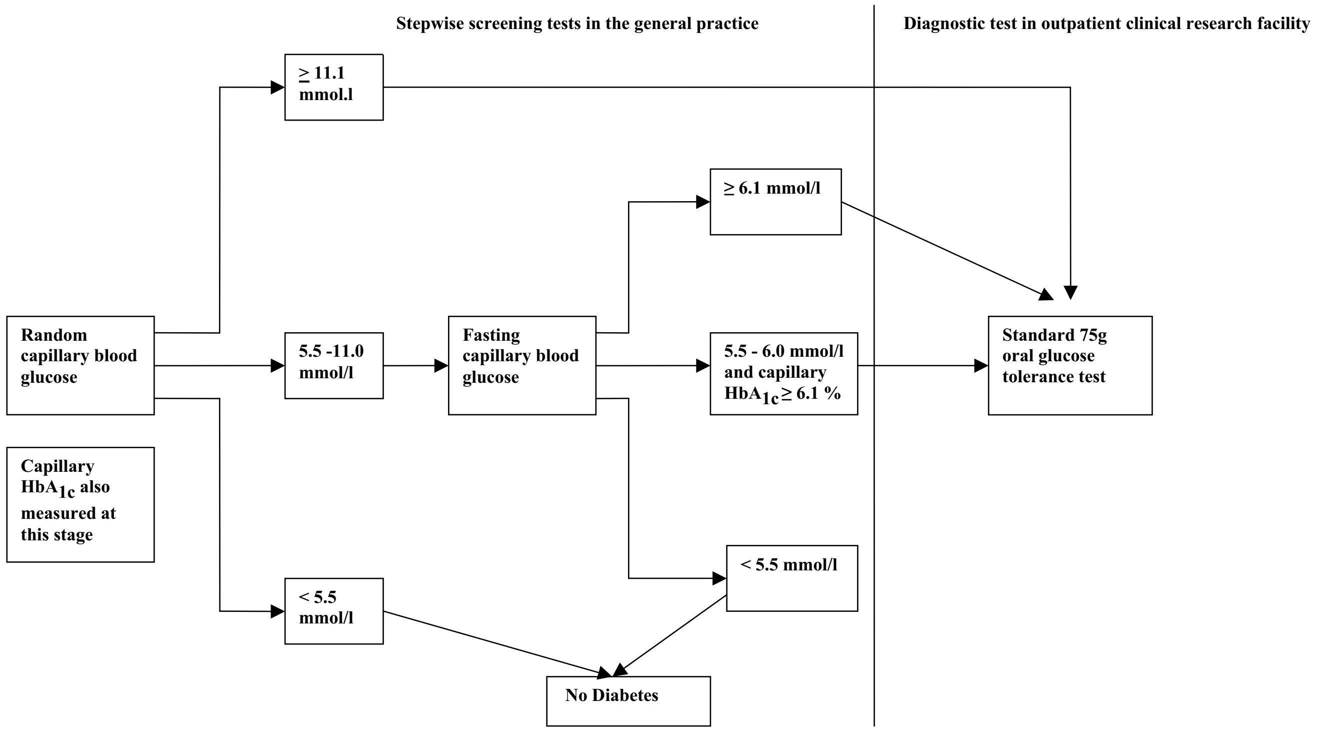

Figure 3
Screening algorithm used in the *ADDITION-Cambridge* study.

individual met diagnostic criteria for type 2 diabetes. The general practitioner or a practice nurse then informed the patient of the test results.

In the 54 participating practices (including the five control practices), 39,434 people aged 40–69 years were at high risk of prevalent undiagnosed diabetes. In the 49 screening practices, 35,297 individuals aged 40–69 years were at high-risk. 33,539 patients were invited for the first stage of screening (RBG and HbA_{1c}) and 24,654 (73.5%) attended this appointment.

(iii) Outcomes

These include the number of high-risk individuals presenting for screening, the number of people with newly diagnosed type 2 diabetes, the psychological status of people invited for screening, metabolic status, cardiovascular risk and self-perceived health in people with newly-diagnosed type 2 diabetes, and health service and patient costs. In addition we will assess the population effects of the screening programme by comparing high-risk individuals in the three study groups (IT, RC and control) using the following measures: mortality, self-reported cardiovascular morbidity, health status, health utility and lifestyle changes (self-reported diet, physical activity and smoking status). Mortality will be assessed on all high-risk individuals, while other measures will be collected in a random sample of the high-risk population (in each of the three groups: IT, RC and control) using a postal questionnaire. All the high-risk participants in the three study arms are tagged at the Office of National Statistics (ONS) for mortality, following approval under section 60 of the UK Health and Social Care Act 2001 (Reference MR798).

Phase two: trial of intensive multifactorial treatment in people with screen-detected diabetes

(i) Intervention

Participants are treated routinely or intensively depending on the study arm to which their practice was randomised (RC or IT). The intensification of diabetes management is achieved through the addition of the following features to the existing diabetes care within IT practices:

- Funding of practices to facilitate more frequent contact between patients and practitioners. The recommended frequency of consultation was one 30-minute annual review for each patient, three additional 10-minute consultations with a GP and three with a nurse, per year for the first three years after diagnosis, over and above the usual consultation frequency for a patient aged 40–69 years.

For peer review only - http://bmjopen.bmj.com/site/about/guidelines.xhtml

• Recommendation to the GPs to refer all newly diagnosed patients to a dietitian

• A practice-based academic detailing session for practitioners conducted by a local consultant diabetologist and a general practice opinion leader to describe the treatment algorithms and targets, patient materials, and present the evidence underpinning intensive treatment. The treatment algorithms (Table 1) were based on trial data demonstrating the benefits of intensive treatment of several cardiovascular risk factors in people with diabetes [8,13]. All treatment recommendations were for medications within their existing licensed indications. GPs were advised to consider prescribing an angiotensin converting enzyme (ACE) inhibitor to patients with a blood pressure ≥ 120/80 mmHg and a previous cardiovascular event or at least one cardiovascular risk factor other than diabetes [13]. The rest of the intervention is based on the stepwise regimen from the Steno-2 study [8] aimed at optimising hyperglycaemia, hypertension, dyslipidaemia and microalbuminuria. As per the Steno-2 regime, GPs were advised to consider prescribing 75 mg of aspirin daily to all patients without specific contraindications. Although targets for treatment are specified and classes of drugs recommended, where there is a choice of individual agents the decision is made by the GPs and patients. The intensive treatment protocol was reviewed after the publication of the Heart Protection Study [12] to include the prescription of statins to all screen-detected patients with a cholesterol level of ≥ 3.5 mmol/l.

• Two interactive practice-based audit and feedback sessions were undertaken, including feedback of the overall performance of the practice against the treat-

Table 1: Treatment recommendations in the intensive treatment arm

	Target	Baseline	2 months If above target	4 months If above target	6 months If above target	9 months If above Target	12 months If above target
HbA1c	**<7.0%**	**Diet**	HbA1c >6.5% **Metformin** (avoid using metformin if creatinine level >130 μmol/L)	HbA1c >6.5% add a second medication **Metformin or PGR or SU or TZD**	HbA1c >6.5% add a third medication **Metformin or PGR or SU or TZD**	Continue oral hypoglycaemic medication and consider adding insulin	As for 9 months
Blood Pressure	**≤ 135/ 85mmHg**	>120/80 mmHg or CVD+ **ACE Inhibitor** titrated to maximum dose	If bp >135/85 mmHg Add a **Thiazide diuretic or Ca Antagonist** (Change **ACE to ACE2** if creatinine >130 μmol/L or K+ >5.0 mmol/L or intolerable side effects)	As for 2 months	If bp >135/85 mmHg **Add β blocker orα Blocker**	As for 6 months	As for 6 months
Cholesterol †IHD-	**<5.0mmol/l**	Chol ≥ 3.5 mmol/l, **diet & statin**	Chol >5.0 mmol/l **Increase statin dose up to maximum** (If CK> 1800 U/L, stop statin)	As for 2 months	Consider adding a **fibrate** if Chol >5.0 mmol/l	As for 6 months	As for 6 months
Cholesterol IHD+	**<4.5mmol/l**	chol ≥ 3.5 mmol/, **diet & statin**	Chol >4.5 mmol/l **Increase statin dose up to maximum** (If CK> 1800 U/L, stop statin)	As for 2 months	Consider adding a **fibrate** if Chol >5.0 mmol/l	As for 6 months	As for 6 months
Acetylsalycilic acid	75 mg of aspirin daily to all patients without specific contraindications						

SU = Sulphonylurea, PGR = Prandial glucose regulator, ACE = angiotensin converting enzyme, TZD = thiazolinedione, ACE2: Angiotensin- II receptor Antagonist, K+: potassium, Ca: Calcium, IHD- = no history of ischaemic heart disease, IHD+ = history of ischaemic heart disease, CVD+ = Previous cardiovascular event or presence of cardiovascular risk factor other than diabetes, bp = blood pressure, Chol = cholesterol

For peer review only - http://bmjopen.bmj.com/site/about/guidelines.xhtml

ment targets, the optimisation of the management of individual patients and the reiteration of the treatment targets. These were organised by the same opinion leaders at six and 14 months after the initial education session.

• Provision of glucometers for patients and any necessary training in their use for practitioners. The decision to offer a glucometer or not to a patient was left to the practitioner.

• Practice teams were provided with a pack of theory-based educational materials (Getting Started with Diabetes) to give to patients at diagnosis. The materials provide a shared framework on the causes, consequences and treatment of diabetes. The materials were developed by a multidisciplinary team and drew on Leventhal's self regulation model, a social cognition model from psychology [37]. They cross-referred to 'Diabetes for Beginners-Type 2' a Diabetes UK publication [38] that was included in the patient's pack. Specifically, participants were encouraged (i) to try to lose 5–10% of their body weight (relevant if BMI>28 kg/m^2 with a target of 0.45 kg/week) through a combination of diet and physical activity; (ii) to increase their physical activity gradually (the goal was to reach the equivalent of 35 minutes of brisk walking per day for 7 days per week); (iii) to avoid excessive alcohol intake; (iv) to take their medication regularly; (v) to self-monitor their blood glucose level if given a glucometer by their practice (the targets for self-monitored blood glucose are < 9 mmol/l 90 minutes after meals, and < 6 mmol/l before meals), and: (vi) to attend annual checks. Participants who smoked were encouraged to stop.

In the RC arm, participants with screen-detected diabetes receive the normal pattern of diabetes care as delivered through the UK National Health Service (NHS) according to current recommendations.

(ii) Endpoints

Primary endpoints: *At one year follow-up* the principle outcome is modelled 10-year risk of cardiovascular events derived using the UKPDS risk engine [39]. The UKPDS model uses information on sex, ethnicity, smoking status, presence or absence of atrial fibrillation, systolic blood pressure, HbA_{1c}, total cholesterol, and HDL-cholesterol to predict the 10-year risk of primary CVD. Predicted events are myocardial infarction, sudden cardiac death, other incident ischaemic heart disease, stroke, and peripheral vascular disease death. *At five-year follow-up*, the primary endpoint is a composite of cardiovascular mortality and morbidity (non-fatal myocardial infarction, non-fatal stroke, non-traumatic amputations and revascularisations). ***Secondary endpoints*** are all-cause mortality, development or progression of renal impairment, peripheral neuropathy, blindness, reduced visual acuity, macular oedema, retinopathy; health status, health utility, quality of life, anxiety, well-being, treatment satisfaction, health service costs (number of visits to general practitioners and hospital doctors for outpatient clinics, hospital admissions and prescribed medications). ***Intermediate endpoints*** are self-reported smoking status, diet, physical activity behaviour and medication adherence, HbA_{1c}, total cholesterol, LDL-cholesterol, HDL-cholesterol, triglycerides, blood pressure, modelled 10-year cardiovascular risk (at five-year follow-up), self reported hypoglycaemic episodes, microalbuminuria, body mass index and plasma vitamin C.

Measurement

Table 2 shows the distribution across time of measures relating to the screening procedure and the treatment phase of the study. Baseline measurements were carried out on all patients eligible for an OGTT following the screening phase of the study. These included the completion of questionnaires, physiological and anthropometric measures and venesection. Similar measurements are conducted at one year and five years after diagnosis, without repetition of the OGTT. The measurements at baseline, one-year and five-year follow-up are undertaken at outpatient clinical research facilities by trained staff following standard operational procedures and unaware of participants' study group allocation. Questionnaires are used to collect information on basic demographics, health behaviours, health utility, functional status and costs.

Health behaviours

Smoking status, alcohol consumption, and medication adherence are assessed by questionnaire. Medication adherence is assessed by the Medication Adherence Report Schedule (MARS) questionnaire [40]. Physical activity is assessed using the EPAQ2 [41] and IPAQ [42] questionnaires. Dietary intake is evaluated using a validated food frequency questionnaire [43].

Health utility, functional status, quality of life, well-being, treatment satisfaction and anxiety

The generic and disease-specific instruments used are diabetes well-being questionnaire (W-BQ12) [44], SF-36 [45], SF-8 [46], Audit of Diabetes-Dependent Quality of Life (ADDQoL) [44], diabetes treatment satisfaction (DTS) [44], and EuroQol (EQ-5D) [47], consultation and relational empathy (CARE) measure [48] and the Spiegelberger Short form State Anxiety Inventory [49].

Costs

Personal patient costs to attend initial screening tests and health service use in the three months prior to follow-up

For peer review only - http://bmjopen.bmj.com/site/about/guidelines.xhtml

BMC Public Health 2009, **9**:136 http://www.biomedcentral.com/1471-2458/9/136

Table 2: Measures used at baseline, one-and five-years in the *ADDITION-Cambridge*

Measures	Baseline			1-year			5-year		
	C	RC	IT	C	RC	IT	C	RC	IT
**Diabetes risk score calculation**	X	X	X						
**Questionnaires measures**									
1. Ethnic group, occupation, educational level and social class		X	X						
2 Smoking status, alcohol consumption		X	X		X	X	X	X	X
3. Rose angina questionnaire [51]		X	X				X		
4. Self-reported history of angina, heart attack and stroke		X	X		X	X	X	X	X
5. Medication adherence:									
All drugs during the last month [40]		X	X		X	X		X	X
Hypoglycaemic drugs during the last month [40]					X	X		X	X
6. EuroQoL EQ-5D [47] & SF-36 [45]/SF-8 [46]		X	X		X	X	X	X	X
7. Diabetes related quality of life: ADDQoL [44], Diabetes well-being: W-BQ 28 [44], Diabetes treatment satisfaction: DTSQ[44]					X	X		X	X
8. Spiegelberger Short form State Anxiety inventory [49]		X	X		X	X			
9. Consultation and relational empathy measure: CARE [48]		X	X		X	X			
10. Diabetes knowledge †					X	X			
11. EPAQ-2 [41]		X	X		X	X		X	X
12. IPAQ [42]		X	X		X	X	X	X	X
13. EPIC food frequency questionnaire [43]		X	X		X	X		X	X
14. Brief dietary questionnaire (adapted from the EPIC food frequency questionnaire) †							X	X	X
15. Costs comprising:									
Personal patient costs †		X	X						
Health Service and medication use previous 3 months (adapted from the Aberdeen Health Service Research Unit questionnaire) †					X	X	X	X	X
16. Neuropathy questionnaire (adapted from the Michigan Screening Instrument) †		X	X		X	X		X	X
**Biological measures**									
17. Waist circumference, height, weight, blood pressure, body fat impedance and ECG		X	X		X	X		X	X
18. Fasting capillary blood glucose		X	X						
19. Fasting, 30 and 120 min: venous whole blood glucose (OGTT), plasma glucose, plasma insulin.		X	X						
20. HbA_{1c}, total cholesterol, HDL and LDL cholesterol, triglyceride, Vitamin C, Urinalysis, Urine albumin/creatinine ratio, Urea and Electrolytes, Creatinine, Albumin, Biliribin, Alanine Amino Transferance (ALT), Alkaline Phosphatase, Aspartate Amino Transferase (AST), Thyroid Stimulating Hormone (TSH)		X	X		X	X		X	X
21. Modelled CVD risk calculated with the UKPDS risk engine [39]		X	X		X	X		X	X
22. Stereoscopic fundal photography								X	X
23. Mortality							X	X	X

†: Questionnaire developed for the study

are quantified using an adapted version of the Health Services Research Unit Aberdeen questionnaire that inquires about the use of services (consultations with healthcare professionals and hospitalisations) and medications [50].

Angina is assessed using the Rose angina questionnaire [51]. Neuropathy is evaluated using an adapted version of the Michigan Neuropathy Screening Instrument [52].

Physiological measures

Random and fasting capillary blood glucose concentrations were assessed by Hemocue (β-HemoCue AB, Angelholm, Sweden). The venous plasma blood glucose level is assessed by the glucose dehydrogenase method and read photochromatically. The stability of the analyser was checked daily and external calibration with the Hemocue quality assurance scheme was undertaken monthly. HbA_{1c} was analysed in capillary blood samples from general practices using the Bio-Rad® system and in venous samples at the time of diagnostic testing by ion-exchange high-performance liquid chromatography on a Tosoh machines (Tosoh Bioscience, Redditch, UK). Serum total cholesterol, HDL-cholesterol and triglycerides are measured by means of enzymatic techniques (Dade Behring Dimension analyser, Newark, USA). Plasma creatinine is analysed with kinetic colorimetric methods, urine albumin by rate nephelemetry (Dade Behring Nephelometer II, Newark, USA). Plasma levels of urea and electrolytes, bilirubin, alanine aminotansferase (ALT), aspartate aminotransferase (AST), alkaline phosphatase, and thyroid stimulating hormone (TSH) and urine levels of creatinine are assayed by means of the Dade Behring Dimension analyser. Plasma vitamin C level was measured with a Fluoroskan Ascent FL fluorometer. The albumin-to-creatinine ratio (ACR) is measured on a random spot urine specimen. For assays requiring fasting, participants attend after a 10-hour fast.

For peer review only - http://bmjopen.bmj.com/site/about/guidelines.xhtml

Anthropometry
Blood pressure is calculated as the mean of three measurements performed after at least 10 minutes rest, while participants are seated with the cuff on the predominant arm at the level of the heart, using an automatic sphygmomanometer (Omron M4, UK). ECG is recorded by a 12 lead machine. Body height and weight are measured in light indoor clothing and without shoes using a fixed rigid stadiometer and a scale (SECA, UK) respectively. Waist circumference is estimated as the average of two measurements taken with a tape measure halfway between the lowest point of the rib cage and the anterior superior iliac crests when standing. Body fat percentage is measured by bio-electrical impedance (TANITA, Tokyo, Japan).

Ascertainment of mortality and cardiovascular morbidity
Macrovascular and microvascular events in patients with screen-detected diabetes will be ascertained by a combination of strategies including electronic READ code searches of medical records for events, and notes extraction on potential cases of events. Anonymous case reports packs will be prepared by a member of the research team unaware of participants study group allocation for independent review of each potential event by an endpoint committee also unaware of study group allocation. All patients will also have an ophthalmologic evaluation including stereoscopic fundal photography at the five-year assessment. Fundal photography will be assessed by a separate endpoint committee blind to study groups. ICD-10 coded mortality data is reported periodically by the ONS for all high-risk participants in the three arms.

Assessment of the effect of screening in a random sample of people at high risk of prevalent undiagnosed diabetes in each of the three study groups (IT, RC and control) will be undertaken by postal questionnaire in 2009, six years on average post randomisation. This questionnaire includes demographic characteristics, self-reported history of angina, heart attack and stroke, self-reported smoking status, IPAQ, simple dietary behaviour questions, EuroQoL (EQ-5D), Short Form-8 (SF-8), and the adapted version of the Health Services Research Unit Aberdeen questionnaire for the use of medication and services.

Costs of the intervention
The economic analysis will establish the NHS costs of the initial screening programme for type 2 diabetes from a patient and health service perspective. We will examine the cost-effectiveness of the multifactorial intensive treatment of patients with screen-detected type 2 diabetes from a health service perspective.

Participant retention
The retention rate at one year follow-up was 85%. In order to maximise retention, we are reimbursing patients' travel at follow-up assessment. We have also been sending annual Christmas cards to all participants. A few months before the start of the five-year assessment, we will send a newsletter to all participants outlining the one-year results and plans for inviting them back for re-measurement.

Participant safety
Screening equipment was enrolled in the HemoCue quality assurance programme. The glucose tolerance test was undertaken by trained staff in dedicated testing centres. Treatment algorithms have been developed with advice from local diabetes specialists who also contributed to the initial and follow-up practice-based training sessions for primary care staff involved in diabetes care. The responsibility for prescribing and management decisions remains with the general practitioners. Classes of medication are only recommended within licensed indications.

In Cambridge, an independent Trial Steering Committee meets regularly and makes recommendations on ethical or safety aspects. At the European level, a Data Monitoring and Ethics Committee receives periodic reports on deaths and hypoglycaemic episodes. Termination of the study would be determined on the basis of mortality. Based on general trials stopping rules, it was suggested that the first interim analysis blind to study group (using data from the three countries) be undertaken when the total number of deaths reaches 200. The rule for termination is a significant difference in mortality between the IT and RC groups at a level of significance of 0.001.

Statistical procedures
Analysis
(i) Effect of intensive multi-factorial treatment
Analysis will be by intention-to-treat allowing for clustering of patients by practice. This will be supported by sensitivity analyses, assuming a range of outcomes for non-completers informed by baseline data. The main analyses will compare outcomes between patients with screen-detected diabetes receiving routine care (RC) and those receiving intensive treatment (IT), adjusting for differences in baseline variables. The primary perspective for cost analysis will be the health service.

At one year comparisons will be made on modelled 10-year cardiovascular disease (CVD) risk [39] and on secondary outcomes including individual cardiovascular risk factors, health utility, functional health status, and costs. The costs of the intensive intervention will then be compared with unit change in health utility. At five-years, analyses will include comparisons of main outcomes (fatal and non-fatal macrovascular events) and secondary outcomes (microvascular events, individual cardiovascular risk factors, all-cause mortality, health utility, functional health status, and costs).

For peer review only - http://bmjopen.bmj.com/site/about/guidelines.xhtml

(ii) Population effects of screening

People at high risk of having undiagnosed diabetes in the screening practices (IT and RC) will be compared to those in the no screening (control) practices to assess the impact of screening on mortality, cardiovascular morbidity, health status, self-reported diet, physical activity and health service costs using ONS and questionnaire data. This will be done using an intention to screen analysis. For the mortality analysis the primary outcome will be all-cause mortality and the secondary outcomes cardiovascular, cancer and other causes of mortality. Mortality, cardiovascular morbidity, health status, diet, and physical activity among people at high risk of having undiagnosed diabetes will also be compared between IT and RC groups in an intention to treat analysis to quantify the potential wider benefits of the practice-based intensive treatment intervention package.

Sample size

The sample size calculation was based on estimates of uptake and prevalence of undiagnosed diabetes from the Ely study between 1990 and 1992 [53]. IT vs. RC comparison of individual risk factors was originally based on 1000 screen-detected patients (500 in the IT and RC groups). Assuming 95% confidence and 80% power and an average practice list of 7,500 people, about 2,500 will be aged 40–69 years. Of these around 30% (750) will be at high risk of prevalent undiagnosed diabetes. Given a 70% uptake of screening [53] 525 would be tested and 60 would have prevalent undiagnosed diabetes per practice, of these 42 should complete one year follow-up [54]. The study design exhibits clustering of patients within practices. Typical values of intra-class correlations range from 0.01 to 0.1; we have previously reported correlations of 0.047 for HbA_{1c} and 0.045 for BMI in people with diabetes one year after diagnosis [54]. For clusters of 42 patients the design effect is therefore 3 (range 1.4 to 5.0). Therefore using our previous diabetes cohort data [53,54], (1000 screen-detected cases would allow detection of the following clinically important differences between IT and RC groups: 0.5% in mean HbA_{1c}, (difference between groups at one year in the UKPDS was 0.7% [55]), 11.5 mmHg systolic blood pressure, 1.5 kg/m^2 in body mass index, 10% in the proportion smoking, a 5 point difference in mean EuroQol health utility index [47] and 1.3 in mean anxiety level [49]. These estimations were initially completed for a total of 28 practices in the IT and RC arms. Given the lower than expected prevalence of diabetes within practices (<42 diabetic patients per practice), we recruited more practices, hence reducing the impact of clustering and improving the power of the study. 867 patients diagnosed with diabetes were finally enrolled.

Prior to the development and validation of a CVD risk score incorporating glycaemic control, the original sample size calculation was based on differences in individuals risk factors such as HbA_{1c} and BMI. With the increased number of practices and smaller patients per practice, power was re-assessed using one-year follow-up data using risk factors making up the UKPDS ten-year modelled CVD risk (excluding the unavailable but rare component of atrial fibrillation). This was based on the initial 293 diabetic patients recruited to the RC arm of the study and accounted for clustering (intracluster correlation of 0.0185). It was estimated that there was 90% power at the 5% level of significance to detect a relative effect of 20% in the mean ten-year modelled CVD risk assuming one-year retention of 70% (600 patients in 48 practices).

Discussion

ADDITION-Cambridge is designed to assess the feasibility and cost-effectiveness of a stepwise screening and intensive multi-factorial treatment programme for type 2 diabetes in a defined high-risk group accessible through primary care.

A targeted stepwise approach to screening is supported by the high proportion of undiagnosed diabetes in the UK [53], and the low performance of screening tests as stand alone assessments [56]. *ADDITION-Cambridge* assesses the feasibility of a combination of a diabetes risk score with various biochemical tests as a screening strategy in primary care. Although developed and tested in datasets from population-based surveys [21-23], the performance and yield of this risk score when used as part of a programme in an existing healthcare setting remain uncertain.

The treatment phase of this study has been designed to assess the costs and benefits of early multifactorial therapy in individuals with screen-detected diabetes with the ultimate aim of reducing the risk of cardiovascular events. Trials suggest that intensive treatment of people with type 2 diabetes is beneficial [8,57]. Much of the benefit of early intervention in screen-detected diabetes would depend upon the associated reduction of cardiovascular risk [6]. The treatment algorithm used in *ADDITION-Cambridge* is based on the Steno-2 regimen [8] which was tested in clinically diagnosed patients with diabetes at an advanced stage of the disease. The effectiveness of this regimen in people at an early stage of the disease has yet to be demonstrated. The patient education aspects of the early treatment programme have been informed by reviews on interventions to prevent weight gain [58], educational and psychosocial interventions for adults with diabetes [59], and trials of physical activity promotion [60]. These support the view that an education programme, especially one based on social, behavioural and psychological theory and evidence, can increase the effectiveness of behavioural change strategies [61,62].

For peer review only - http://bmjopen.bmj.com/site/about/guidelines.xhtml

ADDITION-Cambridge will provide evidence about the benefits, harms and costs of implementing a screening and early treatment programme for type 2 diabetes. The results will be of relevance to policy decisions about screening for diabetes, and subsequent management of people early in the course of the disease. Results will also inform approaches to health promotion, the management of chronic disease and risk, and will have implications for the training of practitioners in diabetes care.

Competing interests
The authors declare that they have no competing interest.

Authors' contributions
SJG, NJW and ALK are the principal investigators for the *ADDITION-Cambridge* trial. AT is the trial statistician, KMW and RSB are the trial co-ordinators, JBE, RKS and SJG drafted the manuscript. All authors read and approved the final manuscript. SJG is the paper guarantor

The ADDITION- Cambridge team
Simon J. Griffin, Nicholas J. Wareham, Ann-Louise Kinmonth, Andrew T. Prevost Kate M. Williams, Roslyn S. Barling, Tom Fanshawe, Ryan Butler, Nicola Popplewell, Lincoln A. Sargeant, Paul Roberts, Matt Sims, Fiona Whittle, Julie Grant, James Brimicombe, Wendy Hardeman, Stephen Sutton, Ruhul Amin, Adam Dickinson, Justin B. Echouffo Tcheugui, Rebecca K. Simmons, Francis Finucane, Joana Mitchell, the Field, Data Management, IT and Study Coordination teams of the Medical Research Council Epidemiology Unit. The General Practice and Primary Care Research Unit at the University of Cambridge and the Medical Research Council Epidemiology Unit in Cambridge jointly coordinated the baseline and one-year follow-up phases of the study

Independent Trial Steering Committee in Cambridge
Professors Nigel Stott (Chair), John Weinman, Richard Himsworth, and Paul Little.

Data Monitoring and Ethics Committee
Per Winkel, Jørn Wetterslev and Christian Gluud from The Copenhagen Trial Unit (CTU), Centre for Clinical Intervention Research, Rigshospitalet, Copenhagen University Hospital.

Acknowledgements
The trial is supported by the Wellcome Trust, the Medical Research Council, and National Health Service R&D support funding. ALK, KMW & SJG are members of the National Institute for Health Research (NIHR) School for Primary Care Research. The General Practice and Primary Care Research Unit is supported by NIHR Research funds. SJG receives support from the Department of Health NIHR Programme Grant funding scheme [RP-PG-0606-1259]. The views expressed in this publication are those of the authors and not necessarily those of the Department of Health.

We are grateful to all participants and the practices teams for taking part in the *ADDITION Cambridge trial* – Acorn Community Health Centre, Arbury Road Surgery, Ashwell Surgery, Birchwood Surgery, Breton Medical Practice, Bridge Street Surgery, Brookfields & Cherry Hinton Medical Practice, Broomfields Surgery, Burwell Surgery, Cedar House Surgery, Charles Hicks Medical Centre, Clarkson Surgery, Cornerstone Practice, Cornford House Surgery (Fulbourn and Cherry Hinton Sites), Cottenham Surgery, Cromwell Place Surgery, Dr Smith and Partners Mill Road Surgery, Elsenham Surgery, Freshwell Health Centre, George Clare Surgery, Great Stughton Surgery, John Tasker House, Harston Surgery, Hilton House, Mercheford House, Milton Surgery, Nene Valley Medical Practice, Nevells Road Surgery, New Roysia Surgery, Northcote House Surgery, Nuffield Road Medical Centre, Orchard Surgery, Orchard House Surgery, Orford Lodge Surgery, Orton Medical Practice, Park Medical Centre, Paston Health Centre, Petersfield Medical Practice, Priors Field Surgery, Queen Ediths Medical Practice, Queen Street Surgery-Whittlesey, South Street Surgery, Regal Chambers Surgery, Rainbow Surgery, Riverside Practice, Roman Gate Surgery, Rosalind Franklin House, The Health Centre (274 Great North Road, Eaton Socon), The Health Centre (Heath Road, Woolpit, Bury St Edmunds), The Health Centre (Townley Close, Upwell), The Old Exchange Surgery, The Surgery (25 Alms Hill, Bourn, Cambridge), The Surgery (Cathedral View, East street, Manea), The Surgery (Chequers Lane, Papworth Everard, Huntingdon), The Surgery (Glapthorne Road, Oundle, Peterborough), The Surgery (Mayfield, Buckden), The Surgery(Margaret Street, Thaxted, Essex), The Surgery Stanground, The Surgery (The Green, Haddenham, Ely), The Surgery (Tunbridge Lane, Bottisham), Trumpington Street Medical Practice, Wellside Surgery, York Street Medical Practice,.

We thank the CUHNFT Department of Clinical Biochemistry and the NIHR Cambridge Biomedical Research Centre, Core Biochemical Assay Laboratory for carrying out the biochemical assays.

References
1. Wild S, Roglic G, Green A, Sicree R, King H: **Global prevalence of diabetes: estimates for the year 2000 and projections for 2030.** *Diabetes Care* 2004, **27(5):**1047-1053.
2. Institute KsFP: **Counting the cost: the real impact of non-insulin-dependent diabetes mellitus.** London: British Diabetic Association; 1997.
3. Harris MI: **Undiagnosed NIDDM: clinical and public health issues.** *Diabetes Care* 1993, **16(4):**642-652.
4. **UK Prospective Diabetes Study 6. Complications in newly diagnosed type 2 diabetic patients and their association with different clinical and biochemical risk factors.** *Diabetes Res* 1990, **13(1):**1-11.
5. Harris MI, Klein R, Welborn TA, Knuiman MW: **Onset of NIDDM occurs at least 4–7 yr before clinical diagnosis.** *Diabetes Care* 1992, **15(7):**815-819.
6. Wareham NJ, Griffin SJ: **Should we screen for type 2 diabetes? Evaluation against National Screening Committee criteria.** *BMJ* 2001, **322(7292):**986-988.
7. Glumer C, Yuyun M, Griffin S, Farewell D, Spiegelhalter D, Kinmonth AL, Wareham NJ: **What determines the cost-effectiveness of diabetes screening?** *Diabetologia* 2006, **49(7):**1536-1544.
8. Gaede P, Vedel P, Larsen N, Jensen GV, Parving HH, Pedersen O: **Multifactorial intervention and cardiovascular disease in patients with type 2 diabetes.** *N Engl J Med* 2003, **348(5):**383-393.
9. Gaede P, Valentine WJ, Palmer AJ, Tucker DM, Lammert M, Parving HH, Pedersen O: **Cost-effectiveness of intensified versus conventional multifactorial intervention in type 2 diabetes: results and projections from the Steno-2 study.** *Diabetes Care* 2008, **31(8):**1510-1515.
10. **Tight blood pressure control and risk of macrovascular and microvascular complications in type 2 diabetes: UKPDS 38. UK Prospective Diabetes Study Group.** *BMJ* 1998, **317(7160):**703-713.
11. Colhoun HM, Betteridge DJ, Durrington PN, Hitman GA, Neil HA, Livingstone SJ, Thomason MJ, Mackness MI, Charlton-Menys V, Fuller

For peer review only - http://bmjopen.bmj.com/site/about/guidelines.xhtml

JH: **Primary prevention of cardiovascular disease with atorvastatin in type 2 diabetes in the Collaborative Atorvastatin Diabetes Study (CARDS): multicentre randomised placebo-controlled trial.** *Lancet* 2004, **364(9435):**685-696.

12. Collins R, Armitage J, Parish S, Sleigh P, Peto R: **MRC/BHF Heart Protection Study of cholesterol-lowering with simvastatin in 5963 people with diabetes: a randomised placebo-controlled trial.** *Lancet* 2003, **361(9374):**2005-2016.
13. **Effects of ramipril on cardiovascular and microvascular outcomes in people with diabetes mellitus: results of the HOPE study and MICRO-HOPE substudy. Heart Outcomes Prevention Evaluation Study Investigators.** *Lancet* 2000, **355(9200):**253-259.
14. Holman RR, Paul SK, Bethel MA, Neil HA, Matthews DR: **Long-term follow-up after tight control of blood pressure in type 2 diabetes.** *N Engl J Med* 2008, **359(15):**1565-1576.
15. Gerstein HC, Miller ME, Byington RP, Goff DC Jr, Bigger JT, Buse JB, Cushman WC, Genuth S, Ismail-Beigi F, Grimm RH Jr, *et al.*: **Effects of intensive glucose lowering in type 2 diabetes.** *N Engl J Med* 2008, **358(24):**2545-2559.
16. Patel A, MacMahon S, Chalmers J, Neal B, Billot L, Woodward M, Marre M, Cooper M, Glasziou P, Grobbee D, *et al.*: **Intensive blood glucose control and vascular outcomes in patients with type 2 diabetes.** *N Engl J Med* 2008, **358(24):**2560-2572.
17. Duckworth W, Abraira C, Moritz T, Reda D, Emanuele N, Reaven PD, Zieve FJ, Marks J, Davis SN, Hayward R, *et al.*: **Glucose control and vascular complications in veterans with type 2 diabetes.** *N Engl J Med* 2009, **360(2):**129-139.
18. Holman RR, Paul SK, Bethel MA, Matthews DR, Neil HA: **10-year follow-up of intensive glucose control in type 2 diabetes.** *N Engl J Med* 2008, **359(15):**1577-1589.
19. **Multicentre aneurysm screening study (MASS): cost effectiveness analysis of screening for abdominal aortic aneurysms based on four year results from randomised controlled trial.** *BMJ* 2002, **325(7373):**1135.
20. Lauritzen T, Griffin S, Borch-Johnsen K, Wareham NJ, Wolffenbuttel BH, Rutten G: **The ADDITION study: proposed trial of the cost-effectiveness of an intensive multifactorial intervention on morbidity and mortality among people with Type 2 diabetes detected by screening.** *Int J Obes Relat Metab Disord* 2000, **24(Suppl 3):**S6-11.
21. Griffin SJ, Little PS, Hales CN, Kinmonth AL, Wareham NJ: **Diabetes risk score: towards earlier detection of type 2 diabetes in general practice.** *Diabetes Metab Res Rev* 2000, **16(3):**164-171.
22. Park PJ, Griffin SJ, Sargeant L, Wareham NJ: **The performance of a risk score in predicting undiagnosed hyperglycemia.** *Diabetes Care* 2002, **25(6):**984-988.
23. Spijkerman AM, Yuyun MF, Griffin SJ, Dekker JM, Nijpels G, Wareham NJ: **The performance of a risk score as a screening test for undiagnosed hyperglycemia in ethnic minority groups: data from the 1999 health survey for England.** *Diabetes Care* 2004, **27(1):**116-122.
24. Haffner SM, Stern MP, Hazuda HP, Mitchell BD, Patterson JK: **Cardiovascular risk factors in confirmed prediabetic individuals. Does the clock for coronary heart disease start ticking before the onset of clinical diabetes?** *JAMA* 1990, **263(21):**2893-2898.
25. Spijkerman AM, Adriaanse MC, Dekker JM, Nijpels G, Stehouwer CD, Bouter LM, Heine RJ: **Diabetic patients detected by population-based stepwise screening already have a diabetic cardiovascular risk profile.** *Diabetes Care* 2002, **25(10):**1784-1789.
26. Van Drenth BB, Hulscher MEJL, Wouden JC Van der, Mokkink HGA, Van Weel C, Grol RPTM: **Relationship between practice organization and cardiovascular risk factor recording in general practice.** *Br J Gen Pract* 1998, **48:**1054-1058.
27. Marteau TM: **Psychological costs of screening.** *BMJ* 1989, **299(6698):**527.
28. Park P, Simmons RK, Prevost AT, Griffin SJ: **Screening for type 2 diabetes is feasible, acceptable, but associated with increased short-term anxiety: a randomised controlled trial in British general practice.** *BMC Public Health* 2008, **8(1):**350.
29. Adriaanse MC, Snoek FJ: **The psychological impact of screening for type 2 diabetes.** *Diabetes Metab Res Rev* 2006, **22(1):**20-25.
30. Eborall HC, Griffin SJ, Prevost AT, Kinmonth AL, French DP, Sutton S: **Psychological impact of screening for type 2 diabetes: controlled trial and comparative study embedded in the ADDITION (Cambridge) randomised controlled trial.** *BMJ* 2007, **335(7618):**486.
31. Narayan KM, Boyle JP, Thompson TJ, Sorensen SW, Williamson DF: **Lifetime risk for diabetes mellitus in the United States.** *JAMA* 2003, **290(14):**1884-1890.
32. Kuo HS, Chang HJ, Chou P, Teng L, Chen TH: **A Markov chain model to assess the efficacy of screening for non-insulin dependent diabetes mellitus (NIDDM).** *Int J Epidemiol* 1999, **28(2):**233-240.
33. Chang HJ, Kuo HS, Tung TH, Chou P, Chen TH: **Evaluation of a population-based screening for type 2 diabetes: a community-based screening project in Puli, Taiwan.** *Prev Med* 2000, **31(4):**396-402.
34. Engelgau MM, Narayan KM, Herman WH: **Screening for type 2 diabetes.** *Diabetes Care* 2000, **23(10):**1563-1580.
35. Bateman H: **A Research Information Sheet for Practices (RISP): a tool to facilitate research participation.** *Fam Pract* 2002, **19(6):**691-697.
36. Alberti KG, Zimmet PZ: **Definition, diagnosis and classification of diabetes mellitus and its complications;Part 1: diagnosis and classification of diabetes mellitus.** Geneva, Switzerland: World Health Organisation; 1999.
37. Leventhal H, Safer MA, Panagis DM: **The impact of communications on the self-regulation of health beliefs, decisions, and behavior.** *Health Educ Q* 1983, **10(1):**3-29.
38. **Diabetes for beginners- Type2** [http://www.diabetes.org.uk]
39. Coleman RL, Stevens RJ, Holman RR: **Estimating cardiovascular disease risk for individuals with Type 2 diabetes: the new UKPDS Risk Engine [abstract].** *Diabet Med* 2007, **24(Suppl 1):**56.
40. Horne R, Weinman J: **Predicting treatment adherence: an overview of theoretical models.** In *Adherence to treatment in medical conditions* Edited by: Myers L, Midence K. London Harwood Academic; 1998:25-50.
41. Wareham NJ, Rennie KL: **The assessment of physical activity in individuals and populations: why try to be more precise about how physical activity is assessed?** *Int J Obes Relat Metab Disord* 1998, **22(Suppl 2):**S30-38.
42. Craig CL, Marshall AL, Sjostrom M, Bauman AE, Booth ML, Ainsworth BE, Pratt M, Ekelund U, Yngve A, Sallis JF, *et al.*: **International physical activity questionnaire: 12-country reliability and validity.** *Med Sci Sports Exerc* 2003, **35(8):**1381-1395.
43. Bingham SA, Gill C, Welch A, Cassidy A, Runswick SA, Oakes S, Lubin R, Thurnham DI, Key TJ, Roe L, *et al.*: **Validation of dietary assessment methods in the UK arm of EPIC using weighed records, and 24-hour urinary nitrogen and potassium and serum vitamin C and carotenoids as biomarkers.** *Int J Epidemiol* 1997, **26(Suppl 1):**S137-151.
44. Bradley C: *Handbook of psychology and diabetes: a guide to psychological measurement in diabetes research and practice* Harwood Academic Press; 1994.
45. Ware J, Snow KK, Kosinski M, Gandek B: *SF-36 Health Survey. Manual & Interpretation Guide* Boston, Massachusetts: Nimrod press; 1993.
46. Ware JE, Kosinski M, Dewey JE, Gandek B: *How to score and interpret Single-Item Health Status Measures: A Manual for Users of the SF-8™ Health Survey* Lincoln: RI:QualityMetric Incorporated; 2001.
47. Kind P, Dolan P, Gudex C, Williams A: **Variations in population health status: results from a United Kingdom national questionnaire survey.** *BMJ* 1998, **316(7133):**736-741.
48. Mercer SW, Maxwell M, Heaney D, Watt GC: **The consultation and relational empathy (CARE) measure: development and preliminary validation and reliability of an empathy-based consultation process measure.** *Fam Pract* 2004, **21(6):**699-705.
49. Marteau TM, Bekker H: **The development of a six-item short-form of the state scale of the Spielberger State-Trait Anxiety Inventory (STAI).** *Br J Clin Psychol* 1992, **31(Pt 3):**301-306.
50. Knapp M, Beecham J: **Reduced list costings: examination of an informed short cut in mental health research.** *Health Econ* 1993, **2(4):**313-322.
51. Rose GA: **The diagnosis of ischaemic heart pain and intermittent claudication in field surveys.** *Bull World Health Organ* 1962, **27:**645-658.
52. Feldman EL, Stevens MJ, Thomas PK, Brown MB, Canal N, Greene DA: **A practical two-step quantitative clinical and electrophysiological assessment for the diagnosis and staging of diabetic neuropathy.** *Diabetes Care* 1994, **17(11):**1281-1289.

For peer review only - http://bmjopen.bmj.com/site/about/guidelines.xhtml

53. Williams DR, Wareham NJ, Brown DC, Byrne CD, Clark PM, Cox BD, Cox LJ, Day NE, Hales CN, Palmer CR, *et al.*: **Undiagnosed glucose intolerance in the community: the Isle of Ely Diabetes Project.** *Diabet Med* 1995, **12(1):**30-35.
54. Kinmonth AL, Woodcock A, Griffin S, Spiegal N, Campbell MJ: **Randomised controlled trial of patient centred care of diabetes in general practice: impact on current wellbeing and future disease risk. The Diabetes Care From Diagnosis Research Team.** *BMJ* 1998, **317(7167):**1202-1208.
55. **U.K. prospective diabetes study 16. Overview of 6 years' therapy of type II diabetes: a progressive disease. U.K. Prospective Diabetes Study Group.** *Diabetes* 1995, **44(11):**1249-1258.
56. Waugh N, Scotland G, McNamee P, Gillett M, Brennan A, Goyder E, Williams R, John A: **Screening for type 2 diabetes: literature review and economic modelling.** *Health Technol Assess* 2007, **11(17):**iii-iv, ix-xi-1-125.
57. **Intensive blood-glucose control with sulphonylureas or insulin compared with conventional treatment and risk of complications in patients with type 2 diabetes (UKPDS 33). UK Prospective Diabetes Study (UKPDS) Group.** *Lancet* 1998, **352(9131):**837-853.
58. Hardeman W, Griffin S, Johnston M, Kinmonth AL, Wareham NJ: **Interventions to prevent weight gain: a systematic review of psychological models and behaviour change methods.** *Int J Obes Relat Metab Disord* 2000, **24(2):**131-143.
59. Griffin SJ, Kinmonth AL, Skinner C, Kelly J: **Educational and psychosocial interventions for adults with diabetes.** London: British Diabetic Association; 1999.
60. Hillsdon M, Thorogood M, Anstiss T, Morris J: **Randomised controlled trials of physical activity promotion in free living populations: a review.** *J Epidemiol Community Health* 1995, **49(5):**448-453.
61. Padgett D, Mumford E, Hynes M, Carter R: **Meta-analysis of the effects of educational and psychosocial interventions on management of diabetes mellitus.** *J Clin Epidemiol* 1988, **41(10):**1007-1030.
62. Hardeman W, Johnston M, Kinmonth AL, Wareham NJ: **The use of the Theory of Planned Behaviour in interventions: a systematic review.** *Proceedings of the British Psychological Society* 1999, **7(1):**31.

Pre-publication history

The pre-publication history for this paper can be accessed here:

http://www.biomedcentral.com/1471-2458/9/136/prepub

Publish with **BioMed Central** and every scientist can read your work free of charge

"BioMed Central will be the most significant development for disseminating the results of biomedical research in our lifetime."

Sir Paul Nurse, Cancer Research UK

Your research papers will be:

- available free of charge to the entire biomedical community
- peer reviewed and published immediately upon acceptance
- cited in PubMed and archived on PubMed Central
- yours — you keep the copyright

Submit your manuscript here:
http://www.biomedcentral.com/info/publishing_adv.asp

BioMedcentral

For peer review only

BMJ Open

How good are GPs at adhering to a pragmatic trial protocol in primary care? Results from the ADDITION-Cambridge cluster-randomized pragmatic trial

Journal:	*BMJ Open*
Manuscript ID	bmjopen-2016-015295.R1
Article Type:	Research
Date Submitted by the Author:	28-Apr-2017
Complete List of Authors:	Laxy, Michael; Helmholtz Zentrum München, Institute of Health Economics and Health Care Management<br>Wilson, Edward; University of Cambridge, Cambridge Centre for Health Services Research<br>Boothby, Clare; Institute of Metabolic Science, MRC Epidemiology Unit<br>Griffin, Simon; Institute of Metabolic Science, MRC Epidemiology Unit
**Primary Subject Heading**:	Health services research
Secondary Subject Heading:	Diabetes and endocrinology
Keywords:	diabetes mellitus, pragmatic trial, protocol adherence, PRIMARY CARE

SCHOLARONE™ Manuscripts

For peer review only

How good are GPs at adhering to a pragmatic trial protocol in primary care? Results from the ADDITION-Cambridge cluster-randomized pragmatic trial

Authors: Michael Laxy[1,2,3], Edward C.F. Wilson[4], Clare E. Boothby[3], Simon J. Griffin[3]

[1] Institute of Health Economics, Helmholtz Zentrum München, Neuherberg, Germany, Ingolstädter Landstraße 1, 85764 München

[2] German Center for Diabetes Research, Neuherberg, Germany, Ingolstädter Landstraße 1, 85764 München

[3] MRC Epidemiology Unit, University of Cambridge, Cambridge, UK, Cambridge Biomedical Campus, Cambridge CB2 0SL

[4] Cambridge Centre for Health Services Research, University of Cambridge, Cambridge, UK, Forvie Site Cambridge Biomedical Campus, Cambridge CB2 0SR

Email addresses:

Michael Laxy: michael.laxy@helmholtz-muenchen.de

Ed Wilson: ed.wilson@medschl.cam.ac.uk

Clare Boothby: Clare.Boothby@mrc-epid.cam.ac.uk

Simon Griffin: sjg49@medschl.cam.ac.uk

Corresponding author: Dr. Ed Wilson, Cambridge Centre for Health Services Research, University of Cambridge, Institute of Public Health, Forvie Site, Robinson Way, Cambridge CB2 0SR; Email: ed.wilson@medschl.cam.ac.uk

Manuscript word count: 5,070

Key words: diabetes mellitus, pragmatic trial, protocol adherence, primary care

For peer review only - http://bmjopen.bmj.com/site/about/guidelines.xhtml

Abstract

Objective: To assess the fidelity of general practitioners' (GP) adherence to a long term pragmatic trial protocol.

Design: Retrospective analyses of electronic primary care records of participants in the pragmatic cluster-randomised ADDITION (Anglo-Danish-Dutch Study of Intensive Treatment In People with Screen Detected Diabetes in Primary Care)-Cambridge trial, comparing intensive multi-factorial treatment (IT) vs. routine care (RC). Data were collected from the date of diagnosis until December 2010.

Setting: Primary care surgeries in the East of England

Study sample/participants: A subsample (n=189, RC-arm: n=99, IT-arm: n=90) of patients from the ADDITION-Cambridge cohort (867 patients), consisting of 40-69 year old patients with screen detected diabetes mellitus.

Interventions: In the RC-arm treatment was delivered according to concurrent treatment guidelines. Surgeries in the IT-arm received funding for additional contacts between GPs/nurses and patients, and GPs were advised to follow more intensive treatment algorithms for the management of glucose, lipids and blood pressure and aspirin therapy than in the RC-arm.

Outcome measures: The number of annual contacts between patients and GPs/nurses, the proportion of patients receiving prescriptions for cardio-metabolic medication in years 1 to 5 after diabetes diagnosis, and the adherence to prescription algorithms.

Results: The difference in the number of annual GP contacts (β=0.65) and nurse contacts (β=-0.15) between the study arms was small and insignificant. Patients in the IT-arm were more likely to receive glucose-lowering (OR=3.27), ACE-inhibiting (OR=2.03) and lipid-lowering drugs (OR=2.42, all p-values<0.01) than patients in the RC-arm. The prescription adherence varied between medication classes, but improved in both trial arms over the 5 year follow-up.

For peer review only - http://bmjopen.bmj.com/site/about/guidelines.xhtml

Conclusions: The adherence of GPs to different aspects of the trial protocol was mixed. Background changes in health care policy need to be considered as they have the potential to dilute differences in treatment intensity and hence incremental effect.

Clinical trial number: ISRCTN86769081

Article Summary: Strengths and Limitations of the Study

- Pragmatic trials aim to produce externally valid results for decision makers. If and to what extent pragmatic trial interventions are delivered to patients often remains unknown.
- This study describes the adherence of GPs to the ADDITION trial protocol and hence provides a unique insight about what we can expect in future long-term pragmatic studies in the primary care context, particularly in the context of policy and guideline changes.
- Analyses are based on a subsample of participants of the ADDITION-Cambridge trial conducted in the East of England. Therefore, the generalizability of results might be limited.

For peer review only

For peer review only - http://bmjopen.bmj.com/site/about/guidelines.xhtml

Background

Type 2 diabetes is an increasing public health problem associated with premature mortality and costly micro- and macro-vascular complications in terms of both reduced quality of life and financial burden, causing substantial economic pressure on healthcare systems and societies [1-4].

Previous research has shown that intensive treatment of cardiovascular risk factors is an effective and cost-effective intervention for patients with longstanding diabetes or routinely diagnosed diabetes [5-8]. In contrast, little was known about the cost-effectiveness of intensive primary care based treatment in patients in the early stages of the disease, such as screen detected populations. The pragmatic cluster-randomised ADDITION (**A**nglo-**D**anish-**D**utch Study of **I**ntensive **T**reatment **I**n People with Scree**n** Detected Diabetes in Primary Care) - trial (ISRCTN86769081) was one of the first studies addressing this important question [9-11]. Results showed that, compared to routine care, early intensive treatment modestly improved levels of cardiovascular risk factors, but did not significantly reduce the incidence of cardiovascular events, microvascular complications, and cardiovascular/overall mortality over the 5 year study period [12-14].

Pragmatic trials aiming to generate externally valid evidence in a real world setting, such as ADDITION, always present uncertainties concerning the implementation of the planned interventions in daily practice. Unlike highly controlled efficacy trials in which compliance to a (simple, one-dimensional) intervention can (and must) be assured, the purpose of pragmatic trials is to assess the effectiveness of a (complex, multifactorial) intervention in routine settings. In the ADDITION-Cambridge trial, intensive treatment (IT) was compared to routine care (RC) for screen detected diabetes patients. IT in ADDITION was a multifactorial intervention including treatment targets and treatment algorithms that were more intensive than those in contemporary UK national treatment guidelines, as well as educational material for patients [10; 15-17]. However, the degree to which protocol components were implemented into practice, and hence the degree to which more intensified treatment was actually provided to patients in the intervention arm, has remained unknown. Furthermore, potential changes in national treatment guidelines towards more intensive care, and the

For peer review only

For peer review only - http://bmjopen.bmj.com/site/about/guidelines.xhtml

introduction of the pay for performance system in England within the national Quality and Outcomes Framework (QOF) [18; 19], are likely to have improved routine care and may have diluted the difference in treatment intensity between the study arms over time [20].

Beyond improving understanding of the results of the ADDITION-Cambridge study, knowledge about whether and how the intervention was actually delivered in practice can inform future pragmatic trials in relation to barriers to protocol adherence, and the difference in treatment intensity that can be expected in a primary care based pragmatic trial in the context of background policy changes.

The objective of this study was therefore to describe the adherence of GPs to the trial protocol and to compare the intensity of care delivered to screen detected diabetes patients between the trial arms.

Methods

Study design

The ADDITION-Cambridge study protocol has been published elsewhere[10]. In brief, ADDITION-Cambridge is part of the ADDITION-Europe trial, which consisted of two phases: a screening program and a pragmatic, cluster-randomised trial comparing the effect of early intensive treatment versus routine care on five year cardiovascular risk in patients with screen-detected type 2 diabetes mellitus [9]. The primary endpoint was a composite of cardiovascular morbidity and mortality (cardiovascular death, non-fatal myocardial infarction, non-fatal stroke, non-traumatic amputations and revascularisations).

Study population

For ADDITION-Cambridge, 33,539 eligible individuals were invited to stepwise screening. Individuals eligible for screening were people registered at one of the participating general surgeries around Cambridge, aged 40 to 69 years, not known to have diabetes and with a diabetes risk score of >0.17 (corresponding to the top 25% of the population distribution). The risk score included age, sex, BMI, steroid and antihypertensive medication as well as smoking and family history [21]. Exclusion

For peer review only - http://bmjopen.bmj.com/site/about/guidelines.xhtml

criteria were assessed by the potential participant's GP. Patients with severe illness with a life expectancy of less than 12 months, those with psychological or psychiatric disorders that might invalidate informed consent and those who were housebound, pregnant or breast feeding were excluded from the study. 867 eligible patients (from n=49 surgeries) with screen detected diabetes participated in the pragmatic primary care based intervention trial. Ethical approval was granted by the Eastern Multi-Regional Ethics Committee (ref 02/5/54). Written informed consent was obtained from all participants. This trial is registered as ISRCTN86769081.

Due to the high expenses of assessing and extracting data from electronic primary care records it was decided in the planning phase of the ADDITION Cambridge study that only the records of a subset of the study will be assessed. It was decided that the records of participants with a primary endpoint within the 5 years of follow up plus the records of two random participants without a primary endpoint from the same GP surgery will be accessed. Consequently, the records of 63 participants with a primary endpoint (30 from the IT arm and 33 from the RC arm) and of 126 participants without a primary endpoint (60 from the IT arm and 66 from the RC arm) were collected. This selection procedure led in total to a subsample of 189 participants (IT: n=90 patients, RC: n=99 patients) from 34 surgeries (IT: 18 GP surgeries, RC: 16 GP surgeries). The study design is illustrated in detail in **figure 1**.

Intensive Treatment and Routine Care

Patients were treated according to the treatment allocation of their surgery. In the RC-arm patients received diabetes care through the National Health Service according to current UK guidelines and recommendations [15-17]. In the IT-arm additional features were added to current RC:

a) Surgeries received funding for 3 additional 10-minute GP consultations and 3 additional nurse consultations per year in the first 3 years after diagnosis.

b) Treatment algorithms were introduced along with underlying evidence demonstrating positive effects on CVD risk factors among patients with type 2 diabetes. In the IT-arm therapy with glucose lowering medication was indicated if $HbA_{1c} \geq 6.5\%$; ACE inhibitors/ARBs if BP $\geq$ 120/80mmHg;

For peer review only - http://bmjopen.bmj.com/site/about/guidelines.xhtml

statins if cholesterol ≥ 3.5 mmol/l; and aspirin for all patients independent of their risk factor levels (assuming that patients had no contraindications). The thresholds for treatment initiation for glucose lowering, BP lowering and lipid lowering medication and for aspirin therapy in both the IT-arm (based on the trial protocol [10]) and the RC-arm (based on national guidelines [15-17]) are summarized in **Table 1**.

Table 1:
Criteria for the initiation of glucose lowering, blood pressure lowering, lipid lowering and platelet inhibiting (aspirin) medication according to the trial protocol (IT-arm) and national guidelines (RC-arm) ɫ

	Glucose-lowering therapy	Blood pressure-lowering therapy	Lipid-lowering therapy	CVD risk-lowering aspirin therapy
**Routine Care (RC)**	- if HbA_{1c} ≥ 7% ⊦	- if BP ≥ 160/100<br>- if 140/80 mmHg ≤ BP < 160/100 mmHg and either prevalent CVD or 10-year CHD risk ≥ 15%<br>*(ACE inhibitors, ARBs, B-blockers or diuretics as first choice)*	- if total cholesterol ≥ 5 mmol/l or triglycerides ≥ 2.3mmol/l<br>- if prevalent CVD or 10-year CHD-risk ≥ 15%	- if prevalent CVD or 10-year CHD-risk ≥ 15%
**Intensive Treatment (IT)**	- if HbA_{1c} ≥ 6.5%	if ≥ 120/80 mmHg or prevalent CVD<br>*(ACE inhibitors/ARBs as first choice)*	- if total cholesterol ≥ 3.5mmol/l	- independent of risk profile

ɫ Criteria are based on the national treatment guidelines from 2002 [15-17] and the ADDITION trial protocol [10]
⊦ a range of 6.5% - 7.5% was mentioned. Consequently, the arithmetic mean of the borders (7%) was used as threshold

This figure does not claim to comprehensively describe the national treatment algorithms from the year 2002 or the detailed ADDITION trial protocol. It only highlights the differences in criteria for the initiation of drug therapy between IT and RC and does not account for possible contraindications.

c) Practice teams received theory-based educational materials to hand over to the patients, aiming to provide a shared framework for the management of their disease. Furthermore, GPs were advised to refer patients to a dietician and patients were encouraged through their GPs and nurses to increase their physical activity, to avoid excessive alcohol intake, to lose weight, to stop smoking, to adhere to medication, and to self-monitor blood glucose if given a glucometer by their GP.

Intensive treatment was promoted to participating surgeries by practice-based educational meetings with GPs and nurses. This included initial practice-based academic detailing conducted by a diabetologist and an academic GP to introduce treatment algorithms, and two interactive practice-based feedback sessions (approximately 6 and 14 months after the initial education session) to support and monitor treatment delivery.

For peer review only - http://bmjopen.bmj.com/site/about/guidelines.xhtml

Measures of treatment intensity

Information on the intensity of delivered care was extracted from the electronic primary care records of participating patients from the date of the diabetes diagnosis until December 2010 by a researcher blind to the GP surgery study group allocation. These files recorded the date and type of delivered services, including consultations with primary care health professionals, prescribed medications and laboratory measurements/tests. For the analyzed trial population more than 80,000 observations were available in the first 5 years after diagnosis. Clear text functions were used and algorithms were derived to classify the obtained information. Ambiguous observations were screened and coded by hand. Anatomic Therapeutic Chemical (ATC) codes were assigned to drugs to categorize medication classes. The intensity of care indicators were defined as follows:

Contact with health care professionals: The annual number of contacts between patients and GPs (including GP partners, GP principals, GP associates, out-of-hours doctors) and nurses (including practice nurses, nurse practitioners and nurse specialists). This included all contacts as we were unable to distinguish those related to diabetes alone.

Medication: Continuous treatment (≥ 4 prescriptions annually) with glucose lowering drugs (metformin, sulphonylurea, thiazolidinedione, insulin, other glucose lowering drugs), ACE inhibiting drugs (ACE inhibitors or ARBs), lipid lowering drugs (statins, other cholesterol lowering drugs) or aspirin.

Monitoring of risk factor levels: Regular monitoring of glycaemic control (≥ 2 HbA_{1c} tests per year), lipid profile (≥ 1 cholesterol test per year) and kidney function (≥ 1 urine albumin-creatinine ratio (UACR) test per year) [15-17].

Statistical Analyses

We analysed the difference in treatment intensity within the first 5 years from date of diagnosis. The study period was subdivided into five annual intervals representing year 1 (day 1 – day 365) to year 5 (day 1460 – day 1825) from diagnosis. 16 patients whose electronic primary care records did not contain information for at least one entire year were excluded from the analysis, resulting in an

For peer review only - http://bmjopen.bmj.com/site/about/guidelines.xhtml

analysis sample of 173 patients from 34 GP surgeries with a mean cluster size of 5 patients (IT: 82 patients from 18 surgeries, RC: 91 patients from 16 surgeries). Due to non-availability of data, surgery changes and deaths the total number of complete observed patient-years over the follow up period was 827 for contact with health care professionals and monitoring and 737 for prescriptions.

We applied linear regression models separately for years 1 to 5 in order to analyse the difference in the number of contacts with GPs and nurses for each individual year. A multi-level linear regression model accounting for repeated observations (year 1-5) within patients was applied to test the overall difference in the number of annual contacts between the study arms over the 5 year study period. This model included an interaction term between the year since diagnosis and the treatment to capture any time – treatment interactions.

In parallel with the linear regression models for the frequency of contacts with health care professionals, logistic regression models were applied to assess the likelihood of receiving continuous medication (≥ 4 prescriptions annually). In a secondary analysis, we also examined the likelihood of receiving regular monitoring of glycaemic control, lipid profile and kidney function and the likelihood of seeing a dietician[15-17].

Linear and logistic regression models were adjusted for age and sex and accounted for patients being clustered into surgeries (2-level model for stratified analyses and 3-level models for overall analyses). As the non-random selection of the analysed subsample does not exactly represent the study population, we tested in a sensitivity analysis if the introduction of a weighting factor (inverse probability of being included in the study based on the status of having a primary endpoint) has an impact on the results. We also altered the thresholds for the definition of 'continuous' medication (from 4 to 2, 6 and 12 prescriptions) to assess the sensitivity towards these threshold definitions. To assess the sensitivity to missing data we further refitted the analyses to a regression-based multiple-imputed (n=10 imputations) dataset (n=189 patients). Statistical analyses were performed with SAS 9.3 using the GLIMMIX, MI and MIANALYZE procedures (Cary, NC).

For peer review only - http://bmjopen.bmj.com/site/about/guidelines.xhtml

To gain a more detailed insight into the pattern of GPs' adherence to treatment algorithms, we further extracted clinical information including HbA_{1c}, BP, cholesterol, triglycerides, prevalent CVD (defined as MI or stroke) and 10-year modelled CHD risk (using the UKPDS risk engine V2) from the baseline, year 1 and year 5 examinations of the ADDITION study. Missing clinical values were imputed by the methods of last observation carried forward (LOCF) and first observation carried backwards (FOCB) to avoid shrinkage of the sample size. We calculated the proportion of patients who should have received medication, i.e. the proportion of patients whose clinical values exceeded the thresholds referred to in the trial protocol [10] and the national guidelines[15-17] (P [clinical value ≥ threshold]) and the proportion of patients who actually received at least one prescription in a time frame of 3 months after the date of the laboratory measurement (P [# of prescriptions ≥ 1]) (Table 1). We finally defined the adherence of GPs to the trial protocol/national guidelines descriptively as the proportion of patients who receive at least one prescription, out of those patients whose clinical values exceed the thresholds (P [# of prescriptions ≥ 1] | [clinical value ≥ threshold]).

Results

Baseline sample characteristics

Characteristics of the sample at baseline are shown in **Table 2**. The mean age of the sample was 62 years, 34% were female and 96% Caucasian. The biomedical characteristics of the comparison arms were balanced. No differences were observed between the full sample (n=189) and the analysis sample (n=173).

Table 2:
Baseline characteristics of the used subsample of ADDITION Cambridge

	Intensive Treatment	Routine Care
N	82	91
Female sex, n (%)	30 (36.6)	30 (30.3)
Caucasian ethnicity, n (%)	77 (93.9)	96 (97)
Age, mean (SD)	61.87 (7.28)	62.01 (6.81)
BMI [kg/m2], mean (SD)	33.6 (5.6)	33.8 (5.9)
Total cholesterol [mmol/L], mean (SD)	5.47 (1.12)	5.46 (1.22)
HDL cholesterol [mmol/L], mean (SD)	1.16 (0.32)	1.2 (0.31)
Systolic blood pressure [mm Hg], mean (SD)	143 (20.8)	143.8 (22.2)
HbA_{1c} [%], mean (SD)	7.84 (2.09)	7.27 (1.59)

For peer review only - http://bmjopen.bmj.com/site/about/guidelines.xhtml

SD: Standard Deviation, BMI: Body Mass Index, HDL: High Density Lipoprotein, HbA_{1c}: glycated haemoglobin; N: number of individuals included in the analysis sample

Contact with health care professionals

The adjusted mean number of annual GP and nurse contacts is graphically illustrated in **Figure 2**. We found no difference in the mean annual number of contacts with GPs (IT: 5.80, vs. RC: 5.15, β=0.65 [95%-CI: -0.95, +2.26.13] or nurses (IT: 5.34 vs. RC: 5.49, β = -0.15 [-1.77, +1.48]). In addition, no consistent increase or decrease in the number of GP or nurse consultations over time could be observed.

Medication

The proportion of GPs who regularly prescribed (≥ 4 times annually) glucose lowering and cardio-protective drugs and odds ratios for the likelihood of regular prescriptions are shown in **Figure 3**.

GPs in the IT-arm were 3.27 [95%CI: 1.81 to 5.93] times more likely to regularly prescribe glucose lowering medications compared to GPs in the RC-arm. However, this difference diminished over the follow-up period as more patients in the RC arm were also prescribed medication. Patients in the IT-arm also had a greater chance of being prescribed lipid lowering medication (OR=2.42 [1.30 to 4.51]) and ACE inhibiting drugs (OR=2.03 [1.13, 3.65]), which were, in contrast to routine care guidelines, the first choice BP lowering drug according to the trial protocol. But no significant difference was observed between the trial arms for the category of BP lowering drugs as a whole (including beta-blocker, diuretics etc.) (OR=1.41 [0.71, 2.80]) (**Appendix 1**). No significant difference was observed between the trial arms for prescription of aspirin. Overall in both treatment arms, the likelihood of patients receiving glucose lowering, ACE inhibiting and lipid-lowering medications increased from diagnosis to five year follow up.

Monitoring of risk factors

The proportion of patients receiving regular HbA_{1c} tests (≥ 2 annually, 45% of patients), lipid tests (≥ 1 annually, 55% of patients) and UACR tests (≥ 1 annually, 75% of patients) was low. No significant

For peer review only - http://bmjopen.bmj.com/site/about/guidelines.xhtml

difference was observed between the treatment arms (HbA_{1c} tests: OR=1.56 [0.63, 3.83], lipid tests OR=1.53 [0.51, 4.60], UACR-test: OR=0.82 [0.34, 1.98]) (**Appendix 1**).

Sensitivity Analysis

Analyses of multiple-imputed datasets led to qualitatively and quantitatively similar results. Also the introduction of a weighting factor to account for non-random patient selection yielded comparable results. Using different thresholds for the definition of 'continuous medication' showed that the results for glucose and lipid lowering medications were not sensitive to threshold definitions. However, increasing the threshold number for lipid lowering drugs attenuated the respective OR considerably (**Appendix 2**).

Adherence to prescription algorithms

The proportions of patients who should have received medication according to national guidelines and the ADDITION trial protocol and the proportions of patients who actually received a prescription within 3 months following the assessment of bio-medical data are presented in *column 1 and column 2* of **Figure 4**: The black part in *column 2* represents the proportion of patients who received a prescription and whose clinical values exceeded the thresholds for medication prescription and the framed white part represents the proportion of patients who received medication although clinical values did not exceed the thresholds. Adherence to the prescription algorithms, i.e. the proportion of patients who received at least one prescription out of those patients whose clinical values exceeded the thresholds (P [# of prescriptions ≥ 1] | [clinical value ≥ threshold]) is shown numerically in the lower part of **Figure 4**.

Due to tighter algorithms in the trial protocol (IT-arm) than in the national guidelines (RC-arm) more patients in the IT-arm were eligible for glucose-lowering, BP lowering and aspirin therapy than in the RC-arm. However, despite lower cholesterol thresholds in the IT-arm compared to the RC-arm, treatment with lipid lowering medication was indicated in almost equal proportions of patients in the two treatment arms.

For peer review only - http://bmjopen.bmj.com/site/about/guidelines.xhtml

Glucose lowering drugs: In the first year, the adherence to the treatment algorithm was generally low, but considerably higher in the IT-arm than in the RC-arm. At year 5, 73% of patients in both treatment arms with an HbA_{1c} ≥ threshold-level received a prescription.

BP- lowering/ACE inhibiting drugs: In the IT arm, adherence to the guideline for prescription of ACE inhibiting medication increased from 41% at baseline to 77% at year 5. In the RC arm, guideline adherence for prescription of any BP lowering medication increased from 55% at baseline to 94% at year 5 and 'prescription adherence' to ACE inhibiting medication (ACE inhibitors were not mentioned in the guidelines to be the first line treatment in RC) increased from 28% at baseline to 64% at year 5 (not shown). Of note, a large proportion of patients in the RC arm with BP levels below the threshold were prescribed BP lowering medication.

Lipid lowering drugs: Adherence to the treatment algorithms increased in both treatment arms and was consistently better in the IT-arm. At year 5, most patients with clinical values greater than threshold-levels were treated (IT-arm 93%, RC-arm 81%).

Aspirin: The adherence to the trial protocol/guidelines was low, less than 50% of eligible patients in both treatment arms received aspirin.

Discussion

Summary

ADDITION is a large pragmatic primary care based trial aiming to promote intensive multifactorial treatment of patients with screen detected diabetes by GPs. Utilizing electronic primary care records of patients, this study shows that GPs in the IT-arm did not see their patients more often, but were more likely to regularly prescribe metabolic and cardio-protective drugs. Generally, GPs' adherence to prescription algorithms increased substantially in both trial arms over the 5 year follow-up period. Large time-treatment interactions for prescription of glucose lowering medication indicates that background changes in routine care might have diluted the difference in treatment intensity over time.

For peer review only

Contextual frame

Pragmatic ("effectiveness") trials seek to produce externally valid results in order to inform the process of decision-making by policy makers [22-25]. However, unlike in explanatory ("efficacy") trials, adherence to protocol is rarely tightly monitored and the degree to which the intervention is implemented often remains uncertain. In the case of non-statistically significant results, this begs the question whether the intervention is *per se* not efficacious in the tested (heterogeneous) population, or whether the intended difference in treatment intensity was not big enough to detect any effects in the given sample size.

Lack of a difference in the intensity of treatment can be due to different reasons. Firstly, adherence of responsible health care professionals to the protocol might be low due to limited motivation, insufficient resources or lack of interest in the ongoing trial. To tackle this issue, in ADDITION-Cambridge, a detailed trial protocol was specified and the implementation of the protocol elements was incentivized by additional monetary resources and supported by an initial practice-based academic and two interactive feedback sessions[10].

Secondly, treatment delivered in everyday practice might differ from both guidelines and what happens in research-active practices. Not considering actual practice in routine care can result in intervention plans that fail to induce treatment differences between the trial arms. The choice of suitable interventions is therefore particularly challenging in multi-national trials like ADDITION, where guidelines or daily practice in countries might differ but a certain degree of intervention homogeneity is warranted[9].

Thirdly, policy changes, such as changes in the remuneration system and modifications in treatment guidelines, can intensify routine care, thus potentially diluting differences between the intervention and routine care arm. Long-term trials such as ADDITION are particularly susceptible to such influences. Between 2003 (~start of the study) and 2008/09 (~end of the 5 year analysis period) in the UK no new national diabetes treatment guidelines were released. However, in 2004 the Quality and Outcomes Framework (QOF) with its pay for performance system was launched [18] and extended in

For peer review only - http://bmjopen.bmj.com/site/about/guidelines.xhtml

the following years. The QOF incentivised fulfilment of basic quality of care indicators by monetary resources and may have improved the quality of care for patients with various conditions, including diabetes [20; 26].

Principal findings

Our study shows that although surgeries in the IT-arm received monetary resources for additional consultations, GPs and nurses did not see their patients more often, nor were they more likely to perform regular HbA_{1c}, lipid or UACR tests. This result might be explained by the fact that the patients in the RC-arm already saw their GP/nurse on average 5-6 times a year, which is more than the average ~4 GP and ~2.5 nurse contacts per year for the general UK population [27]. Therefore the GPs (and indeed the patients) may have felt that this was sufficient to adequately monitor the condition. It also shows that monetary incentives might help to convince a reasonable number of surgeries to participate in long-term extensive trials such as ADDITION (46% of contacted surgeries agreed to join the study), but that financial incentives might not be successful in motivating GPs to further increase treatment intensity if it is already at a high level [10]. In contrast, our results indicate that the education sessions and feedback audits had a positive impact on the protocol adherence of GPs, as in general adherence to the treatment algorithms in the IT-arm was higher than adherence to the national guidelines in the RC-arm. This finding supports previous research that feedback loops can help to maximize guideline adherence in primary care [28; 29].

According to the clinical thresholds outlined in the trial protocol and the national guidelines, more patients in the IT-arm than in the RC-arm were eligible to receive glucose-lowering, BP-lowering and platelet-inhibiting drugs (**Figure 4**). This suggests that the ADDITION intervention was designed at an appropriate level for the context, as even with a hypothetical prescription adherence of 100% patients in the IT-arm should have received more intensive treatment than patients in the RC-arm.

Notably, a very high proportion of patients in the RC-arm already received BP-lowering medication at baseline, although in many cases their BP levels did not exceed thresholds. The finding of high BP-lowering prescription prevalence probably results from the fact that treatment with BP lowering

For peer review only - http://bmjopen.bmj.com/site/about/guidelines.xhtml

medication was part of the risk-score used to identify high risk individuals eligible for diabetes screening in the first phase of the ADDITION trial [10]. There could be two reasons why many of the patients who received BP-lowering prescriptions had no apparent clinical indication for treatment. On the one hand, these patients might have previously had uncontrolled BP levels, but treatment with BP lowering medication brought their BP under control. On the other hand, it is possible that the daily practice for BP control at this time was already much stricter than recommended by the guidelines. Independently of its origin, the initially high prevalence of BP-lowering medication in both trial arms might be the reason why we did not observe a difference in the proportion of patients prescribed BP lowering drugs. Consequently, the observed difference in ACE inhibiting drugs may be due to GPs switching from diuretics or beta-blockers to ACE inhibiting drugs, as recommended by the trial protocol.

The low adherence to recommendations concerning aspirin therapy observed in both trial arms is interesting, as this prescription behaviour could be interpreted as a general scepticism among GPs (and perhaps patients) towards the weak evidence of benefits of aspirin therapy for primary prevention of cardiovascular disease [6]. The results of subsequent large trials justify such scepticism [30; 31]. Alternatively, some patients may have obtained aspirin from the pharmacy without a prescription without this being noted in the electronic medical record.

Except for aspirin, adherence to prescription algorithms increased substantially over the follow-up period. We assume that this finding is triggered by the progression and duration of the disease and by general improvements in the overall quality of care over time, independently of disease progression [32]. The significant interaction between 'treatment' and 'time since diagnosis' for glucose lowering medication indicates changing treatment patterns in the RC-arm which might be triggered by policy changes, like QOF. However, due to methodological limitations (covariate co-linearity, power problems in stratified models) this question could not be adequately addressed with the available data.

For peer review only - http://bmjopen.bmj.com/site/about/guidelines.xhtml

Implications for the planning of future pragmatic trials

This study shows that the successful implementation of a pragmatic trial in primary care is possible, but there are issues that need to be considered. Namely, (1) a high standard of care in control GP surgeries questions the need for further intensification, (2) treatment of patients in the RC-arm that did not reflect the national guidelines, and (3) background policy changes affecting quality of routine care. These issues need to be identified, considered and addressed when designing a pragmatic study or rolling out an intervention comprehensively [23; 24; 33]. The results further underline the potential importance of standard good practice in (pragmatic) trials. Methods such as initial academic detailing and repeated feedback sessions may be of great importance for the overall success of the study [24; 34]. In this context, more qualitative or quantitative implementation research may help to identify and test strategies that affect the adherence of health care professionals (and patients) [35].

Ideally, pragmatic trials of complex interventions should, if possible, be designed in a way that allows evaluation of the adherence of health care professionals to the trial protocol and of patients to the chosen treatment regimen. This study shows that the use of electronic primary care records is a promising approach for assessing the adherence of GPs. The obtained data are also useful for health economic research. In this particular example, the new primary care data can be used to update a previous analysis to reduce uncertainty in the cost-effectiveness of the intervention [36], a method consistent with an iterative approach to research and adoption decisions [37-39].

Implications for the interpretation of trial results

Intensified prescription algorithms were well implemented into practice. We found that prescription with glucose lowering, ACE inhibiting and lipid lowering drugs was higher in the IT-arm. The expected treatment effect resulting from this difference in medication could be interpreted as an area under the curve issue: The combination of the magnitude and the duration of the treatment difference can be expected to be the crucial driver of long-term effects. The extended follow-up of the UKPDS trial, which aimed to reduce diabetes related complications through tighter glucose and BP control, has shown that after the termination of the intervention, between-group differences in laboratory measurements disappeared [40-43]. However, the reductions in risk of micro- and macro-vascular

For peer review only - http://bmjopen.bmj.com/site/about/guidelines.xhtml

complications persisted (or increased) for patients who had received tight glucose control, but not for patients who had received tight BP control [40; 41]. In ADDITION we observed a small but significant improvement in HbA_{1c}, BP and cholesterol levels in the IT-arm and a non-significant reduction in risk of the composite CVD endpoint (RR=0.83, p=0.12) over a 5 year time period [14]. This study shows that the proportion of patients receiving glucose-lowering drugs in each arm had equalised at the end of the 5 year observation period, suggesting that the differences in glycaemic control might disappear in the subsequent years. However, as a substantially greater proportion of patients in the IT-arm received ACE inhibiting and lipid lowering drugs, it can be assumed that differences in BP and lipids might be sustained. If between-group differences in treatment for blood pressure and lipids diminish so will the levels of risk factors. However, the CVD risk may remain lower due to legacy effects of earlier reductions in glucose and cholesterol. Given that the number of events will also increase over time, it may be that the ADDITION intervention will show a statistically significant effect in the long-term; the ten year follow up of ADDITION will quantify the long term effect of relatively small differences in treatment and risk factors observed in the first 5 years after diagnosis of diabetes by screening [14].

Strengths and limitations

To our knowledge, this is one of the first studies to comprehensively analyse the adherence of GPs to a pragmatic trial protocol in primary care. In contrast to self-reported information from patients, electronically stored primary care records provide a high degree of detail about all GP-based primary care services delivered to patients and are less susceptible to recall bias [44]. Through the linkage of clinical information from the trial measurements with information on prescriptions from the electronic primary care records, it was further possible to comprehensively describe and analyse the prescription adherence of GPs to the trial protocol and national guidelines.

However, we only had data from a subsample of the ADDITION-Cambridge trial-cohort with an oversampling of patients with a primary event during the follow-up period. As our weighted sensitivity analyses showed that this issue did not affect the results, the findings of this study are likely to be generalizable to the sample of GP surgeries who participated in the ADDITION trial.

For peer review only - http://bmjopen.bmj.com/site/about/guidelines.xhtml

Nevertheless, the generalizability of results to average GP surgeries in the UK might be quite limited. In the experience of the authors, the practices that take part in research tend to be more organised and deliver better quality routine care than those declining to participate. This might lead to ceiling effects for interventions, i.e. it appears to be hard to induce a difference in treatment intensity between RC and a more intensive treatment regimen.

Another limitation is that in our assessment of prescription adherence, we did not take into account possible contra-indications for medications as well as patients' views, and analysed the data from a rather non-situational, disease-orientated perspective [45; 46]. Shared decision making between the GP and the patient might reasonably lead to decisions that deviate from those in the protocol (and national guidelines). We therefore do not know if patients or GPs were the main determinants of protocol non-adherence. It is possible that patients did not agree to start medication or to come to the surgery more often. To completely understand the adoption of the intervention the patient's role also needs to be taken into account, which was impossible with the chosen approach. Also, with the given data we could not evaluate the fidelity of GPs handing over the educational materials to study participants, which were also part of the intervention.

Finally, although the accuracy of primary care records for GP-based services is known to be quite high, particularly for prescribed medication and laboratory tests, the handling, merging and extraction of free text data from numerous observations (~80,000) originating from different IT format systems is challenging and validation was not undertaken [44]. Consequently, it is possible that a small proportion of services might be misclassified, resulting in non-differential bias.

For peer review only

Conclusion

This study demonstrates that the successful implementation of long-term pragmatic trials in primary care is possible, but there are many obstacles especially during periods of significant change in routine care. The retrospective analyses of the electronic primary care records of participants in the ADDITION-Cambridge trial shows that intensive treatment was fairly well implemented into practice, suggesting that positive effects on cardiovascular morbidity and mortality might be expected

For peer review only - http://bmjopen.bmj.com/site/about/guidelines.xhtml

in the long-term. Where possible, data needed to evaluate the fidelity of stakeholders to trial protocols should be collected routinely in future pragmatic trials as this information is invaluable for the interpretation of study results and for the planning of future studies.

Figure Legends

Figure 1:

Title: Study design

Figure 2:

Title: Adjusted mean number (and 95%-CI) of contacts with GPs and nurses according to Intensive Treatment (grey) and Routine Care (black); stratified from year 1 to year 5 after diagnosis

Legend:

Ɨ stratified linear regression models with a main effect for the intervention; adjusted for sex and age of diagnosis; accounted for patients being clustered within GP practices

ŀ overall linear regression models with a main effect for the intervention and for time since diagnosis and an interaction term between intervention and time; adjusted for sex and age of diagnosis; accounted for patients being clustered within GP practices and observations being clustered in patients

ł n=827 observations (n=169 in year 1, n=167 in year 2, n=168 in year 3, n=164 in year 4, and n=159 in year 5)

Figure 3:

Title: Proportion of patients receiving at least 4 prescriptions according to Intensive Treatment (grey) and Routine Care (black), stratified for year 1- 5 after diagnosis Ɨ

Legend:

Ɨ stratified logistic regression models with a main effect for the intervention; adjusted for sex and age of diagnosis; accounted for patients being clustered within GP practices

ŀ overall logistic regression models with a main effect for the intervention and for time since diagnosis and an interaction term between intervention and time; adjusted for sex and age of diagnosis; accounted for patients being clustered within GP practices and observations being clustered in patients

For peer review only - http://bmjopen.bmj.com/site/about/guidelines.xhtml

ł n=151 in year1, n=149 in year 2, n=150 in year 3, n=146 in year 4, and n=141 in year 5

n=737 observations (n=151 in year1, n=149 in year 2, n=150 in year 3, n=146 in year 4, and n=141 in year 5)

Figure 4

Title: Proportion of patients who should receive medication according to the ADDITION protocol and national guidelines and the proportion of patients who receive medication within a 3 month time period at baseline, year 1 and year 5 after diagnosis #

Legend:

# baseline, n=169; year 1, n=167; year 5, n=145

* i.e. medication indicated

Ɨ i.e. either well controlled patients or those receiving medication without indication

ꟷ i.e. poorly controlled patients or those receiving indicated medication

˧ Adherence with ADDITION protocol; ¥ Adherence with national guidelines

Conflict of interest statement

None of the authors has competing interests.

Data Sharing Statement

The access policy for sharing is based on the MRC Policy and Guidance on Sharing of Research Data from Population and Patient Studies. All data sharing must meet the terms of existing participants' consent and study ethical approvals.

Information on data and data requests can be found on http://epi-meta.medschl.cam.ac.uk/includes/addcam/addcam.html. In case of questions please contact datasharing@mrc-epid.cam.ac.uk.

For peer review only

Author contribution

ML, EW, CB and SG designed the concept for the paper. ML performed the statistical analysis, interpreted the data and drafted the manuscript. SG and CB were involved in collecting the data. EW provided statistical support. All authors critically revised the intellectual content of the manuscript and approved its final version.

Acknowledgements

We gratefully acknowledge the contribution of all participants, practice nurses and general practitioners in the ADDITION-Cambridge study (a full list of participating practices is given below) and Kit Coutts and Dr Rebecca Simmons for their contribution to data collection. We also acknowledge the contribution of the trial steering committee (Professors Nigel Stott (Chair), John Weinman, Richard Himsworth, and Paul Little) and Prof Jane Armitage and Dr Louise Bowman who adjudicated the endpoints.

Funding

ADDITION-Cambridge was supported by the Wellcome Trust (grant reference No G061895) the Medical Research Council (grant reference no: G0001164), National Health Service R&D support funding (including the Primary Care Research and Diabetes Research Networks), and the National Institute for Health Research. We received an unrestricted grant from University of Aarhus, Denmark, to support the ADDITION-Cambridge trial. Bio-Rad provided equipment to undertake capillary glucose screening by HbA1c in general practice. SG is a National Institute for Health Research (NIHR) Senior Investigator. The Primary Care Unit is supported by NIHR Research funds. SJG received support from the Department of Health NIHR Programme Grant funding scheme (RP-PG-0606-1259). EW is funded by the NIHR Cambridge Biomedical Research Centre. The views expressed in this publication are those of the authors and not necessarily those of the NHS, the NIHR, or the Department of Health.The Primary Care Unit and the Medical Research Council Epidemiology Unit at the University of Cambridge jointly coordinated the study. Aside from the authors, the ADDITION-Cambridge study team has included Rebecca Abbott, Amanda Adler, Judith Argles, Gisela Baker, Rebecca Bale, Ros Barling, Daniel Barnes, Mark Betts, Sue Boase, Sandra Bovan, Gwen Brierley, Ryan Butler, James Brimbicombe, Parinya Chamnan, Sean Dinneen, Pesheya

For peer review only - http://bmjopen.bmj.com/site/about/guidelines.xhtml

Doubleday, Justin Basile Echouffo-Tcheugui, Sue Emms, Mark Evans, Tom Fanshawe, Francis Finucane, Philippa Gash, Julie Grant, Wendy Hardeman, Robert Henderson, Susie Hennings, Muriel Hood, Garry King, Ann-Louise Kinmonth, Georgina Lewis, Christine May Hall, Joanna Mitchell, Richard Parker, Nicola Popplewell, A Toby Prevost, Emanuella De Lucia Rolfe, Richard Salisbury, Lincoln Sargeant, Rebecca Simmons, Stephen Sharp, Megan Smith, Stephen Sutton, Nicholas Wareham, Liz White, Fiona Whittle and Kate Williams. We also wish to thank the Cambridge University Hospitals NHS Foundation Trust Department of Clinical Biochemistry and the NIHR Cambridge Biomedical Research Centre Core Biochemistry Assay Laboratory for carrying out the biochemical assays and the following groups within the MRC Epidemiology Unit: data management (Adam Dickinson), information technology (Iain Morrison, Rich Hutchinson), technical (Matt Sims), and field epidemiology (Paul Roberts, Kim Mwanza, James Sylvester, Gwen Brierley, Jaimie Taylor). ADDITION-Cambridge practices: Acorn Community Health Centre, Arbury Road Surgery, Ashwell Surgery, Birchwood Surgery, Bridge Street Medical Centre, Brookfields & Cherry Hinton, Broomfields, Buckden Surgery, Burwell Surgery, Cambridge Surgery, Cedar House Surgery, Charles Hicks Centre, Chequers Lane Surgery, Clarkson Surgery, Cornerstone Practice, Cornford House Surgery, Cottenham Surgery, Cromwell Place Surgery, Dr Smith and Partner (Cambridge), East Field Surgery, Ely Surgery, Freshwell Health Centre, George Clare Surgery, Great Staughton Surgery, Harston Surgery, Health Centre (Eaton Socon), Hilton House, John Tasker House, Lensfield Medical Practice, Manea Surgery, Mercheford House, Milton Surgery, Nene Valley Medical Practice, Nevells Road Surgery, New Roysia Surgery, Northcote House Surgery, Nuffield Road Medical Centre, Orchard Surgery, Orchard House Surgery, Orton Medical Practice, Park Medical Centre, Paston Health Centre, Peterborough Surgery, Petersfield Medical Practice, Prior's Field Surgery, Queen Edith's Medical Practice, Queen Street Surgery, Rainbow Surgery, Ramsey Health Centre, Riverside Practice, Roman Gate Surgery, Rosalind Franklin House, South Street Surgery, Thaxted Surgery, The Health Centre (Bury St Edmunds), The Old Exchange, The Surgery Stanground, Townley Close Health Centre, Trumpington Street Medical Practice, Werrington Health Centre, York Street Medical Practice.

For peer review only - http://bmjopen.bmj.com/site/about/guidelines.xhtml

References

1. Morgan CL, Currie CJ, Peters JR: Relationship between diabetes and mortality: a population study using record linkage. Diabetes Care 2000;23:1103-1107
2. Narayan KM, Gregg EW, Fagot-Campagna A, Engelgau MM, Vinicor F: Diabetes--a common, growing, serious, costly, and potentially preventable public health problem. Diabetes Res Clin Pract 2000;50:S77-84
3. Fowler MJ: Microvascular and Macrovascular Complications of Diabetes. Clinical Diabetes 2008;26:77-82
4. Schunk M, Reitmeir P, Schipf S, Volzke H, Meisinger C, Thorand B, Kluttig A, Greiser KH, Berger K, Muller G, Ellert U, Neuhauser H, Tamayo T, Rathmann W, Holle R: Health-related quality of life in subjects with and without Type 2 diabetes: pooled analysis of five population-based surveys in Germany. Diabet Med 2012;29:646-653
5. Gaede P, Lund-Andersen H, Parving HH, Pedersen O: Effect of a multifactorial intervention on mortality in type 2 diabetes. N Engl J Med 2008;358:580-591
6. Gaede P, Vedel P, Larsen N, Jensen GV, Parving HH, Pedersen O: Multifactorial intervention and cardiovascular disease in patients with type 2 diabetes. N Engl J Med 2003;348:383-393
7. Clarke PM, Gray AM, Briggs A, Stevens RJ, Matthews DR, Holman RR: Cost-utility analyses of intensive blood glucose and tight blood pressure control in type 2 diabetes (UKPDS 72). Diabetologia 2005;48:868-877
8. Gray A, Raikou M, McGuire A, Fenn P, Stevens R, Cull C, Stratton I, Adler A, Holman R, Turner R: Cost effectiveness of an intensive blood glucose control policy in patients with type 2 diabetes: economic analysis alongside randomised controlled trial (UKPDS 41). BMJ 2000;320:1373-1378
9. Lauritzen T, Griffin S, Borch-Johnsen K, Wareham NJ, Wolffenbuttel BH, Rutten G: The ADDITION study: proposed trial of the cost-effectiveness of an intensive multifactorial intervention on morbidity and mortality among people with Type 2 diabetes detected by screening. Int J Obes Relat Metab Disord 2000;24:S6-11
10. Echouffo-Tcheugui JB, Simmons RK, Williams KM, Barling RS, Prevost AT, Kinmonth AL, Wareham NJ, Griffin SJ: The ADDITION-Cambridge trial protocol: a cluster -- randomised controlled trial of screening for type 2 diabetes and intensive treatment for screen-detected patients. BMC Public Health 2009;9:1471-2458
11. Li R, Zhang P, Barker LE, Chowdhury FM, Zhang X: Cost-Effectiveness of Interventions to Prevent and Control Diabetes Mellitus: A Systematic Review. Diabetes Care 2010;33:1872-1894
12. Sandbaek A, Griffin SJ, Sharp SJ, Simmons RK, Borch-Johnsen K, Rutten GE, van den Donk M, Wareham NJ, Lauritzen T, Davies MJ, Khunti K: Effect of early multifactorial therapy compared with routine care on microvascular outcomes at 5 years in people with screen-detected diabetes: a randomized controlled trial: the ADDITION-Europe Study. Diabetes Care 2014;37:2015-2023
13. Simmons RK, Sharp SJ, Sandbaek A, Borch-Johnsen K, Davies MJ, Khunti K, Lauritzen T, Rutten GE, van den Donk M, Wareham NJ, Griffin SJ: Does early intensive multifactorial treatment reduce total cardiovascular burden in individuals with screen-detected diabetes? Findings from the ADDITION-Europe cluster-randomized trial. Diabet Med 2012;29:1464-5491
14. Griffin SJ, Borch-Johnsen K, Davies MJ, Khunti K, Rutten GE, Sandbaek A, Sharp SJ, Simmons RK, van den Donk M, Wareham NJ, Lauritzen T: Effect of early intensive multifactorial therapy on 5-year cardiovascular outcomes in individuals with type 2 diabetes detected by screening (ADDITION-Europe): a cluster-randomised trial. Lancet 2011;378:156-167

For peer review only - http://bmjopen.bmj.com/site/about/guidelines.xhtml

15. McIntosh AHA, Home PD, Brown F, Bruce A, Damerell A, Davis R, et al. Clinical guidelines and evidence review for Type 2 diabetes: management of blood glucose. University of Sheffield, 2001.
16. McIntosh AHA, Home PD, Brown F, Bruce A, Damerell A, Davis R, et al. Clinical guidelines and evidence review for Type 2 diabetes: management of blood pressure. University of Sheffield, 2002.
17. McIntosh AHA, Home PD, Brown F, Bruce A, Damerell A, Davis R, et al. Clinical guidelines and evidence review for Type 2 diabetes: Lipids management. University of Sheffield, 2002.
18. Health and Social Care Information Centre. National Quality and Outcomes Framework Statistics for England - 2004-05. retrieved from http://www.hscic.gov.uk/catalogue/PUB01946. last accessed: 05.02.2015.
19. Health and Social Care Information Centre. Quality and Outcomes Framework - 2006-07. retrieved from http://www.hscic.gov.uk/catalogue/PUB05997. last accessed: 05.02.2015.
20. Campbell S, Reeves D, Kontopantelis E, Middleton E, Sibbald B, Roland M: Quality of Primary Care in England with the Introduction of Pay for Performance. New England Journal of Medicine 2007;357:181-190
21. Clarke PM, Gray AM, Briggs A, Farmer AJ, Fenn P, Stevens RJ, Matthews DR, Stratton IM, Holman RR: A model to estimate the lifetime health outcomes of patients with type 2 diabetes: the United Kingdom Prospective Diabetes Study (UKPDS) Outcomes Model (UKPDS no. 68). Diabetologia 2004;47:1747-1759
22. Singal AG, Higgins PDR, Waljee AK: A Primer on Effectiveness and Efficacy Trials. Clin Trans Gastroenterol 2014;5:e45
23. Patsopoulos NA: A pragmatic view on pragmatic trials. Dialogues in Clinical Neuroscience 2011;13:217-224
24. Eldridge S: Pragmatic trials in primary health care: what, when and how? Family Practice 2010;27:591-592
25. Roland M, Torgerson DJ: What are pragmatic trials? BMJ 1998;316:285
26. Khunti K, Gadsby R, Millett C, Majeed A, Davies M: Quality of diabetes care in the UK: comparison of published quality-of-care reports with results of the Quality and Outcomes Framework for Diabetes. Diabetic Medicine 2007;24:1436-1441
27. Redekop WK, Koopmanschap MA, Stolk RP, Rutten GE, Wolffenbuttel BH, Niessen LW: Health-related quality of life and treatment satisfaction in Dutch patients with type 2 diabetes. Diabetes Care 2002;25:458-463
28. Ivers N, Jamtvedt G, Flottorp S, Young JM, Odgaard-Jensen J, French SD, O'Brien MA, Johansen M, Grimshaw J, Oxman AD: Audit and feedback: effects on professional practice and healthcare outcomes. Cochrane Database Syst Rev 2012;13
29. Goff DC, Jr., Massing MW, Bertoni AG, Davis J, Ambrosius WT, McArdle J, Duren-Winfield V, Sueta CA, Croft JB: Enhancing quality of heart failure care in managed Medicare and Medicaid in North Carolina: results of the North Carolina Achieving Cardiac Excellence (NC ACE) Project. Am Heart J 2005;150:717-724
30. Belch J, MacCuish A, Campbell I, Cobbe S, Taylor R, Prescott R, Lee R, Bancroft J, MacEwan S, Shepherd J, Macfarlane P, Morris A, Jung R, Kelly C, Connacher A, Peden N, Jamieson A, Matthews D, Leese G, McKnight J, O'Brien I, Semple C, Petrie J, Gordon D, Pringle S, MacWalter R, Prevention of Progression of Arterial D, Diabetes Study Group DRG, Royal College of Physicians E: The prevention of progression of arterial disease and diabetes (POPADAD) trial: factorial randomised placebo controlled trial of aspirin and antioxidants in patients with diabetes and asymptomatic peripheral arterial disease. BMJ : British Medical Journal 2008;337:a1840
31. Ogawa H, Nakayama M, Morimoto T, et al.: Low-dose aspirin for primary prevention of atherosclerotic events in patients with type 2 diabetes: A randomized controlled trial. JAMA 2008;300:2134-2141

For peer review only - http://bmjopen.bmj.com/site/about/guidelines.xhtml

32. Filion KB, Joseph L, Boivin JF, Suissa S, Brophy JM: Trends in the prescription of anti-diabetic medications in the United Kingdom: a population-based analysis. Pharmacoepidemiol Drug Saf 2009;18:973-976
33. Glasgow RE: What Does It Mean to Be Pragmatic? Pragmatic Methods, Measures, and Models to Facilitate Research Translation. Health Education & Behavior 2013;40:257-265
34. Godwin M, Ruhland L, Casson I, MacDonald S, Delva D, Birtwhistle R, Lam M, Seguin R: Pragmatic controlled clinical trials in primary care: the struggle between external and internal validity. BMC Med Res Methodol 2003;3:28
35. Peters DH, Adam T, Alonge O, Agyepong IA, Tran N: Implementation research: what it is and how to do it. BMJ 2013;347:f6753
36. Tao L, Wilson EC, Wareham NJ, Sandbaek A, Rutten GE, Lauritzen T, Khunti K, Davies MJ, Borch-Johnsen K, Griffin SJ, Simmons RK: Cost-effectiveness of intensive multifactorial treatment compared with routine care for individuals with screen-detected Type 2 diabetes: analysis of the ADDITION-UK cluster-randomized controlled trial. Diabet Med 2015;6:12711
37. Wilson E, Abrams K: From Evidence Based Economics to Economics Based Evidence: Using Systematic Review to inform the design of future research. In *Evidence Based Economics* Shemilt I, Mugford M, Vale L, Marsh K, Donaldson C, Eds. London, Blackwell Publishing, 2010
38. Claxton K, Cohen JT, Neumann PJ: When is evidence sufficient? Health Aff (Millwood) 2005;24:93-101
39. Sculpher M, Drummond M, Buxton M: The iterative use of economic evaluation as part of the process of health technology assessment. J Health Serv Res Policy 1997;2:26-30
40. Holman RR, Paul SK, Bethel MA, Matthews DR, Neil HAW: 10-Year Follow-up of Intensive Glucose Control in Type 2 Diabetes. New England Journal of Medicine 2008;359:1577-1589
41. Holman RR, Paul SK, Bethel MA, Neil HAW, Matthews DR: Long-Term Follow-up after Tight Control of Blood Pressure in Type 2 Diabetes. New England Journal of Medicine 2008;359:1565-1576
42. Tight blood pressure control and risk of macrovascular and microvascular complications in type 2 diabetes: UKPDS 38. UK Prospective Diabetes Study Group. Bmj 1998;317:703-713
43. Intensive blood-glucose control with sulphonylureas or insulin compared with conventional treatment and risk of complications in patients with type 2 diabetes (UKPDS 33). UK Prospective Diabetes Study (UKPDS) Group. Lancet 1998;352:837-853
44. Byford S, Leese M, Knapp M, Seivewright H, Cameron S, Jones V, Davidson K, Tyrer P: Comparison of alternative methods of collection of service use data for the economic evaluation of health care interventions. Health Econ 2007;16:531-536
45. Juul L, Sandbaek A, Foldspang A, Frydenberg M, Borch-Johnsen K, Lauritzen T: Adherence to guidelines in people with screen-detected type 2 diabetes, ADDITION, Denmark. Scand J Prim Health Care 2009;27:223-231
46. Zoffmann V, Harder I, Kirkevold M: A person-centered communication and reflection model: sharing decision-making in chronic care. Qual Health Res 2008;18:670-685

For peer review only

For peer review only - http://bmjopen.bmj.com/site/about/guidelines.xhtml

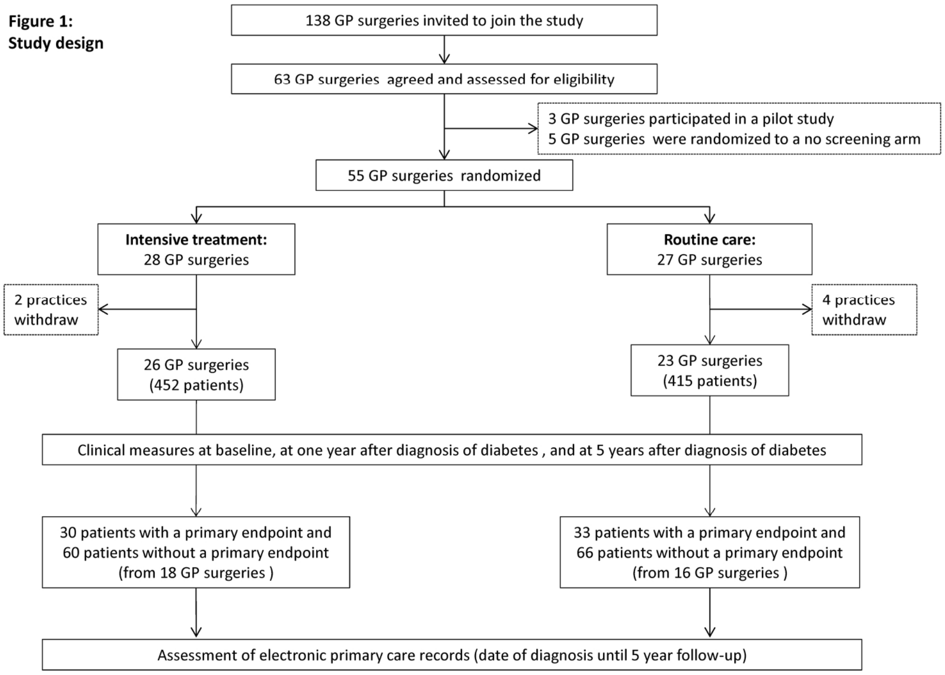

Figure 1

190x142mm (300 x 300 DPI)

Figure 2:

Adjusted mean number(and 95%-CI) of contacts with GPs and nurses according to Intensive Treatment (grey) and Routine Care (black); stratified from year 1 to year 5 after diagnosis ǂ

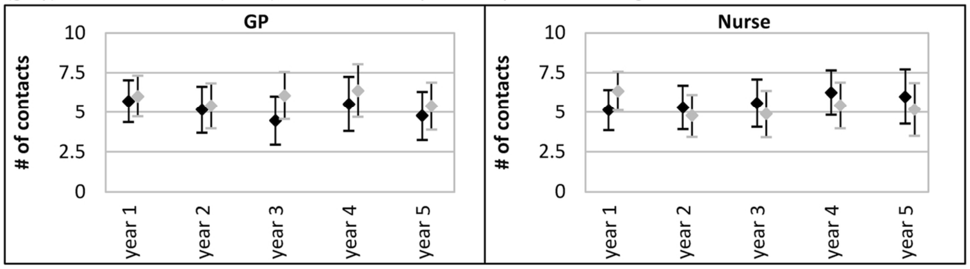

Overall adjusted mean number of contacts with GPs and nurses per year according to Routine Care and Intensive Treatment ˫

	adj. mean (95%CI) ǂ		adj. mean (95%CI) ǂ
Intensive Treatment	5.80 (4.68, 6.93)	Intensive Treatment	5.34 (4.22, 6.47)
Routine Care	5.15 (4.01, 6.29)	Routine Care	5.49 (4.33, 6.65)
Difference (IT vs. RC)	0.65 (-0.95, 2.26)	Difference (IT vs. RC)	-0.15 (-1.77, 1.48)
time since diagnosis (years)	-0.05 (-0.24, 0.13)	time since diagnosis (years)	0.02 (-0.17, 0.21)
p-value (time x treatment)	0.513	p-value (time x treatment)	0.093

ǂ stratified linear regression models with a main effect for the intervention; adjusted for sex and age of diagnosis; accounted for patients being clustered within GP practices

˫ overall linear regression models with a main effect for the intervention and for time since diagnosis and an interaction term between intervention and time; adjusted for sex and age of diagnosis; accounted for patients being clustered within GP practices and observations being clustered in patients

ǂ n=827 observations (n=169 in year 1, n=167 in year 2, n=168 in year 3, n=164 in year 4, and n=159 in year 5)

Figure 2

127x99mm (300 x 300 DPI)

Figure 3:
Proportion of patients receiving at least 4 prescriptions according to Intensive Treatment (grey) and Routine Care (black), stratified for year 1- 5 after diagnosis

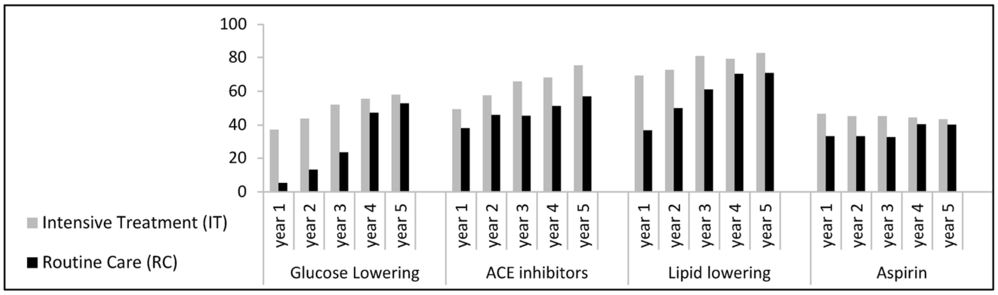

Odds Ratio of having received at least 4 prescriptions per year IT vs. RC (reference)

**Stratified by year ǂ**	**OR (95%-CI) ǂ**	**OR (95%-CI) ǂ**	**OR (95%-CI) ǂ**	**OR (95%-CI) ǂ**
Year 1 (IC vs. RC)	10.89 (3.53, 33.56)	1.57 (0.73, 3.37)	4.00 (1.95, 8.20)	1.67 (0.72, 3.85)
Year 2 (IC vs. RC)	5.88 (2.51, 13.80)	1.60 (0.82, 3.09)	2.63 (1.31, 5.26)	1.66 (0.72, 3.86)
Year 3 (IC vs. RC)	3.78 (1.76, 8.10)	2.34 (1.18, 4.64)	2.63 (1.15, 6.01)	1.60 (0.62, 4.09)
Year 4 (IC vs. RC)	1.42 (0.73, 2.76)	2.06 (1.02, 4.14)	1.57 (0.68, 3.63)	1.16 (0.37, 3.61)
Year 5 (IC vs. RC)	1.23 (0.62, 2.42)	2.66 (1.14, 6.21)	1.99 (0.88, 4.53)	1.22 (0.43, 3.50)
**Year 1-5 Ͱ**	**OR (95%-CI) #**	**OR (95%-CI) #**	**OR (95%-CI) #**	**OR (95%-CI) #**
Overall (IC vs. RC)	3.27 (1.81, 5.93)	2.03 (1.13, 3.65)	2.42 (1.3, 4.51)	1.41 (0.61, 3.24)
Time since diagnosis (per year)	1.61 (1.42, 1.83)	1.25 (1.12, 1.39)	1.33 (1.18, 1.5)	1.04 (0.93, 1.15)
p-value (time x treatment)	<.0001	0.331	0.131	0.220

ǂ stratified logistic regression models with a main effect for the intervention; adjusted for sex and age of diagnosis; accounted for patients being clustered within GP practices

Ͱ overall logistic regression models with a main effect for the intervention and for time since diagnosis and an interaction term between intervention and time; adjusted for sex and age of diagnosis; accounted for patients being clustered within GP practices and observations being clustered in patients

ǂ n=151 in year1, n=149 in year 2, n=150 in year 3, n=146 in year 4, and n=141 in year 5

n=737 observations (n=151 in year1, n=149 in year 2, n=150 in year 3, n=146 in year 4, and n=141 in year 5)

Figure 3

159x130mm (300 x 300 DPI)

Figure 4:
Proportion of patients who should receive medication according to the ADDITION protocol and national guidelines and the proportion of patients who receive medication within a 3 month time period at baseline, year 1 and year 5 after diagnosis [#]

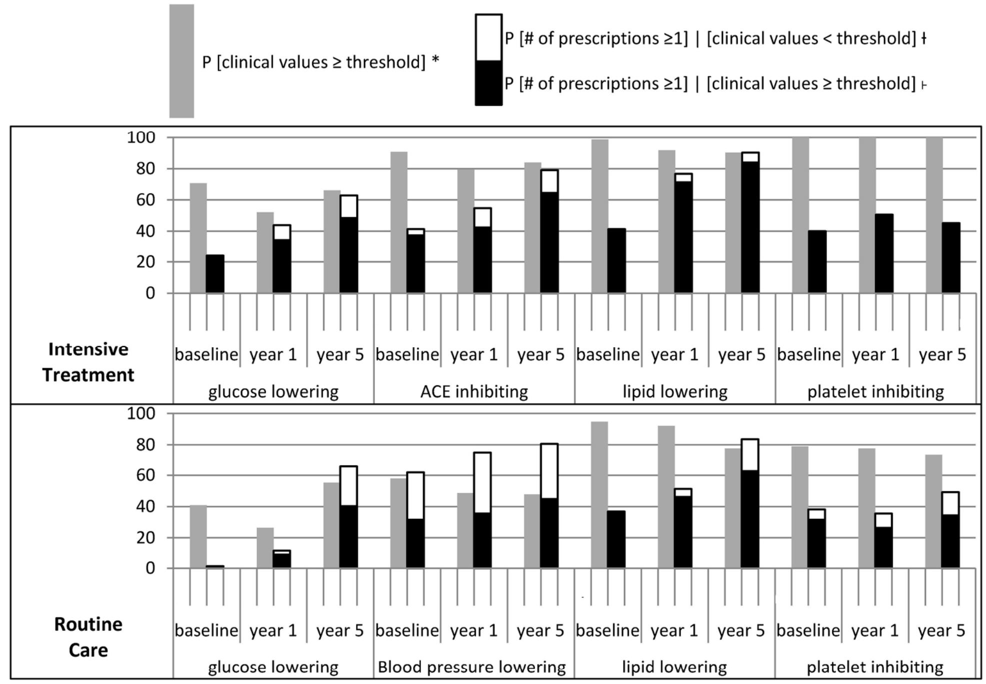

Prescription adherence (P [# of prescriptions ≥1] | [clinical value ≥ threshold])

IT-arm ɫ	0.32	0.66	0.73	0.41	0.53	0.77	0.42	0.78	0.93	0.40	0.51	0.45
RC-arm ¥	0.03	0.35	0.73	0.55	0.73	0.94	0.39	0.50	0.81	0.40	0.34	0.47

[#] baseline, n=169; year 1, n=167; year 5, n=145
* i.e. medication indicated
ɫ i.e. either well controlled patients or those receiving medication without indication
⊦ i.e. poorly controlled patients or those receiving indicated medication
ɫ Adherence with ADDITION protocol; ¥ Adherence with national guidelines

Figure 4

178x171mm (300 x 300 DPI)

Supplementary Material

Appendix 1: Proportion of patients receiving regular monitoring for HbA_{1c}, cholesterol and albuminuria and proportion of patients receiving blood pressure lowering medication

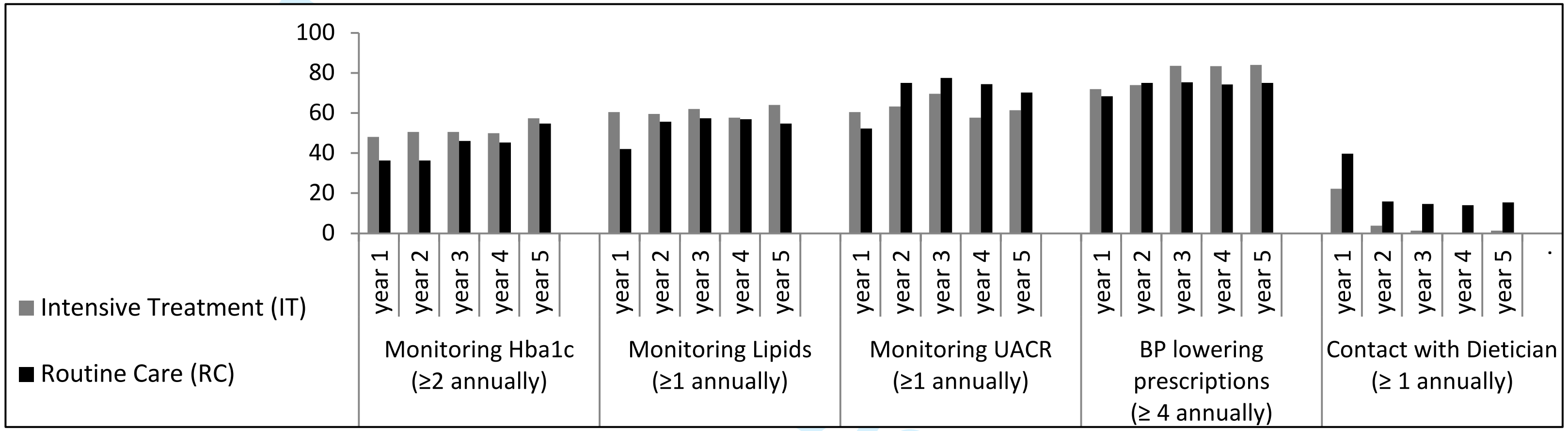

**Stratified by year ƚ**	**OR (95%-CI) ǂ**	**OR (95%-CI) ǂ**	**OR (95%-CI) ǂ**	**OR (95%-CI) #**	**OR (95%-CI) ǂ**
Year 1 (IT vs. RC)	2.00 (0.44, 9.02)	2.66 (0.53, 13.22)	1.41 (0.46, 4.32)	1.15 (0.55, 2.4)	0.88 (0.24, 3.26)
Year 2 (IT vs. RC)	2.29 (0.41, 12.63)	1.30 (0.23, 7.20)	0.62 (0.15, 2.60)	0.95 (0.45, 2.01)	0.38 (0.07, 2.18)
Year 3 (IT vs. RC)	1.28 (0.36, 4.52)	1.96 (0.36, 10.68)	0.93 (0.24, 3.56)	1.69 (0.72, 3.95)	0.12 (0.01, 1.39)
Year 4 (IT vs. RC)	1.52 (0.46, 5.03)	1.28 (0.29, 5.73)	0.49 (0.17, 1.45)	1.76 (0.72, 4.3)	-
Year 5 (IT vs. RC)	1.15 (0.44, 3.03)	2.3 (0.47, 11.32)	0.72 (0.30, 1.77)	1.89 (0.70, 5.15)	0.08 (0.01, 0.67)
**Year 1-5 ⊦**	**OR (95%-CI) ¬**	**OR (95%-CI) ¬**	**OR (95%-CI) ¬**	**OR (95%-CI) ˧**	**OR (95%-CI) ¬**
Overall (IC vs. SC)	1.56 (0.63, 3.83)	1.53 (0.51, 4.6)	0.82 (0.34, 1.98)	1.41 (0.71, 2.8)	0.43 (0.32, 0.58)
Time since diagnosis (years)	1.12 (1.01, 1.23)	1.05 (0.95, 1.16)	1.08 (0.97, 1.2)	1.15 (1.02, 1.31)	0.13 (0.04, 0.45)
p-value (time x treatment)	0.294	0.303	0.075	0.223	0.001

ƚ stratified logistic regression models with a main effect for the intervention; adjusted for sex and age of diagnosis; accounted for patients being clustered within GP practices

⊦ overall logistic regression models with a main effect for the intervention and for time since diagnosis and an interaction term between intervention and time; adjusted for sex and age of diagnosis; accounted for patients being clustered within GP practices and observations being clustered in patients

ǂ n=169 in year 1, n=167 in year 2, n=168 in year 3, n=164 in year 4, and n=159 in year 5

n=151 in year1, n=149 in year 2, n=150 in year 3, n=146 in year 4 and n=141 in year 5

¬ n=827 observations (n=169 in year 1, n=167 in year 2, n=168 in year 3, n=164 in year 4, and n=159 in year 5)

˧ n=737 observations (n=151 in year1, n=149 in year 2, n=150 in year 3, n=146 in year 4, and n=141 in year 5)

HbA_{1c} hemoglobin A1c; UACR urine-albumin-creatinine-ratio; BP blood pressure

For peer review only - http://bmjopen.bmj.com/site/about/guidelines.xhtml

Appendix 2: Results of various sensitivity analyses

	Adjusted odds ratio of having received 'continuous medication', IT vs. RC (reference)ǂ				Difference in adjusted mean number of contacts with GPs and nurses, IT vs. RC (reference) ⊦	
	**OR (95-% CI)**				**adjusted mean difference (95%-CI)**	
	Glucose-lowering	ACE-inhibiting	lipid-lowering	aspirin	# of GP contacts	# of nurse contacts
main model (from Figure 2 & 3)	*3.27 (1.81, 5.93) ¬*	*2.03 (1.13, 3.65) ¬*	*2.42 (1.30, 4.51) ¬*	*1.41 (0.61, 3.24) ¬*	*0.65 (-0.95, 2.26)˧*	*-0.15 (-1.77, 1.48) ˧*
a) weighted model	2.89 (1.51, 5.53) ¬	2.13 (1.15, 3.93) ¬	2.54 (1.32, 4.92) ¬	1.47 (0.59, 3.69) ¬	0.81 (-0.79, 2.42) ˧	0.21 (-1.40, 1.81) ˧
b) multiple imputed model	3.06 (1.78, 5.28) ǂ	2.05 (1.20, 3.50) ǂ	2.37 (1.32, 4.25) ǂ	1.32 (0.62, 2.80) ǂ	0.68 (-0.9, 2.26) ǂ	-0.10 (-1.70, 1.50) ǂ
c) threshold: ≥ 2 prescriptions annually	3.07 (1.68, 5.61) ¬	2.10 (1.12, 3.94) ¬	2.16 (1.13, 4.14) ¬	1.45 (0.70, 3.02) ¬	-	-
d) threshold: ≥ 6 prescriptions annually	3.97 (2.17, 7.26) ¬	2.24 (1.25, 4.03) ¬	2.35 (1.24, 4.45) ¬	1.40 (0.57, 3.46) ¬	-	-
e) threshold: ≥ 12 prescriptions annually	4.86 (2.34, 10.1) ¬	1.79 (0.79, 4.06) ¬	1.35 (0.58, 3.12) ¬	1.04 (0.37, 2.97) ¬	-	-

ǂ overall logistic regression models with a main effect for the intervention and for time since diagnosis and an interaction term between intervention and time; adjusted for sex and age of diagnosis; accounted for patients being clustered within GP practices and observations being clustered in patients

⊦ overall linear regression models with a main effect for the intervention and for time since diagnosis and an interaction term between intervention and time; adjusted for sex and age of diagnosis; accounted for patients being clustered within GP practices and observations being clustered in patients

¬ n=737 observations (n=151 in year1, n=149 in year 2, n=150 in year 3, n=146 in year 4, and n=141 in year 5)

ǂ n=885 observations (n=173 from year1 to year 5)

a) individuals weighted by the inverse probability of being in the sample given the status on the primary endpoint

b) multiple imputed dataset of participants with at least partially missing information on electronic primary care records in year 1 to 5 (PROC MI/PROC MIANALYZE)

c) threshold for 'continuous medication' changed to '≥ 2 prescriptions annually'

d) threshold for 'continuous medication' changed to '≥ 6 prescriptions annually'

e) threshold for 'continuous medication' changed to '≥ 12 prescriptions annually'

For peer review only

For peer review only - http://bmjopen.bmj.com/site/about/guidelines.xhtml

It needs to be acknowledged that the study does not report the primary or secondary outcomes of the trial (they have been reported elsewhere), but it reports the adherence of GPs to the trial protocol. Therefore, several points that are highly important in reporting the results of a trial are of inferior importance in reporting the adherence of GPs to the protocol.

CONSORT 2010 checklist of information to include when reporting a randomised trial*

Section/Topic	Item No	Checklist item	Reported on page No
**Title and abstract**			
	1a	Identification as a randomised trial in the title	1
	1b	Structured summary of trial design, methods, results, and conclusions (for specific guidance see CONSORT for abstracts)	3-4
**Introduction**			
Background and objectives	2a	Scientific background and explanation of rationale	5-6
	2b	Specific objectives or hypotheses	6
**Methods**			
Trial design	3a	Description of trial design (such as parallel, factorial) including allocation ratio	6-7
	3b	Important changes to methods after trial commencement (such as eligibility criteria), with reasons	Na
Participants	4a	Eligibility criteria for participants	6-7
	4b	Settings and locations where the data were collected	6-7
Interventions	5	The interventions for each group with sufficient details to allow replication, including how and when they were actually administered	7-8
Outcomes	6a	Completely defined pre-specified primary and secondary outcome measures, including how and when they were assessed	8-9
	6b	Any changes to trial outcomes after the trial commenced, with reasons	Na
Sample size	7a	How sample size was determined	6-7
	7b	When applicable, explanation of any interim analyses and stopping guidelines	Na
Randomisation:			
Sequence generation	8a	Method used to generate the random allocation sequence	Na
	8b	Type of randomisation; details of any restriction (such as blocking and block size)	Na
Allocation	9	Mechanism used to implement the random allocation sequence (such as sequentially numbered containers),	Na

For peer review only - http://bmjopen.bmj.com/site/about/guidelines.xhtml

concealment mechanism		describing any steps taken to conceal the sequence until interventions were assigned	
Implementation	10	Who generated the random allocation sequence, who enrolled participants, and who assigned participants to interventions	Na
Blinding	11a	If done, who was blinded after assignment to interventions (for example, participants, care providers, those assessing outcomes) and how	Na
	11b	If relevant, description of the similarity of interventions	na
Statistical methods	12a	Statistical methods used to compare groups for primary and secondary outcomes	9-10
	12b	Methods for additional analyses, such as subgroup analyses and adjusted analyses	9-10
**Results**			
Participant flow (a diagram is strongly recommended)	13a	For each group, the numbers of participants who were randomly assigned, received intended treatment, and were analysed for the primary outcome	Figure 1
	13b	For each group, losses and exclusions after randomisation, together with reasons	Figure 1
Recruitment	14a	Dates defining the periods of recruitment and follow-up	8
	14b	Why the trial ended or was stopped	Na
Baseline data	15	A table showing baseline demographic and clinical characteristics for each group	Table 1
Numbers analysed	16	For each group, number of participants (denominator) included in each analysis and whether the analysis was by original assigned groups	Figure 2-4
Outcomes and estimation	17a	For each primary and secondary outcome, results for each group, and the estimated effect size and its precision (such as 95% confidence interval)	Figure 2-4
	17b	For binary outcomes, presentation of both absolute and relative effect sizes is recommended	Na
Ancillary analyses	18	Results of any other analyses performed, including subgroup analyses and adjusted analyses, distinguishing pre-specified from exploratory	Na
Harms	19	All important harms or unintended effects in each group (for specific guidance see CONSORT for harms)	Na
**Discussion**			
Limitations	20	Trial limitations, addressing sources of potential bias, imprecision, and, if relevant, multiplicity of analyses	18-19
Generalisability	21	Generalisability (external validity, applicability) of the trial findings	18-19
Interpretation	22	Interpretation consistent with results, balancing benefits and harms, and considering other relevant evidence	Na
**Other information**			
Registration	23	Registration number and name of trial registry	4
Protocol	24	Where the full trial protocol can be accessed, if available	6 (ref 10)
Funding	25	Sources of funding and other support (such as supply of drugs), role of funders	20-21

For peer review only - http://bmjopen.bmj.com/site/about/guidelines.xhtml

*We strongly recommend reading this statement in conjunction with the CONSORT 2010 Explanation and Elaboration for important clarifications on all the items. If relevant, we also recommend reading CONSORT extensions for cluster randomised trials, non-inferiority and equivalence trials, non-pharmacological treatments, herbal interventions, and pragmatic trials. Additional extensions are forthcoming: for those and for up to date references relevant to this checklist, see www.consort-statement.org.

For peer review only

For peer review only - http://bmjopen.bmj.com/site/about/guidelines.xhtml

BMJ Open

How good are GPs at adhering to a pragmatic trial protocol in primary care? Results from the ADDITION-Cambridge cluster-randomized pragmatic trial

Journal:	*BMJ Open*
Manuscript ID	bmjopen-2016-015295.R2
Article Type:	Research
Date Submitted by the Author:	01-Jun-2017
Complete List of Authors:	Laxy, Michael; Helmholtz Zentrum München, Institute of Health Economics and Health Care Management<br>Wilson, Edward; University of Cambridge, Cambridge Centre for Health Services Research<br>Boothby, Clare; Institute of Metabolic Science, MRC Epidemiology Unit<br>Griffin, Simon; Institute of Metabolic Science, MRC Epidemiology Unit
**Primary Subject Heading**:	Health services research
Secondary Subject Heading:	Diabetes and endocrinology
Keywords:	diabetes mellitus, pragmatic trial, protocol adherence, PRIMARY CARE

SCHOLARONE™
Manuscripts

For peer review only

How good are GPs at adhering to a pragmatic trial protocol in primary care? Results from the ADDITION-Cambridge cluster-randomized pragmatic trial

Authors: Michael Laxy[1,2,3], Edward C.F. Wilson[4], Clare E. Boothby[3], Simon J. Griffin[3]

[1] Institute of Health Economics, Helmholtz Zentrum München, Neuherberg, Germany, Ingolstädter Landstraße 1, 85764 München

[2] German Center for Diabetes Research, Neuherberg, Germany, Ingolstädter Landstraße 1, 85764 München

[3] MRC Epidemiology Unit, University of Cambridge, Cambridge, UK, Cambridge Biomedical Campus, Cambridge CB2 0SL

[4] Cambridge Centre for Health Services Research, University of Cambridge, Cambridge, UK, Forvie Site Cambridge Biomedical Campus, Cambridge CB2 0SR

Email addresses:

Michael Laxy: michael.laxy@helmholtz-muenchen.de

Ed Wilson: ed.wilson@medschl.cam.ac.uk

Clare Boothby: Clare.Boothby@mrc-epid.cam.ac.uk

Simon Griffin: sjg49@medschl.cam.ac.uk

Corresponding author: Dr. Ed Wilson, Cambridge Centre for Health Services Research, University of Cambridge, Institute of Public Health, Forvie Site, Robinson Way, Cambridge CB2 0SR; Email: ed.wilson@medschl.cam.ac.uk

Manuscript word count: 5,070

Key words: diabetes mellitus, pragmatic trial, protocol adherence, primary care

For peer review only - http://bmjopen.bmj.com/site/about/guidelines.xhtml

For peer review only

Abstract

Objective: To assess the fidelity of general practitioners' (GP) adherence to a long term pragmatic trial protocol.

Design: Retrospective analyses of electronic primary care records of participants in the pragmatic cluster-randomised ADDITION (Anglo-Danish-Dutch Study of Intensive Treatment In People with Screen Detected Diabetes in Primary Care)-Cambridge trial, comparing intensive multi-factorial treatment (IT) vs. routine care (RC). Data were collected from the date of diagnosis until December 2010.

Setting: Primary care surgeries in the East of England

Study sample/participants: A subsample (n=189, RC-arm: n=99, IT-arm: n=90) of patients from the ADDITION-Cambridge cohort (867 patients), consisting of 40-69 year old patients with screen detected diabetes mellitus.

Interventions: In the RC-arm treatment was delivered according to concurrent treatment guidelines. Surgeries in the IT-arm received funding for additional contacts between GPs/nurses and patients, and GPs were advised to follow more intensive treatment algorithms for the management of glucose, lipids and blood pressure and aspirin therapy than in the RC-arm.

Outcome measures: The number of annual contacts between patients and GPs/nurses, the proportion of patients receiving prescriptions for cardio-metabolic medication in years 1 to 5 after diabetes diagnosis, and the adherence to prescription algorithms.

Results: The difference in the number of annual GP contacts (β=0.65) and nurse contacts (β=-0.15) between the study arms was small and insignificant. Patients in the IT-arm were more likely to receive glucose-lowering (OR=3.27), ACE-inhibiting (OR=2.03) and lipid-lowering drugs (OR=2.42, all p-values<0.01) than patients in the RC-arm. The prescription adherence varied between medication classes, but improved in both trial arms over the 5 year follow-up.

For peer review only - http://bmjopen.bmj.com/site/about/guidelines.xhtml

For peer review only

Conclusions: The adherence of GPs to different aspects of the trial protocol was mixed. Background changes in health care policy need to be considered as they have the potential to dilute differences in treatment intensity and hence incremental effect.

Clinical trial number: ISRCTN86769081

Article Summary: Strengths and Limitations of the Study

- Pragmatic trials aim to produce externally valid results for decision makers. If and to what extent pragmatic trial interventions are delivered to patients often remains unknown.
- This study describes the adherence of GPs to the ADDITION trial protocol and hence provides a unique insight about what we can expect in future long-term pragmatic studies in the primary care context, particularly in the context of policy and guideline changes.
- Analyses are based on a subsample of participants of the ADDITION-Cambridge trial conducted in the East of England. Therefore, the generalizability of results might be limited.

For peer review only

Background

Type 2 diabetes is an increasing public health problem associated with premature mortality and costly micro- and macro-vascular complications in terms of both reduced quality of life and financial burden, causing substantial economic pressure on healthcare systems and societies [1-4].

Previous research has shown that intensive treatment of cardiovascular risk factors is an effective and cost-effective intervention for patients with longstanding diabetes or routinely diagnosed diabetes [5-8]. In contrast, little was known about the cost-effectiveness of intensive primary care based treatment in patients in the early stages of the disease, such as screen detected populations. The pragmatic cluster-randomised ADDITION (**A**nglo-**D**anish-**D**utch Study of **I**ntensive **T**reatment **I**n People with Scree**n** Detected Diabetes in Primary Care) - trial (ISRCTN86769081) was one of the first studies addressing this important question [9-11]. Results showed that, compared to routine care, early intensive treatment modestly improved levels of cardiovascular risk factors, but did not significantly reduce the incidence of cardiovascular events, microvascular complications, and cardiovascular/overall mortality over the 5 year study period [12-14].

Pragmatic trials aiming to generate externally valid evidence in a real world setting, such as ADDITION, always present uncertainties concerning the implementation of the planned interventions in daily practice. Unlike highly controlled efficacy trials in which compliance to a (simple, one-dimensional) intervention can (and must) be assured, the purpose of pragmatic trials is to assess the effectiveness of a (complex, multifactorial) intervention in routine settings. In the ADDITION-Cambridge trial, intensive treatment (IT) was compared to routine care (RC) for screen detected diabetes patients. IT in ADDITION was a multifactorial intervention including treatment targets and treatment algorithms that were more intensive than those in contemporary UK national treatment guidelines, as well as educational material for patients [10; 15-17]. However, the degree to which protocol components were implemented into practice, and hence the degree to which more intensified treatment was actually provided to patients in the intervention arm, has remained unknown. Furthermore, potential changes in national treatment guidelines towards more intensive care, and the

For peer review only - http://bmjopen.bmj.com/site/about/guidelines.xhtml

introduction of the pay for performance system in England within the national Quality and Outcomes Framework (QOF) [18; 19], are likely to have improved routine care and may have diluted the difference in treatment intensity between the study arms over time [20].

Beyond improving understanding of the results of the ADDITION-Cambridge study, knowledge about whether and how the intervention was actually delivered in practice can inform future pragmatic trials in relation to barriers to protocol adherence, and the difference in treatment intensity that can be expected in a primary care based pragmatic trial in the context of background policy changes.

The objective of this study was therefore to describe the adherence of GPs to the trial protocol and to compare the intensity of care delivered to screen detected diabetes patients between the trial arms.

Methods

Study design

The ADDITION-Cambridge study protocol has been published elsewhere[10]. In brief, ADDITION-Cambridge is part of the ADDITION-Europe trial, which consisted of two phases: a screening program and a pragmatic, cluster-randomised trial comparing the effect of early intensive treatment versus routine care on five year cardiovascular risk in patients with screen-detected type 2 diabetes mellitus [9]. The primary endpoint was a composite of cardiovascular morbidity and mortality (cardiovascular death, non-fatal myocardial infarction, non-fatal stroke, non-traumatic amputations and revascularisations).

Study population

For ADDITION-Cambridge, 33,539 eligible individuals were invited to stepwise screening. Individuals eligible for screening were people registered at one of the participating general surgeries around Cambridge, aged 40 to 69 years, not known to have diabetes and with a diabetes risk score of >0.17 (corresponding to the top 25% of the population distribution). The risk score included age, sex, BMI, steroid and antihypertensive medication as well as smoking and family history [21]. Exclusion

For peer review only - http://bmjopen.bmj.com/site/about/guidelines.xhtml

criteria were assessed by the potential participant's GP. Patients with severe illness with a life expectancy of less than 12 months, those with psychological or psychiatric disorders that might invalidate informed consent and those who were housebound, pregnant or breast feeding were excluded from the study. 867 eligible patients (from n=49 surgeries) with screen detected diabetes participated in the pragmatic primary care based intervention trial. Ethical approval was granted by the Eastern Multi-Regional Ethics Committee (ref 02/5/54). Written informed consent was obtained from all participants. This trial is registered as ISRCTN86769081.

Due to the high expenses of assessing and extracting data from electronic primary care records it was decided in the planning phase of the ADDITION Cambridge study that only the records of a subset of the study will be assessed. It was decided that the records of participants with a primary endpoint within the 5 years of follow up plus the records of two random participants without a primary endpoint from the same GP surgery will be accessed. Consequently, the records of 63 participants with a primary endpoint (30 from the IT arm and 33 from the RC arm) and of 126 participants without a primary endpoint (60 from the IT arm and 66 from the RC arm) were collected. This selection procedure led in total to a subsample of 189 participants (IT: n=90 patients, RC: n=99 patients) from 34 surgeries (IT: 18 GP surgeries, RC: 16 GP surgeries). The study design is illustrated in detail in **figure 1**.

Intensive Treatment and Routine Care

Patients were treated according to the treatment allocation of their surgery. In the RC-arm patients received diabetes care through the National Health Service according to current UK guidelines and recommendations [15-17]. In the IT-arm additional features were added to current RC:

a) Surgeries received funding for 3 additional 10-minute GP consultations and 3 additional nurse consultations per year in the first 3 years after diagnosis.

b) Treatment algorithms were introduced along with underlying evidence demonstrating positive effects on CVD risk factors among patients with type 2 diabetes. In the IT-arm therapy with glucose lowering medication was indicated if $HbA_{1c} \geq 6.5\%$; ACE inhibitors/ARBs if BP $\geq$ 120/80mmHg;

For peer review only - http://bmjopen.bmj.com/site/about/guidelines.xhtml

statins if cholesterol ≥ 3.5 mmol/l; and aspirin for all patients independent of their risk factor levels (assuming that patients had no contraindications). The thresholds for treatment initiation for glucose lowering, BP lowering and lipid lowering medication and for aspirin therapy in both the IT-arm (based on the trial protocol [10]) and the RC-arm (based on national guidelines [15-17]) are summarized in **Table 1**.

Table 1:
Criteria for the initiation of glucose lowering, blood pressure lowering, lipid lowering and platelet inhibiting (aspirin) medication according to the trial protocol (IT-arm) and national guidelines (RC-arm) ǂ

	Glucose-lowering therapy	Blood pressure-lowering therapy	Lipid-lowering therapy	CVD risk-lowering aspirin therapy
**Routine Care (RC)**	- if HbA_{1c} ≥ 7% ˫	- if BP ≥ 160/100<br>- if 140/80 mmHg ≤ BP < 160/100 mmHg and either prevalent CVD or 10-year CHD risk ≥ 15%<br>*(ACE inhibitors, ARBs, B-blockers or diuretics as first choice)*	- if total cholesterol ≥ 5 mmol/l or triglycerides ≥ 2.3mmol/l<br>- if prevalent CVD or 10-year CHD-risk ≥ 15%	- if prevalent CVD or 10-year CHD-risk ≥ 15%
**Intensive Treatment (IT)**	- if HbA_{1c} ≥ 6.5%	if ≥ 120/80 mmHg or prevalent CVD<br>*(ACE inhibitors/ARBs as first choice)*	- if total cholesterol ≥ 3.5mmol/l	- independent of risk profile

ǂ Criteria are based on the national treatment guidelines from 2002 [15-17] and the ADDITION trial protocol [10]
˫ a range of 6.5% - 7.5% was mentioned. Consequently, the arithmetic mean of the borders (7%) was used as threshold

This figure does not claim to comprehensively describe the national treatment algorithms from the year 2002 or the detailed ADDITION trial protocol. It only highlights the differences in criteria for the initiation of drug therapy between IT and RC and does not account for possible contraindications.

c) Practice teams received theory-based educational materials to hand over to the patients, aiming to provide a shared framework for the management of their disease. Furthermore, GPs were advised to refer patients to a dietician and patients were encouraged through their GPs and nurses to increase their physical activity, to avoid excessive alcohol intake, to lose weight, to stop smoking, to adhere to medication, and to self-monitor blood glucose if given a glucometer by their GP.

Intensive treatment was promoted to participating surgeries by practice-based educational meetings with GPs and nurses. This included initial practice-based academic detailing conducted by a diabetologist and an academic GP to introduce treatment algorithms, and two interactive practice-based feedback sessions (approximately 6 and 14 months after the initial education session) to support and monitor treatment delivery.

For peer review only - http://bmjopen.bmj.com/site/about/guidelines.xhtml

Measures of treatment intensity

Information on the intensity of delivered care was extracted from the electronic primary care records of participating patients from the date of the diabetes diagnosis until December 2010 by a researcher blind to the GP surgery study group allocation. These files recorded the date and type of delivered services, including consultations with primary care health professionals, prescribed medications and laboratory measurements/tests. For the analyzed trial population more than 80,000 observations were available in the first 5 years after diagnosis. Clear text functions were used and algorithms were derived to classify the obtained information. Ambiguous observations were screened and coded by hand. Anatomic Therapeutic Chemical (ATC) codes were assigned to drugs to categorize medication classes. The intensity of care indicators were defined as follows:

Contact with health care professionals: The annual number of contacts between patients and GPs (including GP partners, GP principals, GP associates, out-of-hours doctors) and nurses (including practice nurses, nurse practitioners and nurse specialists). This included all contacts as we were unable to distinguish those related to diabetes alone.

Medication: Continuous treatment (≥ 4 prescriptions annually) with glucose lowering drugs (metformin, sulphonylurea, thiazolidinedione, insulin, other glucose lowering drugs), ACE inhibiting drugs (ACE inhibitors or ARBs), lipid lowering drugs (statins, other cholesterol lowering drugs) or aspirin.

Monitoring of risk factor levels: Regular monitoring of glycaemic control (≥ 2 HbA_{1c} tests per year), lipid profile (≥ 1 cholesterol test per year) and kidney function (≥ 1 urine albumin-creatinine ratio (UACR) test per year) [15-17].

Statistical Analyses

We analysed the difference in treatment intensity within the first 5 years from date of diagnosis. The study period was subdivided into five annual intervals representing year 1 (day 1 – day 365) to year 5 (day 1460 – day 1825) from diagnosis. 16 patients whose electronic primary care records did not contain information for at least one entire year were excluded from the analysis, resulting in an

For peer review only - http://bmjopen.bmj.com/site/about/guidelines.xhtml

analysis sample of 173 patients from 34 GP surgeries with a mean cluster size of 5 patients (IT: 82 patients from 18 surgeries, RC: 91 patients from 16 surgeries). Due to non-availability of data, surgery changes and deaths the total number of complete observed patient-years over the follow up period was 827 for contact with health care professionals and monitoring and 737 for prescriptions.

We applied linear regression models separately for years 1 to 5 in order to analyse the difference in the number of contacts with GPs and nurses for each individual year. A multi-level linear regression model accounting for repeated observations (year 1-5) within patients was applied to test the overall difference in the number of annual contacts between the study arms over the 5 year study period. This model included an interaction term between the year since diagnosis and the treatment to capture any time – treatment interactions.

In parallel with the linear regression models for the frequency of contacts with health care professionals, logistic regression models were applied to assess the likelihood of receiving continuous medication (≥ 4 prescriptions annually). In a secondary analysis, we also examined the likelihood of receiving regular monitoring of glycaemic control, lipid profile and kidney function and the likelihood of seeing a dietician[15-17].

Linear and logistic regression models were adjusted for age and sex and accounted for patients being clustered into surgeries (2-level model for stratified analyses and 3-level models for overall analyses). As the non-random selection of the analysed subsample does not exactly represent the study population, we tested in a sensitivity analysis if the introduction of a weighting factor (inverse probability of being included in the study based on the status of having a primary endpoint) has an impact on the results. We also altered the thresholds for the definition of 'continuous' medication (from 4 to 2, 6 and 12 prescriptions) to assess the sensitivity towards these threshold definitions. To assess the sensitivity to missing data we further refitted the analyses to a regression-based multiple-imputed (n=10 imputations) dataset (n=189 patients). Statistical analyses were performed with SAS 9.3 using the GLIMMIX, MI and MIANALYZE procedures (Cary, NC).

For peer review only - http://bmjopen.bmj.com/site/about/guidelines.xhtml

To gain a more detailed insight into the pattern of GPs' adherence to treatment algorithms, we further extracted clinical information including HbA_{1c}, BP, cholesterol, triglycerides, prevalent CVD (defined as MI or stroke) and 10-year modelled CHD risk (using the UKPDS risk engine V2) from the baseline, year 1 and year 5 examinations of the ADDITION study. Missing clinical values were imputed by the methods of last observation carried forward (LOCF) and first observation carried backwards (FOCB) to avoid shrinkage of the sample size. We calculated the proportion of patients who should have received medication, i.e. the proportion of patients whose clinical values exceeded the thresholds referred to in the trial protocol [10] and the national guidelines[15-17] (P [clinical value ≥ threshold]) and the proportion of patients who actually received at least one prescription in a time frame of 3 months after the date of the laboratory measurement (P [# of prescriptions ≥ 1]) (Table 1). We finally defined the adherence of GPs to the trial protocol/national guidelines descriptively as the proportion of patients who receive at least one prescription, out of those patients whose clinical values exceed the thresholds (P [# of prescriptions ≥ 1] | [clinical value ≥ threshold]).

Results

Baseline sample characteristics

Characteristics of the sample at baseline are shown in **Table 2**. The mean age of the sample was 62 years, 34% were female and 96% Caucasian. The biomedical characteristics of the comparison arms were balanced. No differences were observed between the full sample (n=189) and the analysis sample (n=173).

Table 2:
Baseline characteristics of the used subsample of ADDITION Cambridge

	Intensive Treatment	Routine Care
N	82	91
Female sex, n (%)	30 (36.6)	30 (30.3)
Caucasian ethnicity, n (%)	77 (93.9)	96 (97)
Age, mean (SD)	61.87 (7.28)	62.01 (6.81)
BMI [kg/m2], mean (SD)	33.6 (5.6)	33.8 (5.9)
Total cholesterol [mmol/L], mean (SD)	5.47 (1.12)	5.46 (1.22)
HDL cholesterol [mmol/L], mean (SD)	1.16 (0.32)	1.2 (0.31)
Systolic blood pressure [mm Hg], mean (SD)	143 (20.8)	143.8 (22.2)
HbA_{1c} [%], mean (SD)	7.84 (2.09)	7.27 (1.59)

For peer review only - http://bmjopen.bmj.com/site/about/guidelines.xhtml

SD: Standard Deviation, BMI: Body Mass Index, HDL: High Density Lipoprotein, HbA_{1c}: glycated haemoglobin; N: number of individuals included in the analysis sample

Contact with health care professionals

The adjusted mean number of annual GP and nurse contacts is graphically illustrated in **Figure 2**. We found no difference in the mean annual number of contacts with GPs (IT: 5.80, vs. RC: 5.15, β=0.65 [95%-CI: -0.95, +2.26] or nurses (IT: 5.34 vs. RC: 5.49, β = -0.15 [-1.77, +1.48]) and no statistically significant trend over time.

Medication

The proportion of GPs who regularly prescribed (≥ 4 times annually) glucose lowering and cardio-protective drugs and odds ratios for the likelihood of regular prescriptions are shown in **Figure 3**.

GPs in the IT-arm were 3.27 [95%CI: 1.81 to 5.93] times more likely to regularly prescribe glucose lowering medications compared to GPs in the RC-arm. However, this difference diminished over the follow-up period as more patients in the RC arm were also prescribed medication. Patients in the IT-arm also had a greater chance of being prescribed lipid lowering medication (OR=2.42 [1.30 to 4.51]) and ACE inhibiting drugs (OR=2.03 [1.13, 3.65]), which were, in contrast to routine care guidelines, the first choice BP lowering drug according to the trial protocol. But no significant difference was observed between the trial arms for the category of BP lowering drugs as a whole (including beta-blocker, diuretics etc.) (OR=1.41 [0.71, 2.80]) (**Appendix 1**). No significant difference was observed between the trial arms for prescription of aspirin. Overall in both treatment arms, the likelihood of patients receiving glucose lowering, ACE inhibiting and lipid-lowering medications increased from diagnosis to five year follow up.

Monitoring of risk factors

The proportion of patients receiving regular HbA_{1c} tests (≥ 2 annually, 45% of patients), lipid tests (≥ 1 annually, 55% of patients) and UACR tests (≥ 1 annually, 75% of patients) was low. No significant difference was observed between the treatment arms (HbA_{1c} tests: OR=1.56 [0.63, 3.83], lipid tests OR=1.53 [0.51, 4.60], UACR-test: OR=0.82 [0.34, 1.98]) (**Appendix 1**).

For peer review only - http://bmjopen.bmj.com/site/about/guidelines.xhtml

Sensitivity Analysis

Analyses of multiple-imputed datasets led to qualitatively and quantitatively similar results. Also the introduction of a weighting factor to account for non-random patient selection yielded comparable results. Using different thresholds for the definition of 'continuous medication' showed that the results for glucose and lipid lowering medications were not sensitive to threshold definitions. However, increasing the threshold number for lipid lowering drugs attenuated the respective OR considerably (**Appendix 2**).

Adherence to prescription algorithms

The proportions of patients who should have received medication according to national guidelines and the ADDITION trial protocol and the proportions of patients who actually received a prescription within 3 months following the assessment of bio-medical data are presented in *column 1 and column 2* of **Figure 4**: The black part in *column 2* represents the proportion of patients who received a prescription and whose clinical values exceeded the thresholds for medication prescription and the framed white part represents the proportion of patients who received medication although clinical values did not exceed the thresholds. Adherence to the prescription algorithms, i.e. the proportion of patients who received at least one prescription out of those patients whose clinical values exceeded the thresholds (P [# of prescriptions ≥ 1] | [clinical value ≥ threshold]) is shown numerically in the lower part of **Figure 4**.

Due to tighter algorithms in the trial protocol (IT-arm) than in the national guidelines (RC-arm) more patients in the IT-arm were eligible for glucose-lowering, BP lowering and aspirin therapy than in the RC-arm. However, despite lower cholesterol thresholds in the IT-arm compared to the RC-arm, treatment with lipid lowering medication was indicated in almost equal proportions of patients in the two treatment arms.

Glucose lowering drugs: In the first year, the adherence to the treatment algorithm was generally low, but considerably higher in the IT-arm than in the RC-arm. At year 5, 73% of patients in both treatment arms with an HbA_{1c} ≥ threshold-level received a prescription.

For peer review only - http://bmjopen.bmj.com/site/about/guidelines.xhtml

BP- lowering/ACE inhibiting drugs: In the IT arm, adherence to the guideline for prescription of ACE inhibiting medication increased from 41% at baseline to 77% at year 5. In the RC arm, guideline adherence for prescription of any BP lowering medication increased from 55% at baseline to 94% at year 5 and ‘prescription adherence’ to ACE inhibiting medication (ACE inhibitors were not mentioned in the guidelines to be the first line treatment in RC) increased from 28% at baseline to 64% at year 5 (not shown). Of note, a large proportion of patients in the RC arm with BP levels below the threshold were prescribed BP lowering medication.

Lipid lowering drugs: Adherence to the treatment algorithms increased in both treatment arms and was consistently better in the IT-arm. At year 5, most patients with clinical values greater than threshold-levels were treated (IT-arm 93%, RC-arm 81%).

Aspirin: The adherence to the trial protocol/guidelines was low, less than 50% of eligible patients in both treatment arms received aspirin.

Discussion

Summary

ADDITION is a large pragmatic primary care based trial aiming to promote intensive multifactorial treatment of patients with screen detected diabetes by GPs. Utilizing electronic primary care records of patients, this study shows that GPs in the IT-arm did not see their patients more often, but were more likely to regularly prescribe metabolic and cardio-protective drugs. Generally, GPs' adherence to prescription algorithms increased substantially in both trial arms over the 5 year follow-up period. Large time-treatment interactions for prescription of glucose lowering medication indicates that background changes in routine care might have diluted the difference in treatment intensity over time.

Contextual frame

Pragmatic (“effectiveness”) trials seek to produce externally valid results in order to inform the process of decision-making by policy makers [22-25]. However, unlike in explanatory (“efficacy”) trials, adherence to protocol is rarely tightly monitored and the degree to which the intervention is

For peer review only

For peer review only - http://bmjopen.bmj.com/site/about/guidelines.xhtml

implemented often remains uncertain. In the case of non-statistically significant results, this begs the question whether the intervention is *per se* not efficacious in the tested (heterogeneous) population, or whether the intended difference in treatment intensity was not big enough to detect any effects in the given sample size.

Lack of a difference in the intensity of treatment can be due to different reasons. Firstly, adherence of responsible health care professionals to the protocol might be low due to limited motivation, insufficient resources or lack of interest in the ongoing trial. To tackle this issue, in ADDITION-Cambridge, a detailed trial protocol was specified and the implementation of the protocol elements was incentivized by additional monetary resources and supported by an initial practice-based academic and two interactive feedback sessions[10].

Secondly, treatment delivered in everyday practice might differ from both guidelines and what happens in research-active practices. Not considering actual practice in routine care can result in intervention plans that fail to induce treatment differences between the trial arms. The choice of suitable interventions is therefore particularly challenging in multi-national trials like ADDITION, where guidelines or daily practice in countries might differ but a certain degree of intervention homogeneity is warranted[9].

Thirdly, policy changes, such as changes in the remuneration system and modifications in treatment guidelines, can intensify routine care, thus potentially diluting differences between the intervention and routine care arm. Long-term trials such as ADDITION are particularly susceptible to such influences. Between 2003 (~start of the study) and 2008/09 (~end of the 5 year analysis period) in the UK no new national diabetes treatment guidelines were released. However, in 2004 the Quality and Outcomes Framework (QOF) with its pay for performance system was launched [18] and extended in the following years. The QOF incentivised fulfilment of basic quality of care indicators by monetary resources and may have improved the quality of care for patients with various conditions, including diabetes [20; 26].

For peer review only - http://bmjopen.bmj.com/site/about/guidelines.xhtml

Principal findings

Our study shows that although surgeries in the IT-arm received monetary resources for additional consultations, GPs and nurses did not see their patients more often, nor were they more likely to perform regular HbA_{1c}, lipid or UACR tests. This result might be explained by the fact that the patients in the RC-arm already saw their GP/nurse on average 5-6 times a year, which is more than the average ~4 GP and ~2.5 nurse contacts per year for the general UK population [27]. Therefore the GPs (and indeed the patients) may have felt that this was sufficient to adequately monitor the condition. It also shows that monetary incentives might help to convince a reasonable number of surgeries to participate in long-term extensive trials such as ADDITION (46% of contacted surgeries agreed to join the study), but that financial incentives might not be successful in motivating GPs to further increase treatment intensity if it is already at a high level [10]. Qualitative interviews with the GPs about their perspectives on the intervention, as conducted in the screening phase of the ADDITION study [28], would have been a valuable add on to address this question. In contrast, our results indicate that the education sessions and feedback audits had a positive impact on the protocol adherence of GPs, as in general adherence to the treatment algorithms in the IT-arm was higher than adherence to the national guidelines in the RC-arm. This finding supports previous research that feedback loops can help to maximize guideline adherence in primary care [29; 30].

According to the clinical thresholds outlined in the trial protocol and the national guidelines, more patients in the IT-arm than in the RC-arm were eligible to receive glucose-lowering, BP-lowering and platelet-inhibiting drugs (**Figure 4**). This suggests that the ADDITION intervention was designed at an appropriate level for the context, as even with a hypothetical prescription adherence of 100% patients in the IT-arm should have received more intensive treatment than patients in the RC-arm.

Notably, a very high proportion of patients in the RC-arm already received BP-lowering medication at baseline, although in many cases their BP levels did not exceed thresholds. The finding of high BP-lowering prescription prevalence probably results from the fact that treatment with BP lowering medication was part of the risk-score used to identify high risk individuals eligible for diabetes screening in the first phase of the ADDITION trial [10]. There could be two reasons why many of the

For peer review only - http://bmjopen.bmj.com/site/about/guidelines.xhtml

patients who received BP-lowering prescriptions had no apparent clinical indication for treatment. On the one hand, these patients might have previously had uncontrolled BP levels, but treatment with BP lowering medication brought their BP under control. On the other hand, it is possible that the daily practice for BP control at this time was already much stricter than recommended by the guidelines. Independently of its origin, the initially high prevalence of BP-lowering medication in both trial arms might be the reason why we did not observe a difference in the proportion of patients prescribed BP lowering drugs. Consequently, the observed difference in ACE inhibiting drugs may be due to GPs switching from diuretics or beta-blockers to ACE inhibiting drugs, as recommended by the trial protocol.

The low adherence to recommendations concerning aspirin therapy observed in both trial arms is interesting, as this prescription behaviour could be interpreted as a general scepticism among GPs (and perhaps patients) towards the weak evidence of benefits of aspirin therapy for primary prevention of cardiovascular disease [6]. The results of subsequent large trials justify such scepticism [31; 32]. Alternatively, some patients may have obtained aspirin from the pharmacy without a prescription without this being noted in the electronic medical record.

Except for aspirin, adherence to prescription algorithms increased substantially over the follow-up period. We assume that this finding is triggered by the progression and duration of the disease and by general improvements in the overall quality of care over time, independently of disease progression [33]. The significant interaction between 'treatment' and 'time since diagnosis' for glucose lowering medication indicates changing treatment patterns in the RC-arm which might be triggered by policy changes, like QOF. However, due to methodological limitations (covariate co-linearity, power problems in stratified models) this question could not be adequately addressed with the available data.

Implications for the planning of future pragmatic trials

This study shows that the successful implementation of a pragmatic trial in primary care is possible, but there are issues that need to be considered. Namely, (1) a high standard of care in control GP surgeries questions the need for further intensification, (2) treatment of patients in the RC-arm that did

For peer review only - http://bmjopen.bmj.com/site/about/guidelines.xhtml

not reflect the national guidelines, and (3) background policy changes affecting quality of routine care. These issues need to be identified, considered and addressed when designing a pragmatic study or rolling out an intervention comprehensively [23; 24; 34]. The results further underline the potential importance of standard good practice in (pragmatic) trials. Methods such as initial academic detailing and repeated feedback sessions may be of great importance for the overall success of the study [24; 35]. In this context, more qualitative or quantitative implementation research may help to identify and test strategies that affect the adherence of health care professionals (and patients) [36].

Ideally, pragmatic trials of complex interventions should, if possible, be designed in a way that allows evaluation of the adherence of health care professionals to the trial protocol and of patients to the chosen treatment regimen. This study shows that the use of electronic primary care records is a promising approach for assessing the adherence of GPs. The obtained data are also useful for health economic research. In this particular example, the new primary care data can be used to update a previous analysis to reduce uncertainty in the cost-effectiveness of the intervention [37], a method consistent with an iterative approach to research and adoption decisions [38-40].

Implications for the interpretation of trial results

Intensified prescription algorithms were well implemented into practice. We found that prescription with glucose lowering, ACE inhibiting and lipid lowering drugs was higher in the IT-arm. The expected treatment effect resulting from this difference in medication could be interpreted as an area under the curve issue: The combination of the magnitude and the duration of the treatment difference can be expected to be the crucial driver of long-term effects. The extended follow-up of the UKPDS trial, which aimed to reduce diabetes related complications through tighter glucose and BP control, has shown that after the termination of the intervention, between-group differences in laboratory measurements disappeared [41-44]. However, the reductions in risk of micro- and macro-vascular complications persisted (or increased) for patients who had received tight glucose control, but not for patients who had received tight BP control [41; 42]. In ADDITION we observed a small but significant improvement in HbA_{1c}, BP and cholesterol levels in the IT-arm and a non-significant reduction in risk of the composite CVD endpoint (RR=0.83, p=0.12) over a 5 year time period [14].

For peer review only - http://bmjopen.bmj.com/site/about/guidelines.xhtml

This study shows that the proportion of patients receiving glucose-lowering drugs in each arm had equalised at the end of the 5 year observation period, suggesting that the differences in glycaemic control might disappear in the subsequent years. However, as a substantially greater proportion of patients in the IT-arm received ACE inhibiting and lipid lowering drugs, it can be assumed that differences in BP and lipids might be sustained. If between-group differences in treatment for blood pressure and lipids diminish so will the levels of risk factors. However, the CVD risk may remain lower due to legacy effects of earlier reductions in glucose and cholesterol. Given that the number of events will also increase over time, it may be that the ADDITION intervention will show a statistically significant effect in the long-term; the ten year follow up of ADDITION will quantify the long term effect of relatively small differences in treatment and risk factors observed in the first 5 years after diagnosis of diabetes by screening [14].

Strengths and limitations

To our knowledge, this is one of the first studies to comprehensively analyse the adherence of GPs to a pragmatic trial protocol in primary care. In contrast to self-reported information from patients, electronically stored primary care records provide a high degree of detail about all GP-based primary care services delivered to patients and are less susceptible to recall bias [45]. Through the linkage of clinical information from the trial measurements with information on prescriptions from the electronic primary care records, it was further possible to comprehensively describe and analyse the prescription adherence of GPs to the trial protocol and national guidelines.

However, we only had data from a subsample of the ADDITION-Cambridge trial-cohort with an oversampling of patients with a primary event during the follow-up period. As our weighted sensitivity analyses showed that this issue did not affect the results, the findings of this study are likely to be generalizable to the sample of GP surgeries who participated in the ADDITION trial. Nevertheless, the generalizability of results to average GP surgeries in the UK might be quite limited. In the experience of the authors, the practices that take part in research tend to be more organised and deliver better quality routine care than those declining to participate. This might lead to ceiling

For peer review only

effects for interventions, i.e. it appears to be hard to induce a difference in treatment intensity between RC and a more intensive treatment regimen.

Another limitation is that in our assessment of prescription adherence, we did not take into account possible contra-indications for medications as well as patients' views, and analysed the data from a rather non-situational, disease-orientated perspective [46; 47]. Shared decision making between the GP and the patient might reasonably lead to decisions that deviate from those in the protocol (and national guidelines). We therefore do not know if patients or GPs were the main determinants of protocol non-adherence. It is possible that patients did not agree to start medication or to come to the surgery more often. To completely understand the adoption of the intervention the patient's role also needs to be taken into account, which was impossible with the chosen approach. Also, with the given data we could not evaluate the fidelity of GPs handing over the educational materials to study participants, which were also part of the intervention.

Finally, although the accuracy of primary care records for GP-based services is known to be quite high, particularly for prescribed medication and laboratory tests, the handling, merging and extraction of free text data from numerous observations (~80,000) originating from different IT format systems is challenging and validation was not undertaken [45]. Consequently, it is possible that a small proportion of services might be misclassified, resulting in non-differential bias.

Conclusion

This study demonstrates that the successful implementation of long-term pragmatic trials in primary care is possible, but there are many obstacles especially during periods of significant change in routine care. The retrospective analyses of the electronic primary care records of participants in the ADDITION-Cambridge trial shows that intensive treatment was fairly well implemented into practice, suggesting that positive effects on cardiovascular morbidity and mortality might be expected in the long-term. Where possible, data needed to evaluate the fidelity of stakeholders to trial protocols should be collected routinely in future pragmatic trials as this information is invaluable for the interpretation of study results and for the planning of future studies.

For peer review only - http://bmjopen.bmj.com/site/about/guidelines.xhtml

Figure Legends

Figure 1:

<u>Title:</u> Study design

Figure 2:

<u>Title:</u> Adjusted mean number (and 95%-CI) of contacts with GPs and nurses according to Intensive Treatment (grey) and Routine Care (black); stratified from year 1 to year 5 after diagnosis

<u>Legend:</u>

Ɨ stratified linear regression models with a main effect for the intervention; adjusted for sex and age of diagnosis; accounted for patients being clustered within GP practices

˫ overall linear regression models with a main effect for the intervention and for time since diagnosis and an interaction term between intervention and time; adjusted for sex and age of diagnosis; accounted for patients being clustered within GP practices and observations being clustered in patients

ɫ n=827 observations (n=169 in year 1, n=167 in year 2, n=168 in year 3, n=164 in year 4, and n=159 in year 5)

Figure 3:

<u>Title:</u> Proportion of patients receiving at least 4 prescriptions according to Intensive Treatment (grey) and Routine Care (black), stratified for year 1- 5 after diagnosis Ɨ

<u>Legend:</u>

Ɨ stratified logistic regression models with a main effect for the intervention; adjusted for sex and age of diagnosis; accounted for patients being clustered within GP practices

˫ overall logistic regression models with a main effect for the intervention and for time since diagnosis and an interaction term between intervention and time; adjusted for sex and age of diagnosis; accounted for patients being clustered within GP practices and observations being clustered in patients

ɫ n=151 in year1, n=149 in year 2, n=150 in year 3, n=146 in year 4, and n=141 in year 5

n=737 observations (n=151 in year1, n=149 in year 2, n=150 in year 3, n=146 in year 4, and n=141 in year 5)

For peer review only

For peer review only - http://bmjopen.bmj.com/site/about/guidelines.xhtml

Figure 4

Title: Proportion of patients who should receive medication according to the ADDITION protocol and national guidelines and the proportion of patients who receive medication within a 3 month time period at baseline, year 1 and year 5 after diagnosis #

Legend:

# baseline, n=169; year 1, n=167; year 5, n=145

* i.e. medication indicated

Ɨ i.e. either well controlled patients or those receiving medication without indication

꜔ i.e. poorly controlled patients or those receiving indicated medication

ɟ Adherence with ADDITION protocol; ¥ Adherence with national guidelines

Conflict of interest statement

None of the authors has competing interests.

Data Sharing Statement

The access policy for sharing is based on the MRC Policy and Guidance on Sharing of Research Data from Population and Patient Studies. All data sharing must meet the terms of existing participants' consent and study ethical approvals.

Information on data and data requests can be found on http://epi-meta.medschl.cam.ac.uk/includes/addcam/addcam.html. In case of questions please contact datasharing@mrc-epid.cam.ac.uk.

For peer review only - http://bmjopen.bmj.com/site/about/guidelines.xhtml

Author contribution

ML, EW, CB and SG designed the concept for the paper. ML performed the statistical analysis, interpreted the data and drafted the manuscript. SG and CB were involved in collecting the data. EW provided statistical support. All authors critically revised the intellectual content of the manuscript and approved its final version.

Acknowledgements

We gratefully acknowledge the contribution of all participants, practice nurses and general practitioners in the ADDITION-Cambridge study (a full list of participating practices is given below) and Kit Coutts and Dr Rebecca Simmons for their contribution to data collection. We also acknowledge the contribution of the trial steering committee (Professors Nigel Stott (Chair), John Weinman, Richard Himsworth, and Paul Little) and Prof Jane Armitage and Dr Louise Bowman who adjudicated the endpoints.

Funding

ADDITION-Cambridge was supported by the Wellcome Trust (grant reference No G061895) the Medical Research Council (grant reference no: G0001164), National Health Service R&D support funding (including the Primary Care Research and Diabetes Research Networks), and the National Institute for Health Research. We received an unrestricted grant from University of Aarhus, Denmark, to support the ADDITION-Cambridge trial. Bio-Rad provided equipment to undertake capillary glucose screening by HbA1c in general practice. SG is a National Institute for Health Research (NIHR) Senior Investigator. The Primary Care Unit is supported by NIHR Research funds. SJG received support from the Department of Health NIHR Programme Grant funding scheme (RP-PG-0606-1259). EW is funded by the NIHR Cambridge Biomedical Research Centre. The views expressed in this publication are those of the authors and not necessarily those of the NHS, the NIHR, or the Department of Health.The Primary Care Unit and the Medical Research Council Epidemiology Unit at the University of Cambridge jointly coordinated the study. Aside from the authors, the ADDITION-Cambridge study team has included Rebecca Abbott, Amanda Adler, Judith Argles, Gisela Baker, Rebecca Bale, Ros Barling, Daniel Barnes, Mark Betts, Sue Boase, Sandra Bovan, Gwen Brierley, Ryan Butler, James Brimbicombe, Parinya Chamnan, Sean Dinneen, Pesheya

For peer review only - http://bmjopen.bmj.com/site/about/guidelines.xhtml

Doubleday, Justin Basile Echouffo-Tcheugui, Sue Emms, Mark Evans, Tom Fanshawe, Francis Finucane, Philippa Gash, Julie Grant, Wendy Hardeman, Robert Henderson, Susie Hennings, Muriel Hood, Garry King, Ann-Louise Kinmonth, Georgina Lewis, Christine May Hall, Joanna Mitchell, Richard Parker, Nicola Popplewell, A Toby Prevost, Emanuella De Lucia Rolfe, Richard Salisbury, Lincoln Sargeant, Rebecca Simmons, Stephen Sharp, Megan Smith, Stephen Sutton, Nicholas Wareham, Liz White, Fiona Whittle and Kate Williams. We also wish to thank the Cambridge University Hospitals NHS Foundation Trust Department of Clinical Biochemistry and the NIHR Cambridge Biomedical Research Centre Core Biochemistry Assay Laboratory for carrying out the biochemical assays and the following groups within the MRC Epidemiology Unit: data management (Adam Dickinson), information technology (Iain Morrison, Rich Hutchinson), technical (Matt Sims), and field epidemiology (Paul Roberts, Kim Mwanza, James Sylvester, Gwen Brierley, Jaimie Taylor). ADDITION-Cambridge practices: Acorn Community Health Centre, Arbury Road Surgery, Ashwell Surgery, Birchwood Surgery, Bridge Street Medical Centre, Brookfields & Cherry Hinton, Broomfields, Buckden Surgery, Burwell Surgery, Cambridge Surgery, Cedar House Surgery, Charles Hicks Centre, Chequers Lane Surgery, Clarkson Surgery, Cornerstone Practice, Cornford House Surgery, Cottenham Surgery, Cromwell Place Surgery, Dr Smith and Partner (Cambridge), East Field Surgery, Ely Surgery, Freshwell Health Centre, George Clare Surgery, Great Staughton Surgery, Harston Surgery, Health Centre (Eaton Socon), Hilton House, John Tasker House, Lensfield Medical Practice, Manea Surgery, Mercheford House, Milton Surgery, Nene Valley Medical Practice, Nevells Road Surgery, New Roysia Surgery, Northcote House Surgery, Nuffield Road Medical Centre, Orchard Surgery, Orchard House Surgery, Orton Medical Practice, Park Medical Centre, Paston Health Centre, Peterborough Surgery, Petersfield Medical Practice, Prior's Field Surgery, Queen Edith's Medical Practice, Queen Street Surgery, Rainbow Surgery, Ramsey Health Centre, Riverside Practice, Roman Gate Surgery, Rosalind Franklin House, South Street Surgery, Thaxted Surgery, The Health Centre (Bury St Edmunds), The Old Exchange, The Surgery Stanground, Townley Close Health Centre, Trumpington Street Medical Practice, Werrington Health Centre, York Street Medical Practice.

For peer review only - http://bmjopen.bmj.com/site/about/guidelines.xhtml

References

1. Morgan CL, Currie CJ, Peters JR: Relationship between diabetes and mortality: a population study using record linkage. Diabetes Care 2000;23:1103-1107
2. Narayan KM, Gregg EW, Fagot-Campagna A, Engelgau MM, Vinicor F: Diabetes--a common, growing, serious, costly, and potentially preventable public health problem. Diabetes Res Clin Pract 2000;50:S77-84
3. Fowler MJ: Microvascular and Macrovascular Complications of Diabetes. Clinical Diabetes 2008;26:77-82
4. Schunk M, Reitmeir P, Schipf S, Volzke H, Meisinger C, Thorand B, Kluttig A, Greiser KH, Berger K, Muller G, Ellert U, Neuhauser H, Tamayo T, Rathmann W, Holle R: Health-related quality of life in subjects with and without Type 2 diabetes: pooled analysis of five population-based surveys in Germany. Diabet Med 2012;29:646-653
5. Gaede P, Lund-Andersen H, Parving HH, Pedersen O: Effect of a multifactorial intervention on mortality in type 2 diabetes. N Engl J Med 2008;358:580-591
6. Gaede P, Vedel P, Larsen N, Jensen GV, Parving HH, Pedersen O: Multifactorial intervention and cardiovascular disease in patients with type 2 diabetes. N Engl J Med 2003;348:383-393
7. Clarke PM, Gray AM, Briggs A, Stevens RJ, Matthews DR, Holman RR: Cost-utility analyses of intensive blood glucose and tight blood pressure control in type 2 diabetes (UKPDS 72). Diabetologia 2005;48:868-877
8. Gray A, Raikou M, McGuire A, Fenn P, Stevens R, Cull C, Stratton I, Adler A, Holman R, Turner R: Cost effectiveness of an intensive blood glucose control policy in patients with type 2 diabetes: economic analysis alongside randomised controlled trial (UKPDS 41). BMJ 2000;320:1373-1378
9. Lauritzen T, Griffin S, Borch-Johnsen K, Wareham NJ, Wolffenbuttel BH, Rutten G: The ADDITION study: proposed trial of the cost-effectiveness of an intensive multifactorial intervention on morbidity and mortality among people with Type 2 diabetes detected by screening. Int J Obes Relat Metab Disord 2000;24:S6-11
10. Echouffo-Tcheugui JB, Simmons RK, Williams KM, Barling RS, Prevost AT, Kinmonth AL, Wareham NJ, Griffin SJ: The ADDITION-Cambridge trial protocol: a cluster -- randomised controlled trial of screening for type 2 diabetes and intensive treatment for screen-detected patients. BMC Public Health 2009;9:1471-2458
11. Li R, Zhang P, Barker LE, Chowdhury FM, Zhang X: Cost-Effectiveness of Interventions to Prevent and Control Diabetes Mellitus: A Systematic Review. Diabetes Care 2010;33:1872-1894
12. Sandbaek A, Griffin SJ, Sharp SJ, Simmons RK, Borch-Johnsen K, Rutten GE, van den Donk M, Wareham NJ, Lauritzen T, Davies MJ, Khunti K: Effect of early multifactorial therapy compared with routine care on microvascular outcomes at 5 years in people with screen-detected diabetes: a randomized controlled trial: the ADDITION-Europe Study. Diabetes Care 2014;37:2015-2023
13. Simmons RK, Sharp SJ, Sandbaek A, Borch-Johnsen K, Davies MJ, Khunti K, Lauritzen T, Rutten GE, van den Donk M, Wareham NJ, Griffin SJ: Does early intensive multifactorial treatment reduce total cardiovascular burden in individuals with screen-detected diabetes? Findings from the ADDITION-Europe cluster-randomized trial. Diabet Med 2012;29:1464-5491
14. Griffin SJ, Borch-Johnsen K, Davies MJ, Khunti K, Rutten GE, Sandbaek A, Sharp SJ, Simmons RK, van den Donk M, Wareham NJ, Lauritzen T: Effect of early intensive multifactorial therapy on 5-year cardiovascular outcomes in individuals with type 2 diabetes detected by screening (ADDITION-Europe): a cluster-randomised trial. Lancet 2011;378:156-167

For peer review only - http://bmjopen.bmj.com/site/about/guidelines.xhtml

15. McIntosh AHA, Home PD, Brown F, Bruce A, Damerell A, Davis R, et al. Clinical guidelines and evidence review for Type 2 diabetes: management of blood glucose. University of Sheffield, 2001.
16. McIntosh AHA, Home PD, Brown F, Bruce A, Damerell A, Davis R, et al. Clinical guidelines and evidence review for Type 2 diabetes: management of blood pressure. University of Sheffield, 2002.
17. McIntosh AHA, Home PD, Brown F, Bruce A, Damerell A, Davis R, et al. Clinical guidelines and evidence review for Type 2 diabetes: Lipids management. University of Sheffield, 2002.
18. Health and Social Care Information Centre. National Quality and Outcomes Framework Statistics for England - 2004-05. retrieved from http://www.hscic.gov.uk/catalogue/PUB01946. last accessed: 05.02.2015.
19. Health and Social Care Information Centre. Quality and Outcomes Framework - 2006-07. retrieved from http://www.hscic.gov.uk/catalogue/PUB05997. last accessed: 05.02.2015.
20. Campbell S, Reeves D, Kontopantelis E, Middleton E, Sibbald B, Roland M: Quality of Primary Care in England with the Introduction of Pay for Performance. New England Journal of Medicine 2007;357:181-190
21. Clarke PM, Gray AM, Briggs A, Farmer AJ, Fenn P, Stevens RJ, Matthews DR, Stratton IM, Holman RR: A model to estimate the lifetime health outcomes of patients with type 2 diabetes: the United Kingdom Prospective Diabetes Study (UKPDS) Outcomes Model (UKPDS no. 68). Diabetologia 2004;47:1747-1759
22. Singal AG, Higgins PDR, Waljee AK: A Primer on Effectiveness and Efficacy Trials. Clin Trans Gastroenterol 2014;5:e45
23. Patsopoulos NA: A pragmatic view on pragmatic trials. Dialogues in Clinical Neuroscience 2011;13:217-224
24. Eldridge S: Pragmatic trials in primary health care: what, when and how? Family Practice 2010;27:591-592
25. Roland M, Torgerson DJ: What are pragmatic trials? BMJ 1998;316:285
26. Khunti K, Gadsby R, Millett C, Majeed A, Davies M: Quality of diabetes care in the UK: comparison of published quality-of-care reports with results of the Quality and Outcomes Framework for Diabetes. Diabetic Medicine 2007;24:1436-1441
27. Health & Social Care Information Centre. Trends in Consultation Rates in General Practice - 1995-2009. retrieved from http://www.hscic.gov.uk/pubs/gpcons95-09. last accessed: 23.05.2015
28. Graffy J, Grant J, Williams K, Cohn S, Macbay S, Griffin S, Kinmonth AL: More than measurement: practice team experiences of screening for type 2 diabetes. Fam Pract 2010;27:386-394
29. Ivers N, Jamtvedt G, Flottorp S, Young JM, Odgaard-Jensen J, French SD, O'Brien MA, Johansen M, Grimshaw J, Oxman AD: Audit and feedback: effects on professional practice and healthcare outcomes. Cochrane Database Syst Rev 2012;13
30. Goff DC, Jr., Massing MW, Bertoni AG, Davis J, Ambrosius WT, McArdle J, Duren-Winfield V, Sueta CA, Croft JB: Enhancing quality of heart failure care in managed Medicare and Medicaid in North Carolina: results of the North Carolina Achieving Cardiac Excellence (NC ACE) Project. Am Heart J 2005;150:717-724
31. Belch J, MacCuish A, Campbell I, Cobbe S, Taylor R, Prescott R, Lee R, Bancroft J, MacEwan S, Shepherd J, Macfarlane P, Morris A, Jung R, Kelly C, Connacher A, Peden N, Jamieson A, Matthews D, Leese G, McKnight J, O'Brien I, Semple C, Petrie J, Gordon D, Pringle S, MacWalter R, Prevention of Progression of Arterial D, Diabetes Study Group DRG, Royal College of Physicians E: The prevention of progression of arterial disease and diabetes (POPADAD) trial: factorial randomised placebo controlled trial of aspirin and antioxidants in patients with diabetes and asymptomatic peripheral arterial disease. BMJ : British Medical Journal 2008;337:a1840

For peer review only - http://bmjopen.bmj.com/site/about/guidelines.xhtml

32. Ogawa H, Nakayama M, Morimoto T, et al.: Low-dose aspirin for primary prevention of atherosclerotic events in patients with type 2 diabetes: A randomized controlled trial. JAMA 2008;300:2134-2141
33. Filion KB, Joseph L, Boivin JF, Suissa S, Brophy JM: Trends in the prescription of anti-diabetic medications in the United Kingdom: a population-based analysis. Pharmacoepidemiol Drug Saf 2009;18:973-976
34. Glasgow RE: What Does It Mean to Be Pragmatic? Pragmatic Methods, Measures, and Models to Facilitate Research Translation. Health Education & Behavior 2013;40:257-265
35. Godwin M, Ruhland L, Casson I, MacDonald S, Delva D, Birtwhistle R, Lam M, Seguin R: Pragmatic controlled clinical trials in primary care: the struggle between external and internal validity. BMC Med Res Methodol 2003;3:28
36. Peters DH, Adam T, Alonge O, Agyepong IA, Tran N: Implementation research: what it is and how to do it. BMJ 2013;347:f6753
37. Tao L, Wilson EC, Wareham NJ, Sandbaek A, Rutten GE, Lauritzen T, Khunti K, Davies MJ, Borch-Johnsen K, Griffin SJ, Simmons RK: Cost-effectiveness of intensive multifactorial treatment compared with routine care for individuals with screen-detected Type 2 diabetes: analysis of the ADDITION-UK cluster-randomized controlled trial. Diabet Med 2015;6:12711
38. Wilson E, Abrams K: From Evidence Based Economics to Economics Based Evidence: Using Systematic Review to inform the design of future research. In *Evidence Based Economics* Shemilt I, Mugford M, Vale L, Marsh K, Donaldson C, Eds. London, Blackwell Publishing, 2010
39. Claxton K, Cohen JT, Neumann PJ: When is evidence sufficient? Health Aff (Millwood) 2005;24:93-101
40. Sculpher M, Drummond M, Buxton M: The iterative use of economic evaluation as part of the process of health technology assessment. J Health Serv Res Policy 1997;2:26-30
41. Holman RR, Paul SK, Bethel MA, Matthews DR, Neil HAW: 10-Year Follow-up of Intensive Glucose Control in Type 2 Diabetes. New England Journal of Medicine 2008;359:1577-1589
42. Holman RR, Paul SK, Bethel MA, Neil HAW, Matthews DR: Long-Term Follow-up after Tight Control of Blood Pressure in Type 2 Diabetes. New England Journal of Medicine 2008;359:1565-1576
43. Tight blood pressure control and risk of macrovascular and microvascular complications in type 2 diabetes: UKPDS 38. UK Prospective Diabetes Study Group. Bmj 1998;317:703-713
44. Intensive blood-glucose control with sulphonylureas or insulin compared with conventional treatment and risk of complications in patients with type 2 diabetes (UKPDS 33). UK Prospective Diabetes Study (UKPDS) Group. Lancet 1998;352:837-853
45. Byford S, Leese M, Knapp M, Seivewright H, Cameron S, Jones V, Davidson K, Tyrer P: Comparison of alternative methods of collection of service use data for the economic evaluation of health care interventions. Health Econ 2007;16:531-536
46. Juul L, Sandbaek A, Foldspang A, Frydenberg M, Borch-Johnsen K, Lauritzen T: Adherence to guidelines in people with screen-detected type 2 diabetes, ADDITION, Denmark. Scand J Prim Health Care 2009;27:223-231
47. Zoffmann V, Harder I, Kirkevold M: A person-centered communication and reflection model: sharing decision-making in chronic care. Qual Health Res 2008;18:670-685

For peer review only - http://bmjopen.bmj.com/site/about/guidelines.xhtml

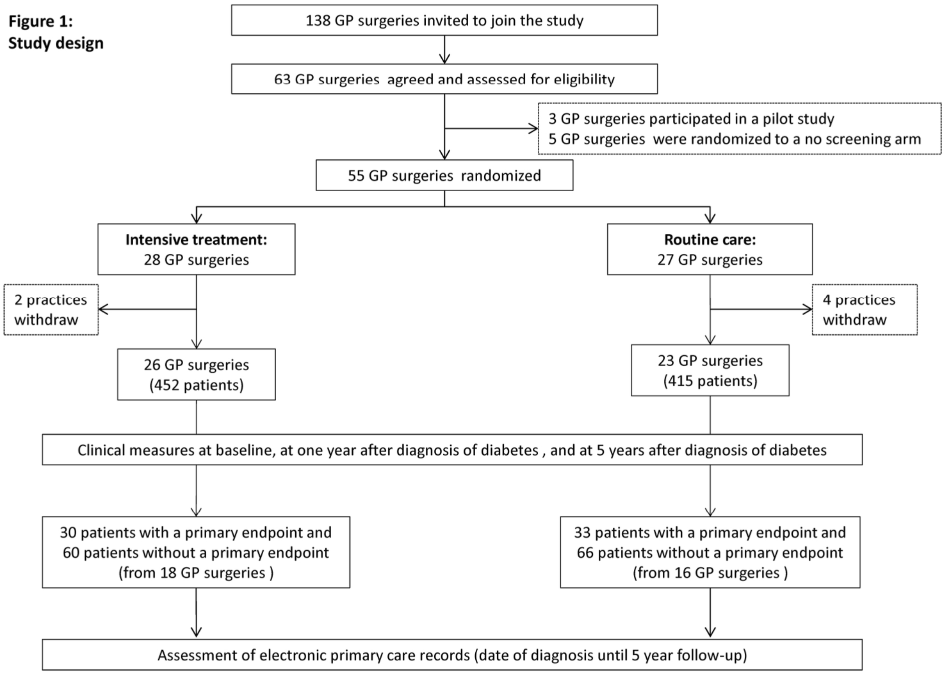

Figure 1

190x142mm (300 x 300 DPI)

ew only

Figure 2:

Adjusted mean number(and 95%-CI) of contacts with GPs and nurses according to Intensive Treatment (grey) and Routine Care (black); stratified from year 1 to year 5 after diagnosis ǂ

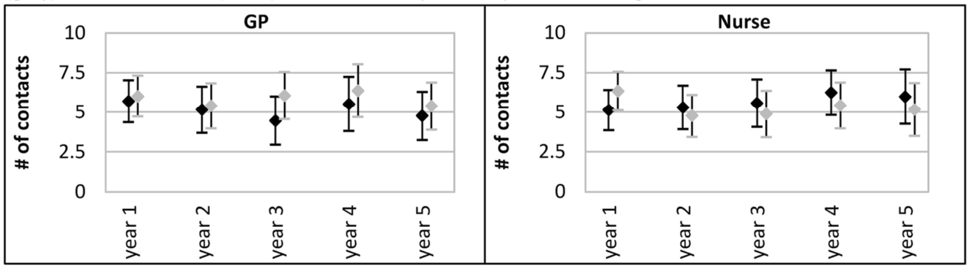

Overall adjusted mean number of contacts with GPs and nurses per year according to Routine Care and Intensive Treatment ⊦

	adj. mean (95%CI) ǂ		adj. mean (95%CI) ǂ
Intensive Treatment	5.80 (4.68, 6.93)	Intensive Treatment	5.34 (4.22, 6.47)
Routine Care	5.15 (4.01, 6.29)	Routine Care	5.49 (4.33, 6.65)
Difference (IT vs. RC)	0.65 (-0.95, 2.26)	Difference (IT vs. RC)	-0.15 (-1.77, 1.48)
time since diagnosis (years)	-0.05 (-0.24, 0.13)	time since diagnosis (years)	0.02 (-0.17, 0.21)
p-value (time x treatment)	0.513	p-value (time x treatment)	0.093

ǂ stratified linear regression models with a main effect for the intervention; adjusted for sex and age of diagnosis; accounted for patients being clustered within GP practices

⊦ overall linear regression models with a main effect for the intervention and for time since diagnosis and an interaction term between intervention and time; adjusted for sex and age of diagnosis; accounted for patients being clustered within GP practices and observations being clustered in patients

ǂ n=827 observations (n=169 in year 1, n=167 in year 2, n=168 in year 3, n=164 in year 4, and n=159 in year 5)

Figure 2

127x99mm (300 x 300 DPI)

Figure 3:
Proportion of patients receiving at least 4 prescriptions according to Intensive Treatment (grey) and Routine Care (black), stratified for year 1- 5 after diagnosis

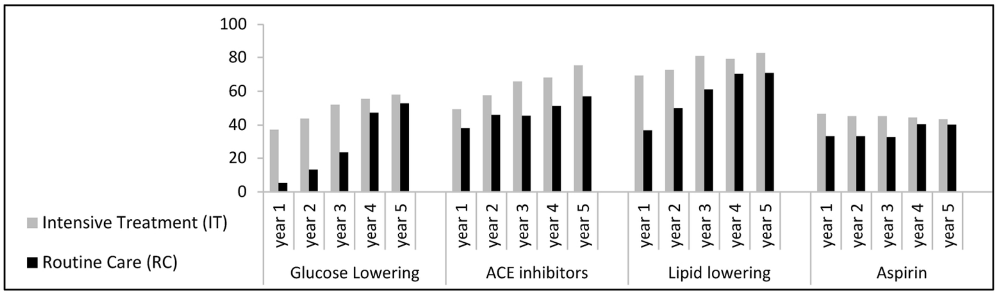

Odds Ratio of having received at least 4 prescriptions per year IT vs. RC (reference)

**Stratified by year ⱡ**	**OR (95%-CI) ⱡ**	**OR (95%-CI) ⱡ**	**OR (95%-CI) ⱡ**	**OR (95%-CI) ⱡ**
Year 1 (IC vs. RC)	10.89 (3.53, 33.56)	1.57 (0.73, 3.37)	4.00 (1.95, 8.20)	1.67 (0.72, 3.85)
Year 2 (IC vs. RC)	5.88 (2.51, 13.80)	1.60 (0.82, 3.09)	2.63 (1.31, 5.26)	1.66 (0.72, 3.86)
Year 3 (IC vs. RC)	3.78 (1.76, 8.10)	2.34 (1.18, 4.64)	2.63 (1.15, 6.01)	1.60 (0.62, 4.09)
Year 4 (IC vs. RC)	1.42 (0.73, 2.76)	2.06 (1.02, 4.14)	1.57 (0.68, 3.63)	1.16 (0.37, 3.61)
Year 5 (IC vs. RC)	1.23 (0.62, 2.42)	2.66 (1.14, 6.21)	1.99 (0.88, 4.53)	1.22 (0.43, 3.50)
**Year 1-5 ⊦**	**OR (95%-CI) #**	**OR (95%-CI) #**	**OR (95%-CI) #**	**OR (95%-CI) #**
Overall (IC vs. RC)	3.27 (1.81, 5.93)	2.03 (1.13, 3.65)	2.42 (1.3, 4.51)	1.41 (0.61, 3.24)
Time since diagnosis (per year)	1.61 (1.42, 1.83)	1.25 (1.12, 1.39)	1.33 (1.18, 1.5)	1.04 (0.93, 1.15)
p-value (time x treatment)	<.0001	0.331	0.131	0.220

ⱡ stratified logistic regression models with a main effect for the intervention; adjusted for sex and age of diagnosis; accounted for patients being clustered within GP practices

⊦ overall logistic regression models with a main effect for the intervention and for time since diagnosis and an interaction term between intervention and time; adjusted for sex and age of diagnosis; accounted for patients being clustered within GP practices and observations being clustered in patients

ⱡ n=151 in year1, n=149 in year 2, n=150 in year 3, n=146 in year 4, and n=141 in year 5

n=737 observations (n=151 in year1, n=149 in year 2, n=150 in year 3, n=146 in year 4, and n=141 in year 5)

Figure 3

159x130mm (300 x 300 DPI)

w only

Figure 4:
Proportion of patients who should receive medication according to the ADDITION protocol and national guidelines and the proportion of patients who receive medication within a 3 month time period at baseline, year 1 and year 5 after diagnosis [#]

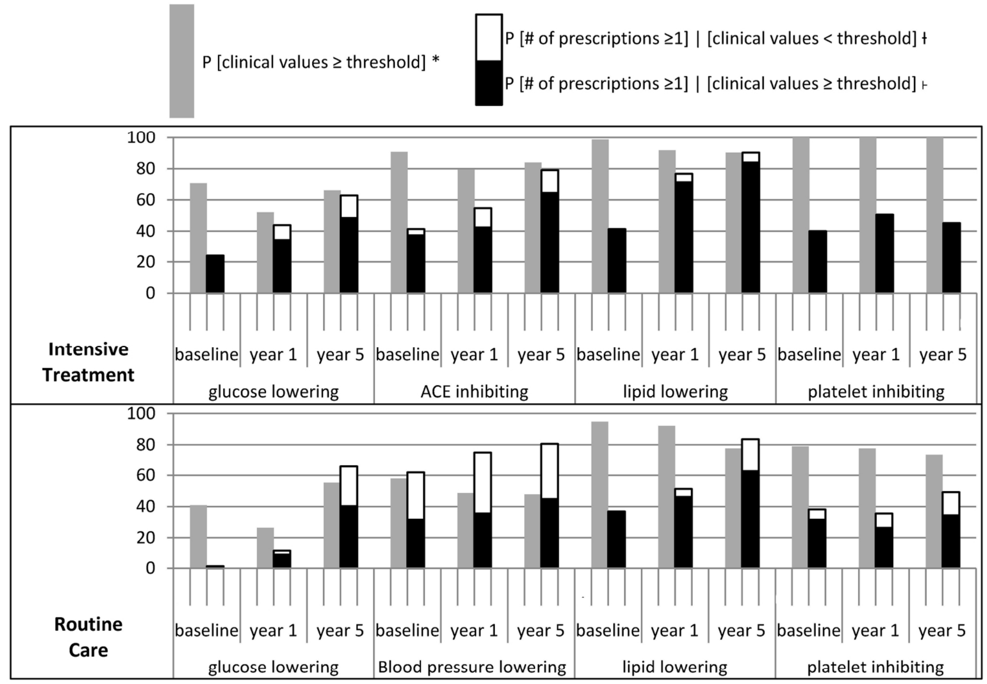

Prescription adherence (P [# of prescriptions ≥1] | [clinical value ≥ threshold])

IT-arm ⫞	0.32	0.66	0.73	0.41	0.53	0.77	0.42	0.78	0.93	0.40	0.51	0.45
RC-arm ¥	0.03	0.35	0.73	0.55	0.73	0.94	0.39	0.50	0.81	0.40	0.34	0.47

[#] baseline, n=169; year 1, n=167; year 5, n=145
* i.e. medication indicated
⸸ i.e. either well controlled patients or those receiving medication without indication
⊦ i.e. poorly controlled patients or those receiving indicated medication
⫞ Adherence with ADDITION protocol; ¥ Adherence with national guidelines

Figure 4

178x171mm (300 x 300 DPI)

Supplementary Material

Appendix 1: Proportion of patients receiving regular monitoring for HbA_{1c}, cholesterol and albuminuria and proportion of patients receiving blood pressure lowering medication

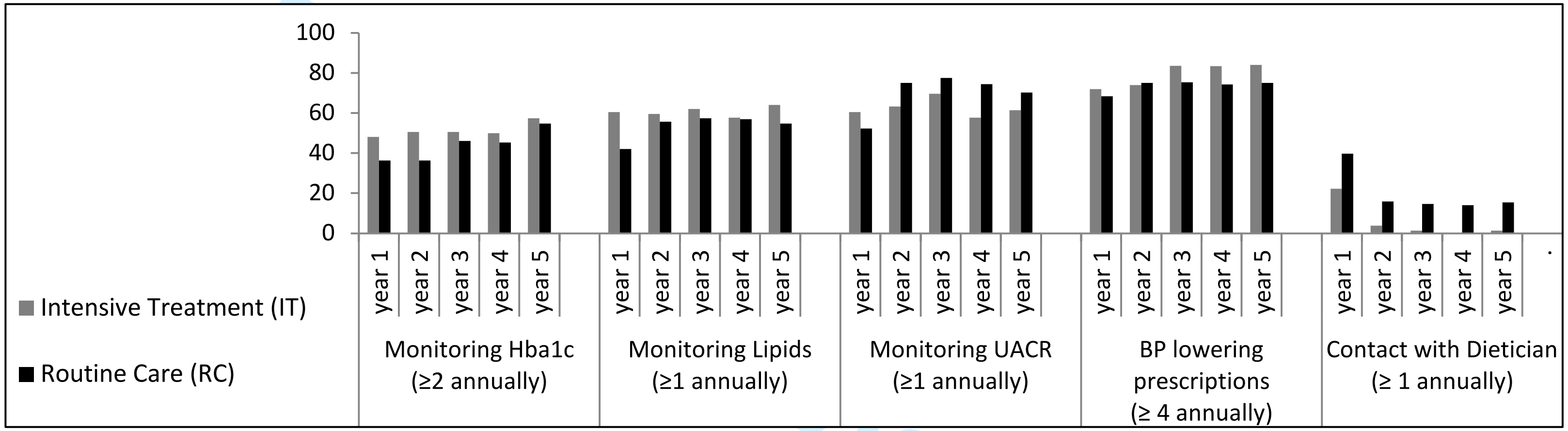

**Stratified by year ƚ**	**OR (95%-CI) ƚ**	**OR (95%-CI) ƚ**	**OR (95%-CI) ƚ**	**OR (95%-CI) #**	**OR (95%-CI) ƚ**
Year 1 (IT vs. RC)	2.00 (0.44, 9.02)	2.66 (0.53, 13.22)	1.41 (0.46, 4.32)	1.15 (0.55, 2.4)	0.88 (0.24, 3.26)
Year 2 (IT vs. RC)	2.29 (0.41, 12.63)	1.30 (0.23, 7.20)	0.62 (0.15, 2.60)	0.95 (0.45, 2.01)	0.38 (0.07, 2.18)
Year 3 (IT vs. RC)	1.28 (0.36, 4.52)	1.96 (0.36, 10.68)	0.93 (0.24, 3.56)	1.69 (0.72, 3.95)	0.12 (0.01, 1.39)
Year 4 (IT vs. RC)	1.52 (0.46, 5.03)	1.28 (0.29, 5.73)	0.49 (0.17, 1.45)	1.76 (0.72, 4.3)	-
Year 5 (IT vs. RC)	1.15 (0.44, 3.03)	2.3 (0.47, 11.32)	0.72 (0.30, 1.77)	1.89 (0.70, 5.15)	0.08 (0.01, 0.67)
**Year 1-5 ⊦**	**OR (95%-CI) ¬**	**OR (95%-CI) ¬**	**OR (95%-CI) ¬**	**OR (95%-CI) ⫞**	**OR (95%-CI) ¬**
Overall (IC vs. SC)	1.56 (0.63, 3.83)	1.53 (0.51, 4.6)	0.82 (0.34, 1.98)	1.41 (0.71, 2.8)	0.43 (0.32, 0.58)
Time since diagnosis (years)	1.12 (1.01, 1.23)	1.05 (0.95, 1.16)	1.08 (0.97, 1.2)	1.15 (1.02, 1.31)	0.13 (0.04, 0.45)
p-value (time x treatment)	0.294	0.303	0.075	0.223	0.001

ƚ stratified logistic regression models with a main effect for the intervention; adjusted for sex and age of diagnosis; accounted for patients being clustered within GP practices

⊦ overall logistic regression models with a main effect for the intervention and for time since diagnosis and an interaction term between intervention and time; adjusted for sex and age of diagnosis; accounted for patients being clustered within GP practices and observations being clustered in patients

ƚ n=169 in year 1, n=167 in year 2, n=168 in year 3, n=164 in year 4, and n=159 in year 5

\# n=151 in year1, n=149 in year 2, n=150 in year 3, n=146 in year 4 and n=141 in year 5

¬ n=827 observations (n=169 in year 1, n=167 in year 2, n=168 in year 3, n=164 in year 4, and n=159 in year 5)

⫞ n=737 observations (n=151 in year1, n=149 in year 2, n=150 in year 3, n=146 in year 4, and n=141 in year 5)

HbA_{1c} hemoglobin A1c; UACR urine-albumin-creatinine-ratio; BP blood pressure

For peer review only - http://bmjopen.bmj.com/site/about/guidelines.xhtml

Appendix 2: Results of various sensitivity analyses

	Adjusted odds ratio of having received 'continuous medication', IT vs. RC (reference)ƚ				Difference in adjusted mean number of contacts with GPs and nurses, IT vs. RC (reference) ⊦	
	**OR (95-% CI)**				**adjusted mean difference (95%-CI)**	
	Glucose-lowering	ACE-inhibiting	lipid-lowering	aspirin	# of GP contacts	# of nurse contacts
main model (from Figure 2 & 3)	*3.27 (1.81, 5.93) ¬*	*2.03 (1.13, 3.65) ¬*	*2.42 (1.30, 4.51) ¬*	*1.41 (0.61, 3.24) ¬*	*0.65 (-0.95, 2.26)˧*	*-0.15 (-1.77, 1.48) ˧*
a) weighted model	2.89 (1.51, 5.53) ¬	2.13 (1.15, 3.93) ¬	2.54 (1.32, 4.92) ¬	1.47 (0.59, 3.69) ¬	0.81 (-0.79, 2.42) ˧	0.21 (-1.40, 1.81) ˧
b) multiple imputed model	3.06 (1.78, 5.28) ǂ	2.05 (1.20, 3.50) ǂ	2.37 (1.32, 4.25) ǂ	1.32 (0.62, 2.80) ǂ	0.68 (-0.9, 2.26) ǂ	-0.10 (-1.70, 1.50) ǂ
c) threshold: ≥ 2 prescriptions annually	3.07 (1.68, 5.61) ¬	2.10 (1.12, 3.94) ¬	2.16 (1.13, 4.14) ¬	1.45 (0.70, 3.02) ¬	-	-
d) threshold: ≥ 6 prescriptions annually	3.97 (2.17, 7.26) ¬	2.24 (1.25, 4.03) ¬	2.35 (1.24, 4.45) ¬	1.40 (0.57, 3.46) ¬	-	-
e) threshold: ≥ 12 prescriptions annually	4.86 (2.34, 10.1) ¬	1.79 (0.79, 4.06) ¬	1.35 (0.58, 3.12) ¬	1.04 (0.37, 2.97) ¬	-	-

ƚ overall logistic regression models with a main effect for the intervention and for time since diagnosis and an interaction term between intervention and time; adjusted for sex and age of diagnosis; accounted for patients being clustered within GP practices and observations being clustered in patients

⊦ overall linear regression models with a main effect for the intervention and for time since diagnosis and an interaction term between intervention and time; adjusted for sex and age of diagnosis; accounted for patients being clustered within GP practices and observations being clustered in patients

¬ n=737 observations (n=151 in year1, n=149 in year 2, n=150 in year 3, n=146 in year 4, and n=141 in year 5)

ǂ n=885 observations (n=173 from year1 to year 5)

a) individuals weighted by the inverse probability of being in the sample given the status on the primary endpoint

b) multiple imputed dataset of participants with at least partially missing information on electronic primary care records in year 1 to 5 (PROC MI/PROC MIANALYZE)

c) threshold for 'continuous medication' changed to '≥ 2 prescriptions annually'

d) threshold for 'continuous medication' changed to '≥ 6 prescriptions annually'

e) threshold for 'continuous medication' changed to '≥ 12 prescriptions annually'

For peer review only

For peer review only - http://bmjopen.bmj.com/site/about/guidelines.xhtml

It needs to be acknowledged that the study does not report the primary or secondary outcomes of the trial (they have been reported elsewhere), but it reports the adherence of GPs to the trial protocol. Therefore, several points that are highly important in reporting the results of a trial are of inferior importance in reporting the adherence of GPs to the protocol.

CONSORT 2010 checklist of information to include when reporting a randomised trial*

Section/Topic	Item No	Checklist item	Reported on page No
**Title and abstract**			
	1a	Identification as a randomised trial in the title	1
	1b	Structured summary of trial design, methods, results, and conclusions (for specific guidance see CONSORT for abstracts)	3-4
**Introduction**			
Background and objectives	2a	Scientific background and explanation of rationale	5-6
	2b	Specific objectives or hypotheses	6
**Methods**			
Trial design	3a	Description of trial design (such as parallel, factorial) including allocation ratio	6-7
	3b	Important changes to methods after trial commencement (such as eligibility criteria), with reasons	Na
Participants	4a	Eligibility criteria for participants	6-7
	4b	Settings and locations where the data were collected	6-7
Interventions	5	The interventions for each group with sufficient details to allow replication, including how and when they were actually administered	7-8
Outcomes	6a	Completely defined pre-specified primary and secondary outcome measures, including how and when they were assessed	8-9
	6b	Any changes to trial outcomes after the trial commenced, with reasons	Na
Sample size	7a	How sample size was determined	6-7
	7b	When applicable, explanation of any interim analyses and stopping guidelines	Na
Randomisation:			
Sequence generation	8a	Method used to generate the random allocation sequence	Na
	8b	Type of randomisation; details of any restriction (such as blocking and block size)	Na
Allocation	9	Mechanism used to implement the random allocation sequence (such as sequentially numbered containers),	Na

For peer review only - http://bmjopen.bmj.com/site/about/guidelines.xhtml

concealment mechanism		describing any steps taken to conceal the sequence until interventions were assigned	
Implementation	10	Who generated the random allocation sequence, who enrolled participants, and who assigned participants to interventions	Na
Blinding	11a	If done, who was blinded after assignment to interventions (for example, participants, care providers, those assessing outcomes) and how	Na
	11b	If relevant, description of the similarity of interventions	na
Statistical methods	12a	Statistical methods used to compare groups for primary and secondary outcomes	9-10
	12b	Methods for additional analyses, such as subgroup analyses and adjusted analyses	9-10
**Results**			
Participant flow (a diagram is strongly recommended)	13a	For each group, the numbers of participants who were randomly assigned, received intended treatment, and were analysed for the primary outcome	Figure 1
	13b	For each group, losses and exclusions after randomisation, together with reasons	Figure 1
Recruitment	14a	Dates defining the periods of recruitment and follow-up	8
	14b	Why the trial ended or was stopped	Na
Baseline data	15	A table showing baseline demographic and clinical characteristics for each group	Table 1
Numbers analysed	16	For each group, number of participants (denominator) included in each analysis and whether the analysis was by original assigned groups	Figure 2-4
Outcomes and estimation	17a	For each primary and secondary outcome, results for each group, and the estimated effect size and its precision (such as 95% confidence interval)	Figure 2-4
	17b	For binary outcomes, presentation of both absolute and relative effect sizes is recommended	Na
Ancillary analyses	18	Results of any other analyses performed, including subgroup analyses and adjusted analyses, distinguishing pre-specified from exploratory	Na
Harms	19	All important harms or unintended effects in each group (for specific guidance see CONSORT for harms)	Na
**Discussion**			
Limitations	20	Trial limitations, addressing sources of potential bias, imprecision, and, if relevant, multiplicity of analyses	18-19
Generalisability	21	Generalisability (external validity, applicability) of the trial findings	18-19
Interpretation	22	Interpretation consistent with results, balancing benefits and harms, and considering other relevant evidence	Na
**Other information**			
Registration	23	Registration number and name of trial registry	4
Protocol	24	Where the full trial protocol can be accessed, if available	6 (ref 10)
Funding	25	Sources of funding and other support (such as supply of drugs), role of funders	20-21

For peer review only - http://bmjopen.bmj.com/site/about/guidelines.xhtml

*We strongly recommend reading this statement in conjunction with the CONSORT 2010 Explanation and Elaboration for important clarifications on all the items. If relevant, we also recommend reading CONSORT extensions for cluster randomised trials, non-inferiority and equivalence trials, non-pharmacological treatments, herbal interventions, and pragmatic trials. Additional extensions are forthcoming: for those and for up to date references relevant to this checklist, see www.consort-statement.org.

For peer review only

For peer review only - http://bmjopen.bmj.com/site/about/guidelines.xhtml
